# Elucidating the functional role of the novel BdP50 protein and extracellular vesicles in the human erythrocyte infection by *Babesia divergens*

**DOI:** 10.1371/journal.pntd.0013401

**Published:** 2025-08-13

**Authors:** Luis Miguel Gonzalez, Belen Revuelta, Aitor Gil, María C. Terrón, Martin Christoph Sachse, Javier Sotillo, Daniel Luque, S M Raihan Rahman, Reginaldo G. Bastos, Carlos Esteban Suarez, Estrella Montero

**Affiliations:** 1 Parasitology Reference and Research Laboratory, Centro Nacional de Microbiología, Instituto de Salud Carlos III, Majadahonda, Madrid, Spain; 2 Electron Microscopy Unit. Scientific and Technical Central Units, Instituto de Salud Carlos III, Majadahonda, Madrid, Spain; 3 Electron Microscopy Unit. Centro de Biología Molecular Severo Ochoa, CSIC-UAM, Campus de la Universidad Autónoma de Madrid, Madrid, Spain; 4 Electron Microscope Unit, Mark Wainwright Analytical Centre, University of New South Wales, Sydney, New South Wales, Australia; 5 School of Biomedical Sciences, University of New South Wales, Sydney, New South Wales, Australia; 6 Department of Veterinary Microbiology and Pathology, Washington State University, Pullman, Washington, United States of America; 7 Animal Disease Research Unit, USDA-ARS, Pullman, Washington, United States of America; Hokkaido University International Institute for Zoonosis Control, JAPAN

## Abstract

*Babesia divergens* is a blood-borne parasite that invades, replicates within and destroys red blood cells (RBCs) during its asexual life cycle, causing babesiosis in humans and cattle. This study focuses on BdP50, a putative *B. divergens* glycosylphosphatidylinositol-anchored protein involved in the parasite life cycle. BdP50 is found on the surface of *B. divergens* invasive parasites (merozoites) as well as on extracellular vesicles (*Bd*-derived EVs). These EVs are secreted by parasites cultured in fresh human RBCs and, in addition to BdP50, are enriched in human and parasite proteins, including proteins related to the parasite invasion process. BdP50 binds to RBCs and could mediate interactions of free merozoites and *Bd*-derived EVs with the host cell. Anti-BdP50 antibodies support this by blocking the BdP50 protein and inhibiting up to 88% of merozoite entry into naïve RBCs. This reinforces the role of BdP50 in parasite-host cell interactions and invasion. However, the inhibitory effect of BdP50 antibodies begins to gradually decrease slightly several hours after invasion, leading to a progressive increase in *B. divergens* infected RBCs over time. Consistent with these findings, our in vitro *de novo* infection assays showed that *Bd*-derived EVs, in addition to promoting parasite propagation, display proteins such as BdP50 that mimic the merozoite surface to likely attenuate the blocking effect of antibodies, thereby ensuring the parasite survival during subsequent rounds of invasion and growth. Given the role of BdP50 and *Bd*-derived EVs in the *B. divergens* life cycle*,* this study could have future implications for developing new approaches to interfere with parasite invasion proteins and *Bd*-derived EVs functions.

## Author summary

*Babesia divergens* parasites infect and destroy red blood cells (RBCs), causing severe babesiosis in humans and cattle. The in vitro model used to study *B. divergens* is based on culturing the parasite in human RBCs. Using this model, we have identified and characterized a glycosylphosphatidylinositol-anchored protein, BdP50. This protein localizes on the surface of free merozoites (the infective stage) and extracellular vesicles (*Bd*-derived EVs) secreted by *B. divergens* infected RBCs into the medium. In addition to BdP50, *Bd-*derived EVs contain abundant parasite and human proteins and interact with RBCs to prepare them for parasite invasion and growth. As BdP50 is capable of binding to RBCs, it is a strong candidate for mediating merozoites and EVs interactions with RBCs and for promoting invasion. Indeed, anti-BdP50 antibodies block 88% of merozoite invasion into RBCs at 12 hours post-invasion. However, parasite growth returns to normal within hours, suggesting an adaptive response. It is plausible that *Bd*-derived EVs, a key product secreted by parasites in vitro, play an important role in modulating the extracellular environment, to favor parasite propagation and to attenuate the blocking effect of antibodies, thereby promoting parasite survival. This study identifies BdP50 and *Bd*-derived EVs as key elements in the life cycle of *B. divergens*, shedding light on babesiosis and offering new insights into potential therapeutic interventions.

## Introduction

Protozoan parasites of the genus *Babesia* are considered a serious threat to humans, livestock and wildlife. *Babesia* parasites have a dixenous life cycle. They are naturally transmitted by the bite of ixodid ticks, and are capable of infecting the red blood cells (RBCs) of a wide variety of vertebrates, including humans [1].

*Babesia microti* and *Babesia divergens* are among the species that affect humans. *B. microti* is a worldwide species and causes endemic babesiosis in the United States and China [1]. *Babesia divergens* is the predominant species in Europe and causes asymptomatic to moderate infections and sporadic but life-threatening fulminant babesiosis, mainly in immunocompromised patients [2]. Clinical infections are characterized by fever, anemia, hemoglobinuria, renal failure and disseminated intravascular coagulation [1,2]. *B. divergens* also causes red water fever in cattle, which is characterized by progressive anemia, hemoglobinaemia and hemoglobinuria to which the disease owes its name [3].

In recent years, increasing attention has been paid to the impact of climate change on the geographical spread of vectors of *Babesia* spp. Climate change has clearly driven the northward spread of *Ixodes ricinus* ticks in Europe and *I. scapularis* ticks in North America. While human babesiosis has not yet shown a clear response to warming, predictive models suggest that it is only a matter of time before cases occur further than they do at present [4].

Clinical symptoms, particularly anemia—the canonical sign of babesiosis— [1] are directly associated with the kinetics of the asexual life cycle of *B. divergens* in the host bloodstream. Parasites invade the host RBCs and replicate asexually through binary fission, involving budding [5]. Then, parasites egress from RBCs as free merozoites, leaving the host cell irreversibly damaged. Free merozoites seek out and invade new RBCs to continue the asexual cycle causing inflammation and disease [6–8].

Several lines of evidence suggest that apical structures of the parasite, including micronemes, rhoptries, and dense granules, discharge many proteins onto the parasite surface, extracellular medium and the host cell in a rapid and coordinated process to facilitate the entry of the merozoite into the RBC [9–14]. Some of these key proteins, such as the apical membrane antigen BdAMA1, the subtilisin protease BdSUB1 and the rhoptry neck protein BdRON2 are blocked by anti-*B. divergens* antibodies, which partially inhibit merozoite entry into RBCs [12,14,15].

Another protein of interest is the major merozoite surface protein of *B. divergens* (Bd37). This glycosylphosphatidylinositol (GPI)-anchored protein is also involved in parasite invasion. Furthermore, Bd37 contributes to the protection of the parasite from the host immune response [13,16–18].

Overall, GPI-anchored proteins from protozoan parasites play crucial roles in host cell-pathogen interaction, invasion and immune evasion making them attractive targets for diagnostics, therapeutics and vaccines [19–23]. Despite their recognized importance in protozoan parasites, GPI-anchored proteins in *B. divergens*, with the exception of Bd37, are still poorly characterized.

In a previous functional genomic and transcriptomic analysis, we identified a novel *B. divergens* surface protein, which was named BdP50 [9]. In this study, bioinformatic predictions and functional analysis reveal that BdP50 is a putative GPI-anchored protein found on the merozoite surface as well as on extracellular vesicles secreted by *B. divergens* infected RBCs (*Bd*-derived EVs). The localization of Bdp50 together with its ability to bind RBCs, suggests that merozoites and *Bd*-derived EVs interact with the human RBC, via BdP50, promoting parasite invasion and growth. The findings highlight the biological significance of both BdP50 and *Bd*-derived EVs in the asexual life cycle of *B. divergens* and their potential as biomarkers for babesiosis.

## Materials and methods

### Ethics statement

Human A+ blood from healthy volunteer donors was used to maintain blood stage cultures of *B. divergens*. The blood and protocol were approved for use by the Blood Transfusion Center, Madrid, Spain. Donors provided informed written consent for use of their blood for research purposes. Rabbits were maintained and immunized in accordance with institutional and national guidelines. The protocol was approved by the Ethics Committee for Research and Animal Welfare (CEIyBA, Instituto de Salud Carlos III) and Consejería de Medio Ambiente y Ordenación del Territorio, Comunidad de Madrid, Spain (PROEX101–14001).

### *Babesia divergens* in vitro culture

*Babesia divergens* parasites (Bd Rouen 1987 strain) were cultured at 37°C, in a humidified atmosphere of 5% CO_2,_ in human A + RBCs using complete medium: RPMI 1640 medium (Life Technologies Corporation, Carlsbad, CA) supplemented with 10% human serum (HS, IBBI Grifolds, Memphis, TN), 0.25% (wt/vol) sodium bicarbonate solution (Life Technologies Corporation) and 50 mg/mL hypoxanthine (Sigma Aldrich, St. Louis, MO). Cells were stained with Giemsa and examined with a Primo Star microscope (Zeiss, Germany) at 100x magnification [24].

### *Babesia divergens* free merozoite isolation

Free merozoites were isolated from *B. divergens* culture flasks of 25 ml each at a parasitemia level of 25–40% [6]. Free merozoites were then pelleted at 2,000 g for 3 min) and resuspended in culture medium and immediately used for different assays.

### Bioinformatics analysis

Nucleotide sequences of cDNAs were compared to those deposited in the NCBI GenBank database using the BLAST algorithm (http://www.ncbi.nlm.nih.gov/BLAST, 27 November 2012). SignalP 6.0 server was used to predict the presence and location of signal peptide cleavage sites, PredGPI was used to predict GPI anchor and transmembrane domains in amino acid sequences [25,26] and FT-GPI was used to detect GPI-APs based on the presence of a hydrophobic helix at both ends of the premature peptide [27]. BUSCA server was used to predict subcellular localization of proteins. The 3D structures of BdP50 (CCX35043.1), *B. bovis* merozoite surface antigen-1 (BbovMSA1) (XP_001608956.1) and *B. bigemina* 45 kDa glycoprotein BbigGP45 (AEJ89908.1) were modelled using AlphaFold2 through Google Collaboratory [28,29]. The nuclear magnetic resonance (NMR) structure of delta-Bd37 structure (2lud) [18] and the of *B. canis* 28.1 (Bc28.1) (2lcu) [20], were derived from the Protein Data Bank (PDB). The structural superimposition and analysis were performed using ChimeraX [30]. The sequence alignment was done using Clustal Omega [31].

### Western blot analysis

Pellets of *B. divergens* infected RBCs (iRBCs) and free merozoite were collected by centrifugation and solubilized into three volumes of saponin buffer: 0.15% of saponin in phosphate-buffered saline (PBS) and incubated at 37°C for 20 min. Samples were then centrifuged for 5 min and pellets were resuspended in PBS with a protease inhibitor mixture (Sigma Aldrich) and sonicated. Thirty micrograms of protein extracts from *B. divergens* cultures, free merozoites and parasite supernatant, and 150 ng each of the recombinant proteins rBdP50Nt, rBdP50Ct and rBdP50 (S1 Appendix) were loaded, per well, on 12.5% SDS-PAGE gel. Western blot was also conducted on *Bd*-derived EVs, uRBC-derived EVs and *B. divergens* culture supernatants free of *Bd*-derived EVs. SDS-PAGE was achieved on 30 μg of protein for EVs and from 30 to 60 μg of protein for supernatants. Proteins were transferred to Amersham Protran nitrocellulose membranes (GE Healthcare) at 80 mA for 1 h, at room temperature (RT). Membranes were blocked in a blocking solution made of PBS with 0.05% Tween-20 (PBS-T) and 3% (w/v) bovine serum albumin (Sigma Aldrich) and incubated for 2 h, at RT, with anti-rBdP50Nt, -rBdP50Ct and/or -rBdP50 rabbit IgG antibodies (S1 Appendix) diluted 1:100 in blocking solution, according to each experiment. Then, membranes were treated with goat anti-rabbit IgG horseradish peroxidase (HRP) conjugate (Thermo Fisher Scientific, Rockford, IL) diluted 1:5,000 in blocking solution for 1 h, at RT, and washed with washing buffer: PBS + 0.05% Tween-20. Antigen detection was assessed by a colorimetric reaction (CN-DAB Substrate kit, Thermo Fihser Scientific) or by an enhanced chemiluminescence (ECL) substrate chemiluminescent reaction (SuperSignal West Femto kit, Thermo Fisher Scientific).

### Indirect immunofluorescent assay

To carry out an in-house indirect immunofluorescent assay (IFA), 1 × 10^7^ cells/ml from *B. divergens* iRBCs cultures and isolated free merozoite were pipetted onto 16-wells slides (10 μl per well) (Thermo Fisher Scientific) that were previously treated with 50 μl of concanavalin A (Sigma Aldrich) [5]. Slides were incubated at 37°C for 30 min and fixed in cold acetone–methanol (1:1) or 2% paraformaldehyde to maintain plasma membrane integrity after fixation. Then, slides were incubated at 37°C for 1 h with preimmune rabbit sera, anti-*B. divergens* supernatant sera or anti-rBdP50Ct antibodies diluted from 1:100–1:5,000 in PBS containing goat serum (10%). Slides were washed three times in PBS and incubated for 45 min with goat anti-rabbit IgG antibodies conjugated to fluorescein isothiocyanate (Thermo Fisher Scientific) diluted 1:200 in PBS containing goat serum (10%). The preparations were counterstained with 5 μl (2.5 μg ml^-1^ in PBS) of 4′, 6′-diamidino-2-phenylindole (DAPI) (Thermo Fisher Scientific) per well and examined with a Leica SP5 AOBS fluorescence confocal microscope (Leica Microsystems, Germany), using a 63 × oil immersion objective. High-resolution images were captured and processed with a Leica Application Suite Advanced Fluorescence (LAS AF) software (Leica Microsystems).

### Tokuyasu cryosectioning technique, immunolabelling and detection of BdP50 by transmission electron microscopy

*Babesia divergens* iRBCs and uninfected RBCs (uRBCs) were fixed for 2 h, at RT, in 2% paraformaldehyde and 0.1% glutaraldehyde in 0.1 M phosphate buffer pH 7.4. Fixed cells were washed with 50 mM NH_4_Cl in PBS to quench remaining free aldehydes and pelleted in 12% gelatin in PBS. The cell pellet was solidified on ice and cut into small blocks. For cryoprotection, blocks were infiltrated overnight with 2.3 M sucrose in PBS at 4°C, mounted on aluminium pins and frozen in liquid nitrogen. Thin cryosections were prepared with an UC7/FC7 (Leica Microsystems) and picked up with a 2:1 mixture of 2.3 M sucrose and 2% methylcellulose [32]. Thawed cryosections were labelled with preimmune and anti-rBdP50Ct sera followed by a goat anti-rabbit antibody coupled to 10 nm colloidal gold (British BioCell, UK). To block unspecific binding of antibodies 5% milk powder in PBS was used. Sections were contrasted with 0.4% uranyl acetate (EMS, Hatfield, PA) in 1.8% methylcellulose and observed with a FEI Tecnai 12 TEM equipped with a Ceta camera (Thermo Fisher Scientific) and operated at 120 kV. To assess the specificity of the anti-rBdP50Ct antibody labelling, we compared the number of gold-particles per surface area between the labelling of uRBCs and iRBCs. Images of uRBCs and iRBCs were taken at a nominal 15,000 x magnification. The surface area of uRBC and iRBCs was measured using the freehand selection tool of the Fiji software and the number of gold particles associated to both uRBCs and iRBCs was counted to determine the number of gold-particles per surface area.

### Isolation of extracellular vesicles

*Babesia divergens* parasites were grown using low human vesicle (LHV)-complete medium (S1 Appendix). Uninfected RBCs (uRBCs) were also maintained in LHV complete medium. When cultures, prepared in triplicated, reached parasitemia of 25–35%, the *B. divergens* supernatants and control uninfected supernatants were collected and sequentially centrifuged at 500 x g, 2,000 x g, and 3,500 x g for 15 min each at 4ºC. The supernatants were then concentrated using a 100 K Vivaspin filter (Sartorius, Gloucestershire, UK) at 6,000 x g and 4°C and spun at 100,000 x g for 6 h, at 4°C in a Beckman Coulter SW40Ti tube. Resulting pellets were washed with 0.22 µm-filtrated sterile PBS and spun again at 100,000 x g for 6 h at 4°C in a Beckman Coulter SW40Ti tube to obtain a pellet enriched in *Bd*-derived EVs and uRBC-derived EVs, respectively. Finally, pellets were resuspended in PBS and loaded on the top of commercial qEV70 nm Sepharose columns (iZON Sciences, Oxford, UK) pre-equilibrated with PBS. Five fractions of 500 μl each containing purified EVs were collected from columns. Protein concentration of EVs was determined by BCA assay (Thermo Fisher Scientific).

### Nanoparticle tracking analysis

The concentration and size of *Bd*-derived EVs and uRBC-derived EVs were analyzed by nanoparticle tracking analysis (NTA) using a NanoSight NS300 with a Blue 488 nm laser (Malvern Panalytical, Malvern, UK). Purified EVs were diluted 1:20 and 1:100 in 0.22 µm-filtrated sterile PBS to reach concentrations inside the precision range of the NTA machine (10^9^-10^11^ particles ml^-1^). Then, EVs were measured at camera levels 11–12 for each measurement, and three consecutive 60-s videos were recorded. After capture, the videos were analyzed (threshold 3) using the in-build NanoSight Software NTA 3.4 Build 3.4.003 (Malvern Panalytical, Worcester, UK).

### Extracellular vesicles proteomic analysis and data processing

*Bd*-derived EVs and uRBC-derived EVs were shipped to CBMSO Protein Chemistry Facility (Madrid, Spain) that belongs to ProteoRed, where protein identification and characterization were performed by LC-MS/MS (S1 Appendix). Database searches were performed using FragPipe (v19.1) with MSFragger (v3.8) [33] and Philosopher (v5.0) [34] against a concatenated target/decoy database consisting of the *B. divergens* proteome [9] appended to the *Homo sapiens* proteome from Uniprot (UP000005640; downloaded 4 April 2023) and common contaminants (25,191 proteins). Precursor and fragment mass tolerance were both set to 5 ppm and 0.01 Da, respectively, while mass calibration and parameter optimization were enabled, and isotope error was set to 0/1/2 with two missed trypsin cleavages allowed. Peptide length was set 7–50, and peptide mass range was set 500–5,000. Oxidation of M (+15.994915 Da) and acetylation of protein N-term (+42.010565 Da) were set as variable modifications, while Carbamidomethylation of C (+57.021464 Da) was set as fixed modification. MSBooster [35] and Percolator [36] were used for rescoring using deep learning prediction and PSM validation, respectively by using Philosopher. ProteinProphet [37] was used to estimate the identification FDR. Peptide and protein level FDR were set to 1%. The mass spectrometry proteomics data have been deposited to the ProteomeXchange Consortium [38] via the PRIDE [39] partner repository with the ProteomeXchange Accession: PXD052589 (https://www.ebi.ac.uk/pride/archive/projects/PXD052589) and FTP Download:https://ftp.pride.ebi.ac.uk/pride/data/archive/2025/07/PXD052589.

### Negative staining and transmission electron microscopy

For negative staining, *Bd*-derived EVs and uRBC-derived EVs were applied to glow-discharged carbon-coated grids (300 mesh) fixed for 5 min in 2% paraformaldehyde and stained with aqueous uranyl acetate. Then, EVs samples were then analysed on a FEI Tecnai 12 TEM equipped with a Ceta camera (Thermo Fisher Scientific) operated at 120 kV.

### Immuno-electron microscopy of extracellular vesicles

For immunogold labelling, *Bd*-derived EVs and uRBC-derived EVs were applied to glow-discharged collodion-carbon-coated copper grids and the grids were fixed with 2% paraformaldehyde. Fixed EVs were washed with 50 mM ammonium chloride in PBS to quench remaining free aldehydes. Then, grids were blocked with 1% BSA in PBS (blocking buffer) and incubated with anti-rBdP50Ct serum diluted 1:1,000 in blocking buffer for 30 min. Finally, grids were incubated for 30 min with a goat anti-rabbit antibody conjugated to 15-nm gold particles, washed and negatively stained with aqueous uranyl acetate. Images were recorded on a FEI Tecnai 12 transmission electron microscope (TEM) equipped with a Ceta camera (Thermo Fisher Scientific) and operated at 120 kV.

### *Babesia divergens*-derived extracellular vesicles efficiency in red blood cell invasion

*Bd*-derived EVs (1x10^9^ EVs ml^-1^) were added to warmed LHV-complete medium at 5% hematocrit (naïve RBCs), placed in 6-well plates and incubated for 18 h at 37°C in a humidified 5% CO2 atmosphere. Naïve RBCs were also incubated in triplicate in the absence of exogenous *Bd*-derived EVs. Then, 100 μl of free merozoite suspension was added to the pre-treated naïve RBCs in a 1:9 supernatant/cells (v/v) per well. Each well contained a final culture volume of 1 ml and the assay was set up in triplicate wells. Samples were incubated at 37°C in a humidified 5% CO_2_ atmosphere, and smears were prepared from each sample at 12 h and 24 h. The level of parasitemia was determined by counting the total number of intracellular parasites present in 2x10^3^ RBCs per slide at 100x magnification using a Primo Star microscope (Zeiss). Invasion/growth efficiency obtained was determined by comparing the observed levels of parasitemia for samples containing *Bd*-derived EVs to the level for samples free of exogenous *Bd*-derived EVs.

### Erythrocyte binding assay

Erythrocyte binding assay was conducted according to a previous protocol [20] with slight modifications: an increasing amount of packed RBCs, ranging from 50 to 200 μl, were incubated with the purified rBdP50 protein (0.5 mg ml^-1^ of protein in PBS) at RT, for 1 h with rotations. Experiments were also conducted with increasing amounts of rBdP50 protein (0.5, 1.0, and 1.5 mg ml^-1^ of protein in PBS) mixed with 100 μl of packed RBCs. The mixtures were then spun at 6,000 x g for 1 min through dibutyl phthalate to remove unbound material [14]. RBCs pelleted at the bottom of the tube, along with the recombinant proteins bound to them, and were recovered by puncturing the tube. Then, bound proteins were eluted from RBCs using 50 μl of PBS containing 0.5 M NaCl. Unbound and bound proteins were then analyzed by Western blot using mouse anti-His tag antibody diluted 1:200 (Qiagen). Membranes were treated with goat anti-mouse IgG HRP conjugate diluted 1:5,000 (Thermo Fisher Scientific). Antigen detection was performed using a CN/DAB Substrate Kit (Thermo Fisher Scientific). To ensure lack of nonspecific binding, the recombinant His-tagged fusion protein F18 (rF18) from *Taenia saginata* available in our laboratory [40], which is not expected to be able to bind to RBCs, was used as a negative control.

A similar binding assay was developed using aliquots of 500 μl of parasite supernatant and parasite supernatant free of EVs that were mixed with 100 μl of packed RBCs at RT, for 30 min [14]. The mixture was then processed as above and analyzed by Western blot using anti-rBdp50 antibodies. The antigen detection was performed using the ECL substrate chemiluminescent reaction (SuperSignal West Femto kit, Thermo Fisher Scientific).

### *In vitro* growth-inhibitory assay

Preimmune rabbit and anti-rBdP50 antibodies were purified using protein G-Sepharose (GE Healthcare) with IgG binding and elution buffers (Thermo Fisher Scientific), according to the manufacturer’s recommendation. Preimmune rabbit antibodies were used as a negative control. Each antibody was tested in triplicate. Controls without serum were also prepared. Preimmune and anti-BdP50 purified antibodies, at a final concentration of 2 mg ml^-1^ were added to warmed LHV-complete medium at 5% hematocrit (naïve RBCs) and deposited in 6-well plates. Then, 100 μl of free merozoite suspension was added to RBCs in a 1:9 supernatant/RBCs (v/v) per well. Free merozoites were allowed to invade RBCs for 5 min before RBCs were washed with RPMI to remove any uninvaded merozoite [15]. Samples for growth inhibition assays (GIA) were immediately replenished with the antibodies. Culture medium, containing purified IgG antibodies to a final concentration of 2 mg ml^-1^, was replenished at 12 h, 24 h and 36 h. Samples were incubated at 37°C in a humidified 5% CO_2_ atmosphere. Smears were made at 12 h, 24 h, 36 and 48 h. The slides were stained with Giemsa (Sigma Aldrich). The level of parasitemia was determined by counting the total number of intracellular parasites present in 2x10^3^ RBCs per slide at 100 x magnification using a Primo Star microscope (Zeiss). The level of inhibition of invasion with respect to the level of inhibition for the controls was determined for each antibody tested. The parasitemia of the no-serum control was considered 100% invasion/growth, and the level of inhibition of invasion/growth obtained by using of either purified preimmune serum or anti-BdP50 antibodies, was determined by comparing their observed levels of parasitemia to the level for the no-serum control.

### Statistical analysis

Comparisons between two treatments were performed with t-tests. Probability values (*p*) of 0.05 or less were considered statistically significant. Statistics and graphs were generated using GraphPad PRISM v.6 and Microsoft Office Excel 2016.

## Results

### The *Bd50* single copy gene encodes a GPI-anchored protein

In a previous work, we predicted a *B. divergens* surface protein named BdP50 in the genome and transcriptome of *B. divergens* Rouen 87 (GeneID: BDIVROU_0183000.t1.2; assembly accession number: GCA_001077455; BioProject PRJEB6536) [9]. Later, immunoscreening of a cDNA library using antibodies against proteins present in the supernatant of *B. divergens* in vitro cultures (S1A and S1B Fig) yielded 150 positive clones (S1 Appendix). One of the clones contained the BdP50 cDNA and expressed the BdP50 protein, confirming our genome and transcriptome prediction (S1 Table). The BdP50 cDNA sequence obtained from the clone (HF969321) was 100% identical to the BdP50 nucleotide sequence found in the *B. divergens* Rouen 87 genome and transcriptome [9]. A gene encoding BdP50 was also identified in the *B. divergens* strain 1802A genome [41]. Nucleotide BLAST analysis revealed no homologous sequences in other *Babesia* spp. Protein BLAST analysis revealed that the deduced BdP50 amino acid sequence showed low sequence identities of ~26% with the *B. gibsoni* 50 kDa surface antigen (accession no. BAB61953.1) and ~27% with the *B. gibsoni* immunodominant 47-kDa antigen (accession no. ACV83722.1) [42,43].

Complete nucleotide sequence analysis showed that the *Bdp50* is a single copy gene that contains no introns. The single contiguous cDNA sequence of 1,643-bp long contains an uninterrupted ORF of 1,347 bp encoding a protein of 448 amino acids with an estimated theoretical molecular mass of 48.15 kDa and an isoelectric point of 5.44 (S2 Fig). Using the full-length sequence of 448 amino acids, SignalP6 predicted a putative N-terminal (Nt) signal peptide 20 residues long, with the potential cleavage site between position V19 and C20 in the BdP50Nt. The signal peptide probability predicted by SignalP6 was 0.969085. PredGPI determined the presence and localization of the canonical omega (ω) site to which GPI anchor is attached at Ser 419, with the highest score = 1 [26]. The FT-GPI software predicted the presence of two strong transmembrane helices with a total score of 3,221. The N-terminal helix spanned residues 1–20 with a score of 1,506, while the C-terminal helix spanned residues 431–448 with a score of 1,715. These high scores support the presence of robust transmembrane domains, particularly at the C-terminus, fulfilling the criteria for predicted GPI-anchored proteins [27]. BUSCA further confirmed the presence of the Nt signal peptide and the C-terminal (Ct) signal for GPI attachment and classified BdP50 as a membrane protein.

Overall, GPI-anchored proteins are synthesized as preproproteins and undergo post-translational modifications before being transported to the parasite surface [44]. In line with the GPI-anchored protein biosynthesis pathway and the bioinformatics prediction, S2 Fig shows BdP50 as a preproprotein of 48.1 kDa containing the characteristic structural motifs of GPI-anchored proteins. The schematic representation also highlights the BdP50 proprotein and post-translational modifications sites, such as the proteolytic cleavage of the Nt and Ct motifs and the transfer of a GPI-anchor at the ω site. These modifications result in the formation of mature GPI-anchored protein with an estimated theoretical molecular mass of 43.3 kDa (S2 Fig).

Structural AlphaFold2 [28] analysis of BdP50, predicts the presence of Nt and Ct alpha helices, lacking beta sheets and disulfide bonds. The lack of disulfide bonds was consistent with the presence of a single cysteine residue in its primary structure (S3 Fig). Despite the low amino acid sequence identity, structural analysis revealed that the Nt helices of BdP50 overlapped with helices of the crystal structures of the GPI-anchored proteins Bd37 and Bc28.1 [18,20]. Moreover, the Ct helices of BdP50 overlapped with the AlphaFold2 predicted structures of the GPI-anchored proteins, BbovMSA1 and BbigGP45 (S3 Fig) [45–47].

### BdP50 is present in parasite lysates and supernatants

Sequences encoding Glu_21_–Leu_225_ and Lys_236_-Ser_418_, which excludes the Nt signal peptide and the Ct signal for GPI attachment, respectively, and the sequence encoding Glu_21_-Ser_418_, which excludes both Nt and Ct signal peptides were expressed in *E. coli* and purified as recombinant GST fusion proteins (rBdP50Nt- and rBdP50Ct) and as a His-tagged fusion protein (rBdP50), respectively (S2 Fig). The fusion proteins were used to produce different polyclonal sera (anti-rBdP50Nt, anti-rBdP50Ct and anti-rBdP50) in rabbits. All antisera reacted with the corresponding fusion proteins by Western blot (S1C Fig). All antisera were also tested by IFA, and the highest antibody titers were obtained for anti-rBdP50 and anti-rBdP50Ct rabbit sera. Based on these results, we selected polyclonal anti-rBdP50 and anti-rBdP50Ct antibodies for use in subsequent experiments.

**Fig 1:**
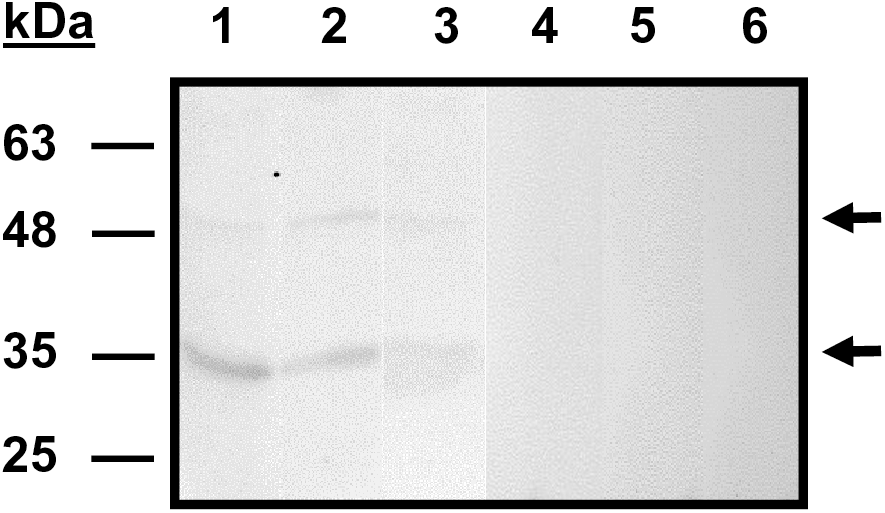
Identification of BdP50 in *B. divergens* cultures. Western blot using *B. divergens* free merozoites (lanes 1 and 4), *B. divergens* iRBCs (lanes 2 and 5) and *B. divergens* supernatants (lanes: 3 and 6) from in vitro cultures, and serum against rBdP50Ct (lines 1, 2 and 3) or preimmune serum, as negative control (lanes 4, 5 and 6). One band of ⁓48 kDa (p48) and a dominant band of ⁓35 kDa (p35) (marked with arrows) were identified by anti-rBdP50Ct antibodies in *B. divergens* free merozoites, iRBCs and supernatants. Molecular mass markers are shown on the left.

Western blot analysis using anti-rBdP50Ct polyclonal antibodies revealed cross-reactivity with *B. divergens* native proteins (Fig 1). A specific dominant product at ~35 kDa (p35) and a minor product at ~48 kDa (p48) were detected in *B. divergens* intraerythrocytic parasites, free merozoites and parasite supernatants preparations from in vitro cultures. As predicted by bioinformatics analyses, p48 was the largest detectable product or the BdP50-preproprotein whereas p35 represented the already processed BdP50 in the parasite culture (Fig 1). There was a good correlation between the theoretical molecular mass of the full-length BdP50 preproprotein (48.15 kDa) and the molecular mass of the ~ 48 kDa native product (p48). The predominant p35 final product of ~35 kDa was slightly smaller than the predicted molecular mass of 43.3 kDa Figs 1 and S2).

### BdP50-1 is found at the rhoptries and on the surface of *B. divergens* free merozoites

**Fig 2:**
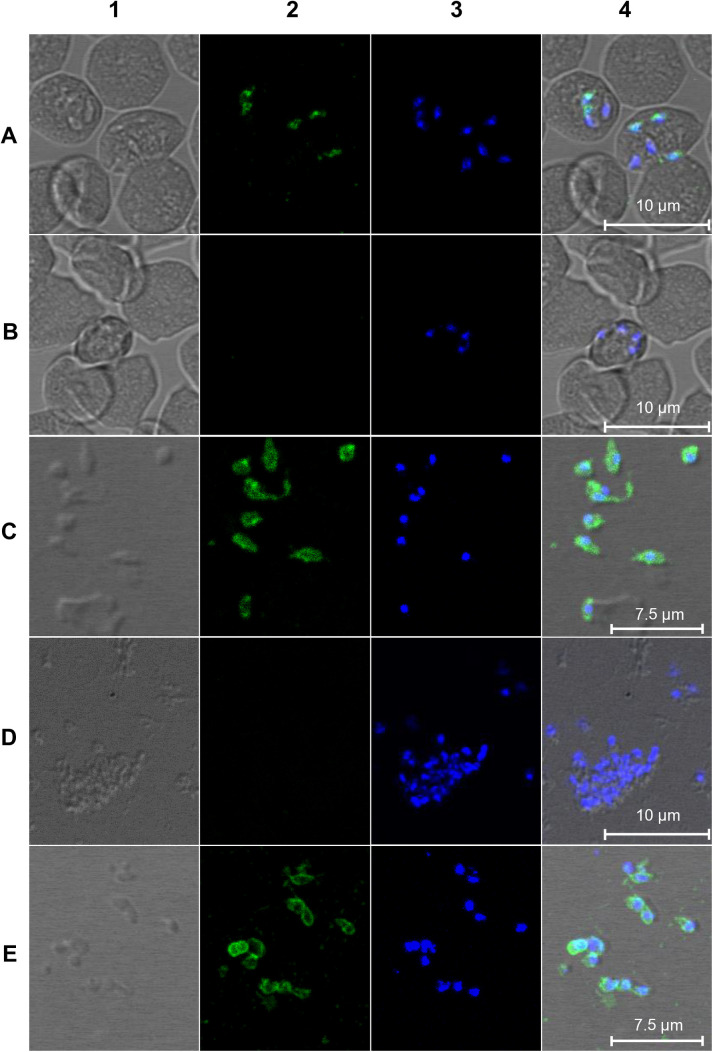
BdP50 localizes at the apical end and around the surface of *B. divergens* parasites. Immunofluorescent assays of intraerythrocytic and free parasites were performed by using anti-rBdP50Ct antibodies and preimmune rabbit serum as a negative control. Bound antibody was detected by using fluorescein isothiocyanate-conjugated anti–rabbit IgG antibodies. The preparations were counterstained with DAPI and examined by confocal microscopy. Samples were laser-stimulated at 488 and 405 nm. (A) Intraerythrocytic parasites were fixed with acetone-methanol (1:1) and probed with anti-rBdP50Ct antibodies. (B) Intraerythrocytic parasites were also probed with a preimmune rabbit serum. (C) Free merozoites were fixed with acetone-methanol (1:1) and probed with anti-rBdP50Ct antibodies. Free merozoites were also probed with preimmune rabbit sera. (E) Free extracellular merozoites were fixed with 2% paraformaldehyde and probed with anti-rBdP50Ct antibodies. Column 1 shows transmitted light images. Column 2 shows green fluorescent parasites. Column 3 shows the parasite DNA stained with DAPI. Column 4 shows all images overlaid.

To localize BdP50, IFA was performed on *B. divergens* iRBCs, as well as on purified, extracellular merozoites using anti-rBdP50Ct antibodies. In Fig 2, the staining pattern in intracellular parasites showed a punctate fluorescent signal, consistent with the apical end of the parasite. (Fig 2A, frames 2 and 4). When IFA was performed on free merozoites, a fluorescence signal was observed around the surface of the parasite in addition to the apical fluorescence signal using acetone-methanol (1:1) for fixation (Fig 2C, frames 2 and 4). In contrast, when using 2% paraformaldehyde in phosphate buffer for fixation, which preserves the integrity of the parasite membrane, the fluorescence signal appeared only around the surface of the free merozoite (Fig 2E, frames 2 and 4). TEM using the same antibody on Tokuyasu thawed cryosections of *B. divergens* iRBCs and free merozoites, confirmed the localization of BdP50. As shown in S4 Fig, low background labelling by anti-rBdP50Ct antibodies was observed in the cytosol of uRBCs (S3A Fig). In contrast, specific rBdP50Ct antibody reactivity was observed in *B. divergens* parasites inside iRBCs and free merozoites (S4B Fig). Quantitative analysis further showed a significant lower average density of gold particles on the surface area of uRBCs (0.4 gold particles/µm²) compared to iRBCs (4.6 gold particles/µm²), confirming the specificity of the rBdP50Ct antibodies (S2 Table). Consistent with the analyses, discrete labeling was seen in the bulb of the rhoptries at the apical end of intraerythrocytic parasites (S4C and S4D Fig). Moreover, BdP50 was found to be translocated from the rhoptries to the parasite surface, as strong immunoreactivity was observed on the surface of both intraerythrocytic parasites (S4C and S4D Fig) and free merozoites (S4 Fig, panels E and F). However, the mechanism and targeting signals involved in BdP50 trafficking to the surface remain unknown. The preimmune sera did not react with either uRBCs or iRBCs.

### BdP50 is also present in vesicles released by *B. divergens* parasites to the extracellular environment

As mentioned above, BdP50 was detected in culture supernatants by Western blot, suggesting that the protein is released into the medium by *B. divergens* parasites. Since parasites release both soluble proteins and protein-carrying extracellular vesicles (EVs) [48,49], we examined whether BdP50 is present in a soluble form or associated with *Bd*-derived EVs in the extracellular medium.

**Fig 3:**
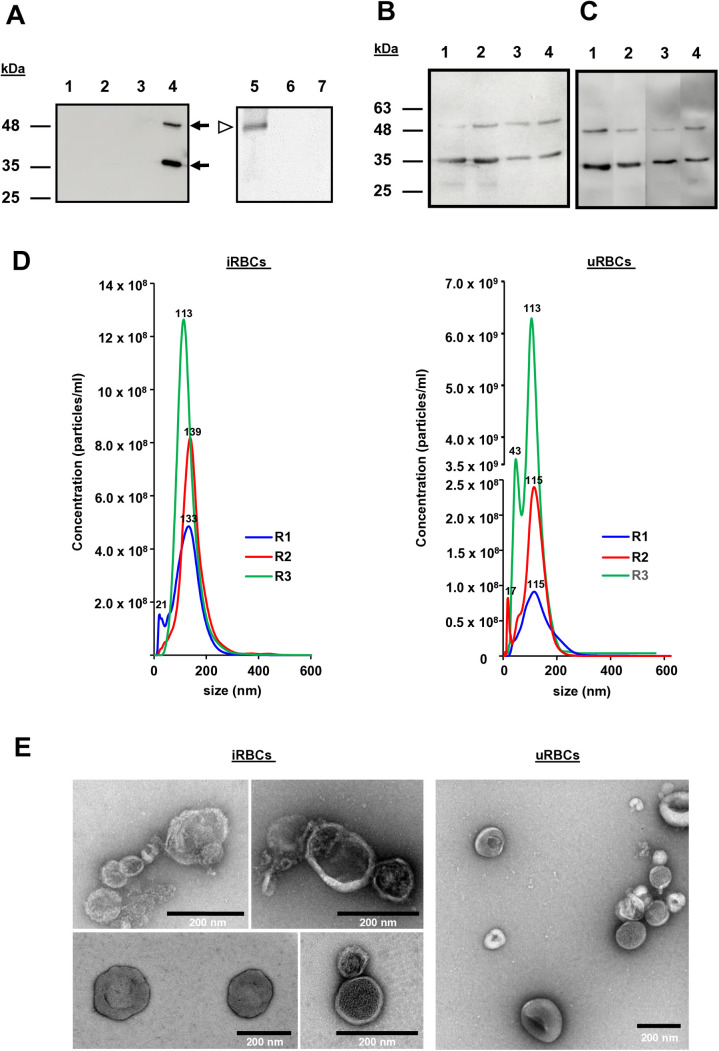
Characterization of *B. divergens* and human derived extracellular vesicles (EVs). (A) Immunolocalization of BdP50 by Western blot using uRBC-derived EVs and *Bd*-derived EVs as targets, preimmune rabbit serum (lanes 1 and2) and anti-rBdP50Ct antibodies (lanes 3 and 4). Lane 1 and 3: uRBC-derived EVs. Lane 2 and 4: *Bd*-derived EVs. The BdP50 protein is predominantly found at 35 kDa (p35) and as a minor band at 48 kDa (p48) in *Bd*-derived EVs (black arrows). Molecular mass standards are shown on the left. The presence of BdP50 as a soluble protein in parasite culture supernatants free of EVs is ruled out by Western blot using anti-rBdP50Ct antibodies. Lane 5: recombinant His-tagged protein rBdP50 (~43 kDa) was used as a control. Lanes 6 and 7: 30 and 60 μg of concentrated *B. divergens* culture supernatants free of EVs, respectively. *Babesia divergens* merozoites (B) and *Bd*-derived EVs (C) were incubated either without (lanes 1 and 3) or with phosphatidylinositol-specific phospholipase C (PI-PLC) for 15 min (lanes 2 and 4). After centrifugation, pellets and supernatants were collected and analyzed by SDS-PAGE followed by immunoblotting with anti-BdP50 antibodies. Lanes 1 and 3: pellets and corresponding supernatants from untreated samples. Lanes 2 and 4: pellets and corresponding supernatants from PI-PLC-treated samples. Molecular mass markers are indicated on the left. (D) Nanoparticle tracking analysis (NTA) of EVs that were purified by ultracentrifugation and subsequent size exclusion chromatography from culture supernatants of *B. divergens* iRBCs and uRBCs. NTA showed *Bd*-derived EVs with a modal size between 123 ± 2.2 nm and 137.5 ± 2.6 nm, and uRBC-derived EVs with a modal size from 112.6 ± 3.8 to 116.7 ± 2.8 nm. Profile of size (in nm) concentration is shown. The highest concentration peak of *Bd*-derived EVs was between ~113 - ~ 139 nm, whereas for uRBC-derived EVs it was between ~113 - ~ 115 nm. (E) Negative staining and visualisation by transmission electron microscopy of *Bd*-derived EVs and uRBC-derived EVs. The size ranges of the purified EVs were consistent with the nanoparticle tracking analysis shown in panel A. Scale bars: 200 nm.

To detect a soluble BdP50 form, *B. divergens* culture supernatants were collected, ultracentrifuged to remove the EVs, and then concentrated using centrifugal concentrators. Various amounts of EV-free concentrated supernatants were analyzed by Western blot, using the-rBdP50Ct serum. However, neither p35 nor p48 was detected in the assay, indicating that BdP50 is not present or detectable as a free soluble protein in the extracellular medium (Fig 3A–3C). We therefore isolated and purified *Bd*-derived EVs to analyze their protein content and to detect the presence of BdP50. In parallel, uRBC-derived EVs were also analyzed and compared to *Bd*-derived EVs.

To obtain the EVs secreted by iRBCs and uRBCs, we first removed the EVs present in the human serum used to supplement the culture medium for growing *B. divergens* (S1 Appendix). This step was taken to minimize the presence of human serum-derived EVs, thus avoiding overlap with the EV populations derived from iRBCs and uRBCs under study. To this end, human serum-derived EVs were depleted by ultracentrifugation, resulting in a human serum low in EVs (LHV-serum). *B. divergens* iRBCs and uRBCs were then cultured in triplicate with medium containing the LHV-serum, and the resulting supernatants were collected and used as a source of EVs.

*Bd*-derived EVs and uRBC-derived EVs were purified from each replicate by ultracentrifugation followed by size exclusion chromatography using qEV70 nm sepharose columns. Particles were fractionated into five batches. Fraction #2 consistently contained the highest portion of particles. The size range and the concentration of EVs from fraction #2 were determined by NTA. As shown in Fig 3D, NTA revealed EVs from *B. divergens* cultures ranging from size 20–300 nm, with the modal size between 123 ± 2.2 nm and 137.5 ± 2.6 nm, with peaks seen at 21, 113, 133 and 139 nm. EVs from uRBCs cultures yielded a slightly different mode of size range from 112.6 ± 3.8 to 116.7 ± 2.8 nm and picks at 17, 43, 113 and 115. Furthermore, the purified EVs were analyzed by negative staining, followed by TEM. As shown in Fig 3E, the approximate size and morphology of EVs were according with the NTA data. Variations in morphology and density among EVs were also observed, highlighting the heterogeneity of the EV population.

To assess the presence of BdP50 in *Bd*-derived EVs, we analyzed the EVs protein cargo by performing proteomics analysis. The proteins from *Bd*-derived EVs and uRBC-derived EVs replicates were extracted, trypsinized, separated by capillary liquid chromatography and analyzed by tandem mass spectrometry (LC/MS/MS) (S1 Appendix). The abundance profile of the *Bd*-derived EVs replicates was analyzed and compared. In total, 612 proteins were identified, of which 305 were *B. divergens*-derived proteins and the rest (307) were human-derived proteins. The EVs released by uRBC contained a total of 401 human proteins (S5 Fig).

*Bd*-derived EVs and uRBC-derived EVs shared 38% of the identified human proteins, the rest were unique to each group. Both *Bd*-derived EVs and uRBC-derived EVs contain several markers typically found in human EVs, including aldolase, fructose-bisphosphate A, Heat Shock Protein 90 Alpha Family Class A Member 1, Glyceraldehyde-3-Phosphate Dehydrogenase, Phosphoglycerate Kinase 1, Actin B, Cofilin 1, Clathrin Heavy Chain and programmed Cell Death 6 Interacting Protein [50–53], (S3, S4 and S5 Tables).

Among the human proteins present in *Bd*-derived EVs, many proteins were involved in metabolic, cell differentiation and metal ion binding processes or were identified as cytosol components. The most abundant functional related human proteins from uRBC-derived EVs were different from those present in *Bd*-derived EVs (S6 and S7 Figs and S3 and S4 Tables).

**Fig 4:**
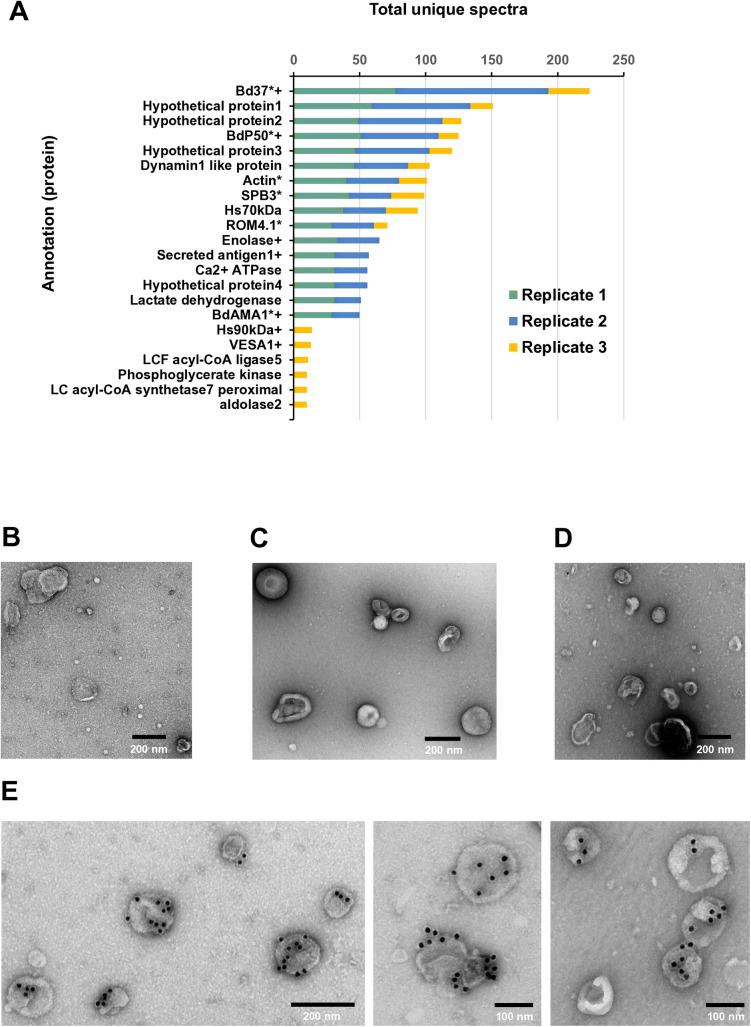
Most abundant *B. divergens* proteins in *Bd*-derived extracellular vesicles, including BdP50. (A) The graph shows the 16 most abundant parasite proteins found in *Bd*-derived EVs per replicate. (*) *B. divergens* proteins involved in the red blood cell invasion process (^+^) Molecules that were also identified by the immunoscreening of the *B. divergens* cDNA library. Immunoelectron microscopy analysis of uRBC-derived EVs and *Bd*-derived EVs in glow-discharged collodion-carbon-coated copper grids fixed with 2% paraformaldehyde. (B) uRBC-derived EVs that were immunolabeled with preimmune rabbit serum. (C) *Bd*-derived EVs that were immunolabeled with a preimmune rabbit serum. (D) uRBC-derived EVs that were immunolabeled with anti-rBdP50Ct antibodies. (E) BdP50 is exclusively localized in *Bd*-derived EVs that were immunolabeled with anti-BdP50Ct antibodies. Bound antibodies were detected with a goat anti-rabbit conjugated to 15-nm gold particles. Scale bars: 200 and 100 nm.

In relation to the parasite cargo, 78 *B. divergens* proteins were shared between the three *Bd*-derived EVs replicates. Forty-nine proteins from replicate *Bd*-derived EVs1 and 19 from replicate *Bd*-derived EVs2 were unique to each group (S5 Fig). *Babesia divergens* protein cargo included proteins related to biological process, molecular function and cellular components (S5 Fig). Of note, BdP50 was among the 16 more abundant proteins in replicates *Bd*-derived EVs1, EVs2 and EVs3, with Bd37 being the most abundant (Figs 4A and S8). Notably, Western blot confirmed the presence of both preprocessed and processed forms of BdP50, as indicated by the detection of p48 and the dominant p35 fractions in *Bd*-derived EVs (Fig 3A). Additionally, immunogold TEM performed on *Bd*-derived EVs fixed with 2% paraformaldehyde (PFA), without membrane permeabilization, confirmed the localization of BdP50 on the outer membrane of *Bd*-derived EVs (Fig 4B).

Free merozoites and *Bd*-derived EVs were treated with phosphatidylinositol-specific phospholipase C (PI-PLC) to evaluate the potential release of the BdP50 protein (S1 Appendix). Samples were incubated for 15–60 minutes, and the structural integrity of both merozoites and EVs was preserved following treatment, consistent with previous reports in *Toxoplasma gondii* tachyzoites [54]. However, as shown in Fig 3B and 3C BdP50 remained predominantly associated with the pellet fractions, which correspond to the recovered merozoites and EVs, regardless of treatment or incubation time. Comparable and consistently lower levels of BdP50 were detected in the supernatants of both treated and untreated samples. These findings indicate that BdP50 is not readily released by PI-PLC treatment.

Other proteins listed as more abundant in the three *Bd*-derived EVs replicates were: spherical body protein 3 (SBP 3), secreted antigen 1, BdAMA1, rhomboid like protease 4 (ROM4) and actin (S8 Fig), all of them involved in the *B. divergens* invasion process [9,14]. Although less abundant, other proteins related to invasion, gliding motility, moving junction formation and egress processes were also found in the replicates. These were: calcineurin, calcium dependent protein kinase 4 (CDPK-4), rhoptry neck proteins 2, 4 and 5 (RON2, RON4 and RON5), gliding associated proteins 45 and 50 (GAP45 and GAP50), myosin A (MYOA), profilin, thrombospondin related apical membrane protein (TRAP), RAP 1 and mac/perforin protein 2 (MAC2) [9,12,55]. Some of these invasion-related proteins were also identified through the screening of the *B. divergens* cDNA library by using the anti-*B. divergens* supernatant serum, which likely contains antibodies against both soluble proteins and EVs-associated proteins secreted into the extracellular medium (S8 Fig and S5 Table).

Proteomic data also revealed the presence of subunits of the 26S and 20S proteasomes in the *Bd*-derived EVs. Notably, the 20S proteasome has been recently identified in the EVs derived from many other pathogenic parasites, including *Plasmodium falciparum* where it is involved in reshaping host cell membranes to facilitate the malaria parasite invasion [53].

### *Babesia divergens*-derived extracellular vesicles promote parasite invasion and growth in human RBCs

**Fig 5:**
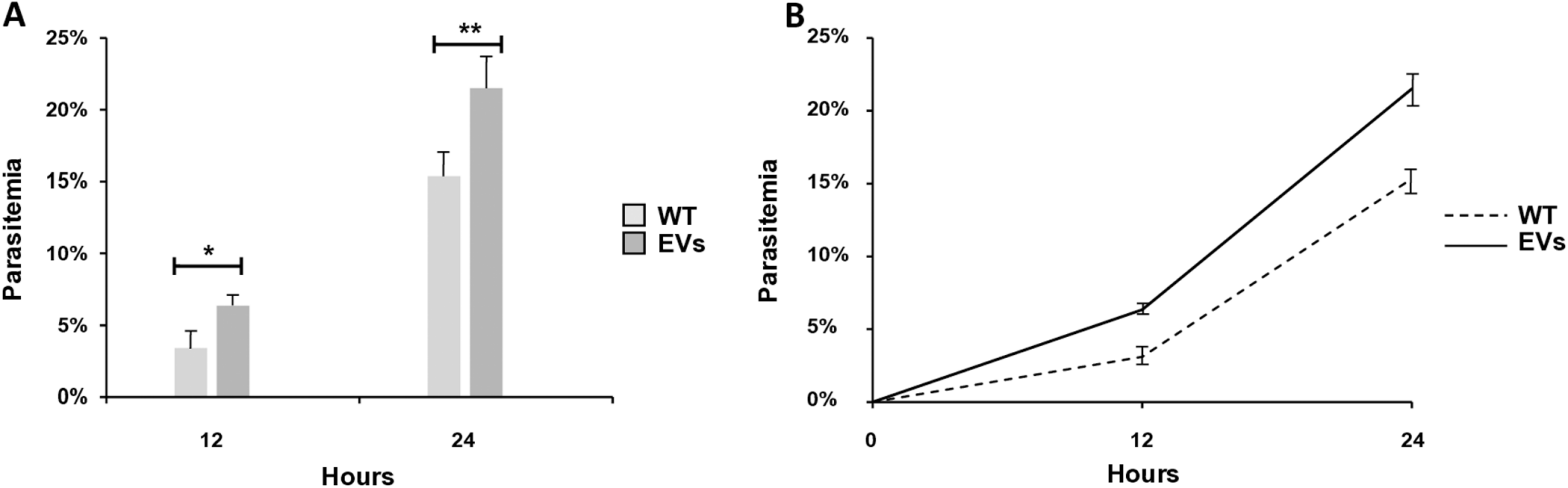
*Babesia divergens*-derived EV treatment favours parasite invasion and growth in human RBCs. Naïve RBC were pre-incubated for 18 h with *Bd*-derived EVs. Naïve RBCs were also incubated without exogenous *Bd*-derived EVs and used as a control. *Babesia divergens* free merozoites were then add to the pre-treated RBCs (with or without EVs) and parasitemia level was monitored using Giemsa-stained smears. Graphics A and B illustrate the significant differences in parasitemia between the group treated with *Bd*-derived EVs, which shows higher levels, and the group that did not receive any exogenous EVs. Each value represents the mean of the readings obtained from triplicates for each group. Error bars re*p*resent standard deviations of the mean levels of parasitemia, *p = 0.03, **p = 0.007*.*

In view of the presence of proteins in the *Bd*-derived EVs that are key in parasite invasion, we next examined whether EVs could interact with the human RBC to promote the invasion process. To this end, we followed the experimental approach described for the *P. falciparum*-derived EVs for similar purposes [53]. Based on previous findings that EVs from *B. divergens* cultures are internalized by host cells [48], naïve RBCs were pre-incubated for 18 hours with equal amounts of purified *Bd*-derived EVs (EV levels were counted by NTA measurement). Naïve RBCs were also incubated in the absence of exogenous EVs as a control. Free merozoites were then added to the pre-treated naïve RBCs and the control, and parasitemia levels were monitored for 12 and 24 hours by counting Giemsa smears of intraerythrocytic parasites. Our results indicated that in the naïve RBC group that received the *Bd*-derived EVs pre-treatment, parasitemia levels increased by 3.24% (*p* = 0.03) and 6.17% (*p* = 0.007) 12 and 24 hours post invasion, respectively, in comparison to the group that did not receive EVs (Fig 5). Thus, EVs derived from *B. divergens* cultures cooperated with naïve human RBCs to favor parasite propagation.

### BdP50 binds to human RBCs

**Fig 6:**
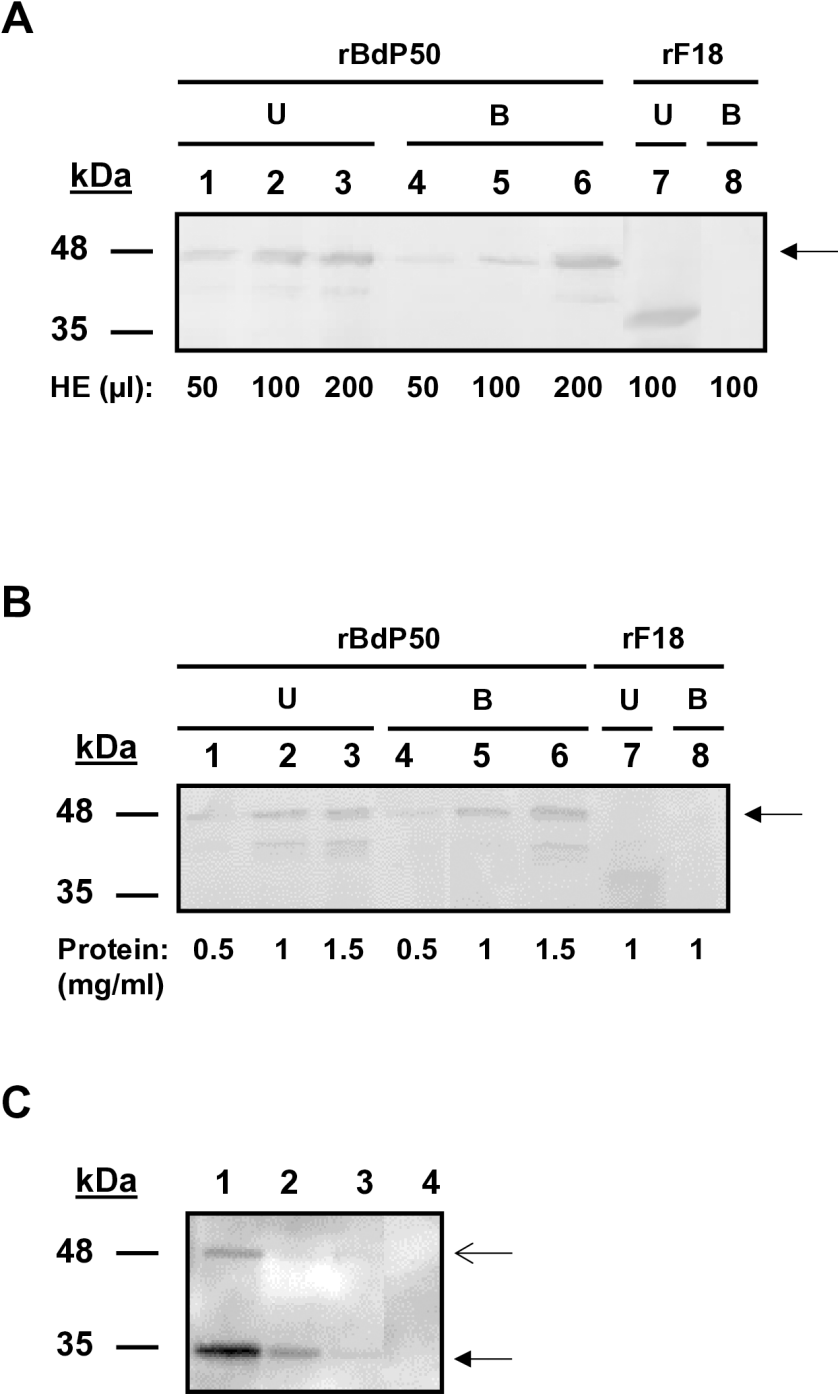
BdP50 is an erythrocyte-binding protein. (A) Binding assay using increasing amounts of packed human RBCs, ranging from 50 to 200 µl, and a constant amount of the recombinant rBdP50 protein (0.5 mg/ml) (black arrow). (B) Binding assay using increasing amounts of the recombinant rBdP50 protein (black arrow), ranging from 0.5 to 1.5 mg/ml, and a constant amount of packed RBCs (100 µl). Recombinant rF18 protein is used as a negative control. Lanes 1, 2, and 3 show unbound (U) rBdP50 protein and lanes 4, 5 and 6 bound rBdP50 protein, as revealed by Western blot using anti-His-tag monoclonal antibodies. The recombinant protein rF18 does not bind to human erythrocytes (lanes 7 and 8). (C) A binding assay mixing *B. divergens* culture supernatants or *B. divergens* supernatants free of EVs with packet RBCs was analyzed by Western blot using anti-rBdP50 antibodies*. Babesia divergens* culture supernatant (lane 1). Total eluate from RBCs after a binding reaction of the *B. divergens* culture supernatant (lane 2). Specific bound proteins (lane 3). BdP50 protein is found in *B. divergens* culture supernatant and eluate samples, predominantly at 35 kDa (p35) (black arrow) and as a faint minor band at 48 kDa (p48) (open arrow). The specific bound protein in lane 3 also corresponds to the major product p35. Specific bound proteins were not observed in *B. divergens* culture supernatants free of EVs (lane 4). Molecular mass markers are shown on the left.

The localization of BdP50 on the surfaces of both the free merozoite and the *Bd*-derived EVs suggested a potential role for BdP50 in RBC interaction and invasion. We therefore performed an erythrocyte-binding assay using increasing numbers of human RBCs and increasing amounts of the His-tagged fusion protein rBdP50. This is a functional assay that has been successfully used to evaluate the ability of other *Babesia* GPI-anchored proteins to bind RBCs [13,20]. As shown in Fig 6, rBdP50 bound to RBCs, indicating that the structured core of the protein is sufficient to ensure this function. Notably, the amount of recombinant protein that bound to RBCs was lower compared to the unbound protein, as in the case for Bd37 and Bc28.1 [13,20]. However, correct association was observed when the volume of packed RBCs was increased while the amount of rBdP50 remained constant, as an increasing amount of bound rBdP50 was recovered from the binding assay (Fig 6A). Furthermore, when the volume of packed RBCs remained constant and the amount of rBdP50 increased, an increasing amount of bound rBdP50 was also recovered from the binding assay (Fig 6B). A negative control was performed by using the rF18 protein [40], which did not bind RBCs (Fig 6A and 6B, lanes 7 and 8).

We conducted an additional functional binding assay [14] by mixing human RBCs with *B. divergens* culture supernatants as well as with parasite supernatants free of EVs. Binding was analyzed by Western blot using anti-rBdp50 antibodies. As shown in Fig 6C, BdP50 products, mainly the final p35 form, were detected in the binding assay using *B. divergens* culture supernatants. However, neither p35 nor p48 were detected in the binding assay when using parasite supernatants free of EVs. This result confirmed that BdP50 is not present as a free soluble protein in the extracellular medium (Fig 3A) but found in *Bd*-derived EVs to interact with RBCs.

### Anti-BdP50 antibodies inhibit red blood cell invasion in vitro

**Fig 7:**
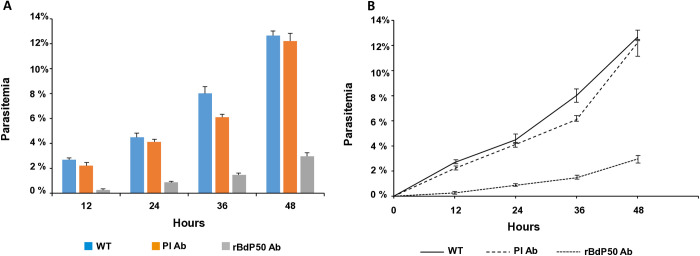
Purified anti-rBdP50 antibodies inhibit invasion of *B. divergens* merozoites in vitro. Naïve human RBC were infected by *B. divergens* free merozoites and parasitemia monitored at 12 h, 24 h, 36 and after 48 h post invasion in the continued presence of purified IgG antibodies from the anti-rBdP50 serum (rBdP50 Ab) or IgG antibodies from the preimmune serum (PI Ab) at 2 mg ml^-1^. An in vitro wild-type control (WT), free of IgG antibodies, was also monitored. The percentage of parasitemia was monitored by examination of Giemsa-stained smears. A and B) After 12 h, anti BdP50 Ab reach their maximum invasion inhibition of 88.2%, which is significantly higher than those achieved with PI Ab or in the control (no antibodies). Each value represents the mean of the readings (% parasitemia) obtained from triplicate samples for each antibody and control (WT). Error bars represent standard deviations of the mean levels of parasitemia.

Another important goal of the study was to evaluate BdP50 as a potential antigen for vaccine development. To this end, GIA was used to determine the efficacy of anti-rBdP50 antibodies in preventing de novo merozoite invasion. Efficient inhibition of parasite invasion was observed in cultures in the presence of anti-rBdP50 purified antibodies. The percentage of inhibition of merozoite invasion at 12 h post-RBC infection was significantly higher in the BdP50 antibody group (88.2% inhibition, *p* = 0,0057, 0.26% parasitemia) than in the IgG-purified antibodies from preimmune serum samples or in the no-antibody control. At 24 h, 36 h and 48 h the percentage of inhibition remained highly significant, but with a slight decrease as post infection time increased (78.9%, *p* = 0.0035, at 24 h; 75.9%, *p* = 0.0042, at 36 h and 73.9%, *p* = 0.0141 inhibition, at 48h). Conversely, the percentage of parasitemia at 24, 36 and 48 h increased slightly over time according to the observed parasitemia levels at 0.87%, 1.47% and 2.96%, respectively (Fig 7).

## Discussion

In this study, we have characterized the *B. divergens* BdP50 and substantially advanced our understanding of its biogenesis and cellular and extracellular localizations, which are associated with its role in host RBC interaction and invasion. We found BdP50 on the parasite surface, but also in *Bd*-derived EVs secreted by infected RBCs. EVs are lipid bilayer structures that facilitate cell-to-cell communication and, in the case of the pathogens, are used to transfer molecules into host cells, to signal to other pathogens and to modify the host environment to favor their survival [56]. The role of EVs in various diseases, including infectious diseases, is being actively investigated for potential diagnostic and therapeutic applications. Thus, in this work, we have also highlighted the functional relevance of the *Bd*-derived EVs on RBC infection and parasite propagation.

Genetic, proteomic and functional analysis provided evidence that BdP50 is synthesized as an early detectable preproprotein of ~ 48-kDa (p48), containing an Nt signal peptide and a Ct signal for GPI attachment. According to the canonical biogenesis pathway of GPI-anchored proteins, the p48 precursor undergoes remodeling steps and is translocated and distributed on the surface of intraerythrocytic parasites and free merozoites. During this maturation process, BdP50 appears to be processed into a final product of ~ 35 kDa (p35), which represents the major active form of BdP50 on intraerythrocytic parasites and free merozoites surfaces. The p35 form, smaller than its predicted theoretical mass of 43.3 kDa, resembles proteins in Apicomplexan parasites that are initially synthesized as high molecular weight molecules. These proteins undergo posterior maturation process and proteolytic cleavage, resulting in smaller, active forms on the parasite surface that differ from their predicted theoretical sizes [12,14,57,58].

In addition to being present on the surface of merozoite, BdP50 was readily detected in purified, *Bd*-derived EVs released by infected RBCs, as revealed by the proteomic analysis conducted in this study. Both the preprocessed (p48) and processed (p35) forms of BdP50 were identified in the EVs by Western blot. Immunogold TEM further confirmed the presence of BdP50 on the surface of *Bd*-derived EVs. The fixation protocol used preserves membrane integrity without causing permeabilization and ensures that antibody binds only occur to epitopes exposed on the external surface of the vesicle membrane. All these findings together suggest that the p35 form could be the predominant end product attached to the outer EV surface, mimicking the active form found on the merozoite surface and the merozoite’s surface architecture.

Notably, BdP50 was not detected as a free, soluble protein in the extracellular medium. Upon treatment with PI-PLC, BdP50 remained associated with the membrane in both free merozoites and *Bd*-derived EVs. This resistance to PI-PLC cleavage may result from the presence of an acyl-chain at the 2-position of inositol within the GPI anchor—a modification known to confer PI-PLC resistance in yeast and in specific mammalian cells, such as human and mouse RBCs, where the acyl-chain is retained [59,60]. A similar mechanism may underline the persistent membrane association observed for BdP50. In support of this hypothesis, acylated inositol has recently been described in *B. divergens* GPIs [23]. Nevertheless, specific biochemical analyses will be required to confirm this in future studies.

The observation of BdP50 in multiple locations allows it to be classified as a multilocated protein, a feature shared by other apicomplexan parasite proteins. These proteins often show a diverse localization pattern in cell apical compartments, the merozoite surface and the extracellular environment [12,14,58,61]. This distribution profile also highlights the functional versatility of BdP50, which may allow it to mediate critical interactions on the surface of both merozoites and EVs within the extracellular milieu.

Like EVs from other eukaryotic cells [62], *Bd*-derived EVs vary in size, and are rich in microRNAs [48]. As our proteomic analysis showed, *Bd*-derived EVs also possess a remarkable capacity to accommodate hundreds of proteins, highlighting proteins related to the invasion process [9,13–15]. It is very likely that these proteins are selectively distributed in subpopulations of EVs to induce important effects on recipient RBC [63]. According to our in vitro invasion assays, one of these critical effects was the interaction of *Bd*-derived EVs with naïve RBCs to promote parasite propagation, which reinforces the relevance of the EV protein content and its potential role in this process.

For this and other effects to occur, efficient and specific recognition between *Bd*-derived EVs and recipient RBCs is required. Such recognition is usually mediated by surface proteins on eukaryotic EVs [63,64]. Since its ability to bind RBCs in binding assays, BdP50 is likely one of the *B. divergens* surface proteins through which free merozoites and *Bd-*derived EVs interact with the human RBC. The structure of BdP50 appears to be suitable for establishing these interactions with the RBC, as it shares homologous α-helical motifs and conserves amino acid residues with other *Babesia* surface proteins. These proteins are Bd37, Bc28.1, BbovMSA1 and BbigGP45, which also interact with the RBC of various vertebrates through different mechanisms [17,20,45,46].

The anti-rBdP50 polyclonal antibodies, which recognized BdP50 on the surface of both free merozoite and *Bd*-derived EVs, inhibited de novo merozoite invasion process by ~88%, confirming the role of BdP50 in host cell interactions and invasion. However, while antibodies significantly reduced parasite growth, viable intraerythrocytic parasites persisted in the in vitro culture system. In fact, the antibody inhibitory effect did not remain constant over time but began to decline after 12 hours and over the following hours post-invasion, despite the continued presence of the antibodies in the extracellular medium. This led to a slight but progressive increase in the number of *B. divergens* iRBCs, suggesting that the initially hostile extracellular environment, characterized by the presence of BdP50 antibodies and the absence of *Bd*-derived EVs, gradually became more favorable to the parasite after the first round of invasion.

Thus, in a hypothetical model, once inside the host cell, intraerythrocytic parasites initiate their activity, including the secretion of *Bd*-derived EVs into the extracellular medium. These EVs prepare naïve red blood cells (RBCs) for invasion, making the subsequent rounds of invasion more efficient. Furthermore, *Bd*-derived EVs that display BdP50 on their surface effectively mimic the merozoite membrane, which also exhibits this protein. Consequently, the continuous and abundant secretion of these EVs could act as a decoy, diverting anti-BdP50 antibodies away from the merozoites and counteracting their inhibitory effect. Ultimately, this mechanism could favor the invasion of the merozoites and thereby promote the propagation of the *B. divergens* iRBC population in vitro. It is also noteworthy that the human cargo of EVs secreted by iRBCs differed from that of EVs secreted by naïve RBCs, suggesting that the biological activity of *B. divergens* parasites after infection certainly alters the human RBC and modifies the extracellular environment.

Previous studies have investigated proteins, such as Bd37, the most abundant protein in *Bd*-derived EVs in this study, highlighting their potential role in immune modulation and their influence in host-pathogen interactions [13,17,19]. For instance, the soluble form of Bd37 present in the extracellular milieu could protect merozoites from the host immune response by acting as a decoy, diverting immune detection away from the merozoite-bound Bd37 [13,19].

The well-orchestrated mechanism involving free merozoites, *Bd*-derived EVs and multilocated proteins could certainly represent a strategy to ensure parasite survival in vitro. Similar adaptive strategies in *B. divergens* might also occur in natural infections to sustain the lifecycle even in hostile host’s environments. Notably, an extracellular environment enriched with *Bd*-derived EVs containing surface proteins such as BdP50 could have significant implications for parasite infection, particularly in immunosuppressed patients with limited antibody production.

Eukaryotic EVs are gaining increasing attention due to their role in intercellular communication and effect on disease outcome. In this context, *Bd*-derived EVs and their cargo, including BdP50, play a critical role in parasite growth and could serve as potential targets for multiple interventions aimed at controlling babesiosis.

## Supporting information

S1 Appendix Supplementary Methods followed by a Reference List. (DOCX)

S1 Fig Anti-*B. divergens* sera recognize *B. divergens* proteins. (A) The immunoprecipitation assay shows anti-*B. divergens* supernatant sera recognizing parasite-specific proteins in the *B. divergens* supernatant (lane 2) and *B. divergens* iRBCs (lane 3) of [35^S^]-labelled parasite cultures. Lane 1 is a negative control using the preimmune rabbit serum. Positions of the molecular mass standards are shown on the left. (B) Immunofluorescent assays were performed using *B. divergens* cultures and anti-*B. divergens* supernatant sera. Bound antibody was detected by using fluorescein isothiocyanate-conjugated anti–rabbit IgG antibodies. *B. divergens* proteins were mostly localized in free merozoites and some intraerythrocytic parasites. Panel 1: *B. divergens* iRBCs and free merozoites captured by differential interference contrast (DIC) image. Panel 2: parasite nucleus stained with DAPI. Panel 3: Fluorescing parasites probed with anti-*B. divergens* supernatant sera. Panel 4: All images overlap. (C) Analysis by SDS-PAGE (upper panels) of the expression of the recombinant proteins: rBdP50Nt, rBdP50Ct and rBdP50 (line 1) and the purification of rBdP50Nt, rBdP50Ct, GST and rBdP50 (line 2). Polyclonal rabbit sera against rBdP50Nt, rBdP50Ct and rBdP50 were tested by Western blot (lower panels) using the corresponding purified recombinant proteins including GST as targets. Molecular mass markers are shown on the left.
(TIF)

S2 Fig Characterization of the *bdp50* gene and primary structure of BdP50. The cartoon above shows standard features of the *bdp50* gene. Non-coding regions of the gene are shown in black and a coding region in a white box. The *bdp50* gene contains the complete ORF (1347 bp) including the initial ATG codon at 5’ -end and the poly (A) tail at the 3’ -end. The BdP50 preproprotein is represented in a white bar that includes an estimated theoretical molecular mass. The N-terminal (Nt) signal peptide in the BdP50Nt and the canonical omega (ω) site to which the glycosylphosphatidylinositol (GPI) anchor is attached in the BdP50C-terminal (Ct) are represented in grey. The cartoon also shows post-translational modifications sites, such as proteolytic cleavage of the Nt and Ct motifs and the transfer of a GPI-anchor to generate a nascent GPI-AP and finally a mature GPI-AP which is represented in a white bar that includes an estimated theoretical molecular mass. The cartoon below shows the rBdP50Nt and rBdP50Ct-GST fusion proteins, which exclude Nt signal peptide and C-terminal signal for GPI attachment, respectively. There is also the His-tagged fusion rBdP50 protein, which lacks Nt and Ct signals. The core regions of the recombinant proteins, including the position of the Nt and Ct amino acids, are represented as grey boxes.
(TIF)

S3 Fig Protein structure analysis of BdP50. (A) The table shows the similarity based on the root mean square deviation (RMSD) score of pruned atom pairs and all atom pairs from structural superimpositions of BdP50 with related *Babesia* spp. proteins. RMSD <2 Å indicates significant structural similarity. (B) Superimposition of BdP50 (golden) with the 1^st^ model of nuclear magnetic resonance (NMR) structure of delta-Bd37 of *B. divergens* (cyan). The helix of BdP50 (purple) overlaps with a delta-Bd37 helix (cyan). The RMSD between the particular 142–164 residues of BdP50 and 197–219 residues of delta-Bd37 model was 4.321 Å.(C): Superimposition of BdP50 (golden) with the 13^th^ NMR structure of *B. canis* 28.1 (Bc28.1). Helices of BdP50 (magenta and orange) overlap with two Bc28.1 helices (pink). One of the helices shows a RMSD of 0.942 Å between 182–190 BdP50 residues and 148–156 Bc28.1 residues. The RMSD between 138–164 BdP50 residues and 104–130 Bc28.1 residues of the other helix was 1.219 Å. (D): Superimposition of BdP50 (golden) with the AlphaFold2 predicted BbovMSA1 structure of *B. bovis* (cyan). The purple helix of BdP50 overlaps with a helix of BbovMSA1. The RMSD between 380–391 BdP50 residues and 177–188 BbovMSA1 residues was 1.285 Å. (E) Superimposition of BdP50 (golden) with the AlphaFold2 predicted BbigGP45 structure of *B. bigemina* (pink). The yellow helix of BdP50 overlaps with a similar BbigGP45 helix. The RMSD between 380–391 BdP50 residues and 177–188 BbigGP45 residues was 1.980 Å. Nt: N-terminal, Ct: C-terminal. (F) Predicted align error (PAE) plots of the AlphaFold models and the confidence score coloring. (G) Sequence alignments reveal the hydrophobic nature of helices in BdP50 compared to the other *Babesia* spp proteins. Alignment between BdP50 and delta-Bd37 highlights one conserved serine, two lysines, and four leucines; similarly, comparison with Bc28.1 shows conserved leucine, tryptophan, and isoleucine in one helix and tryptophan and phenylalanine residues in the other one, emphasizing helix hydrophobicity. Alignment with BbovMSA1 identifies conserved valine and leucine residues, further supporting the hydrophobicity of the helices. Additionally, alignment with BbigGP45 reveals conserved leucine and glutamine, alongside other hydrophobic residues. Alignments were performed using UniProt’s alignment tool (https://www.uniprot.org/align), which uses the Clustal Omega method.
(TIF)

S4 Fig BdP50 is localized to the rhoptries and the surface of *B. divergens* parasites. Thawed cryosections of non-infected RBCs (A, B), *B. divergens* infected RBCs (C, D) and free extracellular merozoites (E, F) were labelled with anti-rBdP50Ct antibodies followed by a goat anti-rabbit secondary antibody coupled to 10 nm gold. (A) Overview of uRBCs. (B) Detail from (A). The arrow points to the background label in the cytosol. (C) Overview of the parasite inside the RBC. (D) Detail from (C). BdP50 is mainly present at the plasma membrane of the parasite (arrows). In addition, it is present on the rhoptries (arrowheads). (E) Overview of a free merozoite. (D) Detail from (E). BdP50 localizes mainly to the plasma membrane (arrows). N – nucleus. Scale bar: (A) – 1 µm, (C, E) – 500 nm, (B, D, F) – 200nm.
(TIF)

S5 Fig Proteomic analysis of *Bd*-derived EVs. Total proteins from *Bd*-derived EVs were, trypsinized and analysed by tandem mass spectrometry. (A) A graphic shows difference in abundance of *B. divergens* and human proteins identified in *Bd*-derived EVs and uRBC-derived EVs (B) Venn diagram depicting differences and similarities between the *Bd*-derived EVs replicates used in this study. (C) Pathway analysis of *Bd*-derived EV replicates shows proteins related to biological processes, molecular functions and cellular components. Proteins are associated with their number and plotted as bar graphs. Reference list: *Homo sapiens* proteome from Uniprot and *B. divergens* proteome [9].
(TIF)

S6 Fig List of human proteins related to biological processes detected in *Bd*-derived EV and uRBC-derived EV cargos. The horizontal bar graph shows the human proteins. Reference list: *Homo sapiens* proteome from Uniprot.
(TIF)

S7 Fig List of human proteins related to molecular functions and cellular components detected in *Bd*-derived EV and uRBC-derived EV cargos. The horizontal bar graph shows the human proteins. Reference list: *Homo sapiens* proteome from Uniprot.
(TIF)

S8 Fig Most abundant *B. divergens* proteins in *Bd*-derived extracellular vesicles. The table show the 16 most abundant parasite proteins found in *Bd*-derived EVs per replicate. (*) *B. divergens* proteins involved in the red blood cell invasion process. (^+^) Molecules that were also identified by the immunoscreening of the *B. divergens* cDNA library. NUP: number unique peptides, TUS: Total unique spectra.
(TIF)

S1 Table *Babesia divergens* cDNAs identified in the parasite expression library. (DOCX)

S2 Table Quantification of labeling density. The table shows the associated gold particles per surface area (GP/µm2) counted in uninfected RBCs (uRBCs) compared to the GP/µm2 counted in infected RBCs (iRBCs).
(DOCX)

S3 Table Human proteins identified in *Bd*-derived EVs. Reference list: *Homo sapiens* proteome from Uniprot.
(DOCX)

S4 Table Human proteins identified in uRBCs-derived EVs replicates. Reference list: *Homo sapiens* proteome from Uniprot.
(DOCX)

S5 Table *Babesia divergens* proteins identified in *Bd*-derived EVs: replicate 1 (text in black), replicate 2 (text in red) and replicate (text in blue). NUP: number unique peptides, TUS: Total unique spectra. Reference list: *B. divergens* proteome [9].
(DOCX)
