# Supplementary material for: Elucidating the functional role of the novel BdP50 protein and extracellular vesicles in the human erythrocyte infection by Babesia divergens
Source: PLoS Negl Trop Dis. 2025 Aug 13;19(8):e0013401. doi: 10.1371/journal.pntd.0013401 (PMC12370190; doi:10.1371/journal.pntd.0013401)
Supplement: S1 Appendix — (DOCX) [file pntd.0013401.s001.docx]

**Supplementary Methods**

***B. divergens* supernatant antisera production**

*Babesia divergens* cultures were consecutive centrifuged at 400 x g, and 2,000 x g for 5 minutes at 4°C to collect the parasite supernatant. A protease inhibitor mixture (Sigma Aldrich) was added to the supernatant and centrifuged twice at 8,000 x g for 5 minutes at 4°C to remove cell debris. Then, the *B. divergens* supernatant was concentrated using Amicon Ultra-4 10K filter units (Millipore, Belford, MA) at 4,000 x g for 15 min at 4°C. Protein concentration of the *B. divergens* supernatant was determined using the Pierce BCA protein assay kit (Thermo Fisher Scientific). Supernatants from uRBCs cultures (uninfected supernatants) were obtained following the same protocol.

For polyclonal anti*-B. divergens* supernatant sera production, two female New Zealand White rabbits were injected subcutaneously on days 0, 21 and 49 with 500 μg of parasite *B. divergens* supernatant proteins. On the 0 day, before the first immunization, 20 ml of serum were obtained from each rabbit and used as a negative control. The first injection was subcutaneous and contained 500 μl of complete Freund´s adjuvant (Sigma Aldrich). The following injections were intramuscular and contained 500 μl of incomplete Freund´s adjuvant (Sigma Aldrich). Fifteen days after the third immunization, the animals were bled and sera were collected, preadsorbed against uninfected supernatants and title by indirect immunofluorescent assay (IFA).

**Protein expression and purification and anti-BdP50 sera production**

DNAs encoding Glu^21^–Leu^225^ (BdP50Nt), Lys^236^-Ser^418^ (BdP50Ct) and Glu^21^-Ser^418^ (BdP50) were amplified by PCR from the BdP50 cDNA. Bd50Nt and BdP50Ct cDNAs were cloned into the expression plasmid vector pGEX-4T1 (GE Healthcare, Waukesha, WI) and BdP50 cDNA was cloned into the expression plasmid pET-30a (Qiagen, Valencia, CA) according to the manufacturer's instructions. *Escherichia coli* strain BL21-gold (DE3) plysS (Agilent Technologies, Santa Clara, CA, USA) was transformed with BdP50Nt and BdP50Ct expression plasmids. *E. coli* M13 (Qiagen) was transformed with BdP50 expression plasmid. Then, super optimal broth (SOB) medium (250 ml) containing 100 μg ml^-1^ ampicillin (Sigma Aldrich) was inoculated with 1 ml of fresh overnight transformed *E. coli* strain BL21-gold and *E. coli* M13 cultures and grown at 37 °C to A600 = 0.6, before induction with 0.5 mM isopropyl-β-d-thiogalactopyranoside (IPTG) (Sigma Aldrich). After 3 h of induction to produce rBdP50Nt, rBdP50Ct and rBdP50 recombinant proteins, cells were pelleted and resuspended in B-PER bacterial protein extraction reagent (Thermo Fisher Scientific) supplemented with a protease inhibitor mixture (Sigma Aldrich) for 10 min. Then, the insoluble material was removed by centrifugation and the soluble fraction was used to purify rBdP50Nt, rBdP50Ct and rBdP50 by using glutathione-Sepharose 4B (GE Healthcare) and HisPur Ni-NTA resin (Thermo Fisher Scientific), respectively as described by the manufacturer. Protein concentration was determined by Pierce BCA protein assay kit (Thermo Fisher Scientific). The purified rBdP50Nt, rBdP50Ct and rBdP50 products were analyzed by Western blot using anti-Glutathione-S-Transferase (GST) polyclonal (Sigma Aldrich) or anti-Histidine (His) Tag monoclonal (Qiagen) antibodies, respectively, and used to immunize rabbits. Polyclonal sera were raised against rBdP50Nt, rBdP50Ct and rBdP50, respectively, using the standard immunization protocol for rabbits described above. Anti-rBdP50Nt, -rBdP50Ct and -rBdP50 antibodies were tested by IFA using *B. divergens* cultures following the protocol above, and by Western blot using the recombinant proteins as targets and the rabbit sera diluted from 1:100 to 1:5,000. Antigen detection was assessed by a colorimetric reaction (CN-DAB Substrate kit, Thermo Fihser Scientific)

**Immunoprecipitation**

Freshly cultured parasites were washed and resuspended in methionine-free medium (RPMI 1640, MP Biomedicals, Inc, Aurora, OH). 200 _Ci ml^-1^ of [^35^S] methionine/cysteine (PerkinElmer Life Sciences, Boston, MA) was added, and parasites were incubated at 37 °C for 2 h. Then, parasites were detergent-solubilized and treated with preinmune rabbit sera and anti-*B. divergens* supernatant sera and analyzed by inmmunoprecipitation following previous protocols [1,2].

**Immunoscreening of a *B. divergens* cDNA expression library**

A *B. divergens* cDNA expression library [1] was screened using anti-*B. divergens* supernatant sera following standard protocols [1]. Positive clones were purified and amplified by PCR using T3 and T7 universal primers [1]. The cDNA products were sequenced using an ABI PRISM 3730XL DNA Analyzer (Applied Biosystems, San Francisco, CA, USA).

**Depletion of extracellular vesicles from human sera and *B. divergens* culture supernatants**

The human serum used to grow *B. divergens* parasites was depleted to minimize the presence of human EVs. To this aim, human serum was diluted (1:1) with RPMI and centrifuged for 22 h at 118,388 g using a SW40Ti rotor (Beckman Coulter, Indianapolis, IN). Only the upper, light-coloured supernatant was carefully transferred to a new tube and considering as a low human vesicle (LHV) serum, while the pellet enriched in EVs was discharged. The complete medium containing the LHV serum (LHV-complete medium) was used in *B. divergens* cultures resulting optimal for the propagation of the parasites in vitro. When *B. divergens* cultures, using the LHV serum, reached 25-35% parasitemia, culture supernatants were collected and centrifuged sequentially at 500 x g, 2,000 x g and 3,500 x g for 15 min each at 4°C. Then, EVs were also removed from the supernatants following the same procedure. Upper parasite supernatants were carefully collected and concentrated using a Vivaspin 10 K filter (Sartorius, Gloucestershire, UK) at 6,000 x g and 4°C until used. Protein concentration of the supernatants was determined by a BCA assay (Thermo Fisher Scientific). The resulting samples were used for checking the presence of a soluble form of BdP50 by Western blot.

**In-gel digestion**

Purified EVs (5 μg) were suspended in a volume up to 50 μl of sample buffer and then applied onto 1.2-cm wide wells of a conventional SDS-PAGE gel (1.5 mm-thick, 4% stacking, and 10% resolving). The run was stopped as soon as the front entered 3 mm into the resolving gel, therefore the whole proteome became concentrated in the stacking/resolving gel interface. The unseparated protein bands were visualized by Coomassie staining, excised, cut into cubes (2 x 2 mm), and placed in 0.5 ml microcentrifuge tubes. The gel pieces were destained in acetonitrile: water (ACN:H_2_O, 1:1), reduced and alkylated (disulfide bonds from cysteinyl residues were reduced with 10 mM DTT for 1 h at 56 ºC and then thiol groups were alkylated with 10 mM iodoacetamide for 30 min, at RT, in darkness), and digested *in situ* with sequencing grade trypsin (Promega, Madison, WI) following a previous protocol [5] with minor modifications. The gel pieces were shrunk by removing all liquid using sufficient ACN. Acetonitrile was pipetted out and the gel pieces were dried in a speed vacuum. The dried gel pieces were re-swollen in 100 mM Tris-HCl pH 8, 10mM CaCl_2_ with 60 ng/μl trypsin at 5:1 protein:enzyme (w/w) ratio*.* The tubes were kept on ice for 2 h and incubated at 37°C for 12 h. Digestion was stopped by the addition of 1% TFA. Whole supernatants were dried down and then desalted onto ZipTip C18 Pipette tips (Millipore) until the mass spectrometric analysis.

**Reverse phase-liquid chromatography RP-LC-MS/MS analysis**

The desalted protein digest was dried, resuspended in 10 μl of 0.1% formic acid and analyzed by RP-LC-MS/MS in an Easy-nLC 1200 system coupled to an ion trap LTQ-Orbitrap-Velos-Pro hybrid mass spectrometer (Thermo Fisher Scientific). The peptides were concentrated (on-line) by reverse phase chromatography using a 0.1mm × 20 mm C18 RP precolumn (Thermo Fisher Scientific) and then separated using a 0.075mm x 250 mm C18 RP column (Phenomenex, Torrance, CA) operating at 0.25 μl/min. Peptides were eluted using a 90 min dual gradient. The gradient profile was set as follows: 5−25% solvent B for 67 min, 25−40% solvent B for 23 min, 40−100% solvent B for 2min and 100% solvent B for 18 min (Solvent A: 0.1% formic acid in water, solvent B: 0.1% formic acid, 80% acetonitrile in water). ESI ionization was done using a Nano-bore emitters Stainless Steel ID 30 μm (Proxeon, Odense, DK) interface at 2.1 kV spray voltage with S-Lens of 60%. The Orbitrap resolution was set at 30,000 [6]. Peptides were detected in survey scans from 400 to 1600 amu (1 μscan), followed by twenty data dependent MS/MS scans (Top 20), using an isolation width of 2u (in mass-to-charge ratio units), normalized collision energy of 35%, and dynamic exclusion applied for 60-second periods. Charge-state screening was enabled to reject unassigned and singly charged protonated ions.

**Release of BdP50 by phosphatidylinositol-specific phospholipase C**

Pellets of purified free merozoites and *Bd*-derived extracellular vesicles (*Bd*-derived EVs) were resuspended in 50 µL of RPMI 1640 medium (Life Technologies Corporation, Carlsbad, CA, USA). Samples were incubated with phosphatidylinositol-specific phospholipase C (PI-PLC) (2 U/mL; Thermo Fisher Scientific, Waltham, MA,) or a glycerol control for different lengths of time from 15 min to 2 h at 37 °C [7]. After incubation, the pellets and the supernatant from the merozoite samples were collected by centrifugation at 10,000× g and 4 °C for 30 min. The pellets and the supernatant from the *Bd*-derived EVs were collected by centrifugation at 20,000× g and at 4 °C for 90 min. All samples were analysed by Western blot using rabbit polyclonal anti-rBdP50 serum (see Materials and Methods section).

**References**

1. Montero E, Gonzalez LM, Rodriguez M, Oksov Y, Blackman MJ, Lobo CA. A Conserved Subtilisin Protease Identified in *Babesia divergens* Merozoites. J Biol Chem. 2006;281: 35717–35726. doi:10.1074/jbc.M604344200

2. Montero E, Rodriguez M, Oksov Y, Lobo CA. *Babesia divergens* Apical Membrane Antigen 1 and Its Interaction with the Human Red Blood Cell. Infect Immun. 2009;77: 4783–4793. doi:10.1128/IAI.00969-08

3. Madeira F, Pearce M, Tivey ARN, Basutkar P, Lee J, Edbali O, et al. Search and sequence analysis tools services from EMBL-EBI in 2022. Nucleic Acids Res. 2022;50: W276–W279. doi:10.1093/nar/gkac240

4. Moreno M-L, Escobar J, Izquierdo-Álvarez A, Gil A, Pérez S, Pereda J, et al. Disulfide stress: a novel type of oxidative stress in acute pancreatitis. Free Radic Biol Med. 2014;70: 265–277.

5. Shevchenko A, Wilm M, Vorm O, Mann M. Mass Spectrometric Sequencing of Proteins from Silver-Stained Polyacrylamide Gels. Anal Chem. 1996;68: 850–858. doi:10.1021/ac950914h

6. Alonso R, Pisa D, Marina AI, Morato E, Rábano A, Rodal I, et al. Evidence for Fungal Infection in Cerebrospinal Fluid and Brain Tissue from Patients with Amyotrophic Lateral Sclerosis. Int J Biol Sci. 2015;11: 546–558. doi:10.7150/ijbs.11084

7. Fujita M, Maeda Y, Kinoshita T. Phosphatidylinositol-specific phospholipase C (PI-PLC) treatment of glycosylphosphatidylinositol (GPI)-anchored proteins on the cell surface of intact cells. In: Nishihara S, Angata K, Aoki-Kinoshita KF, Hirabayashi J, editors. Glycoscience Protocols (GlycoPODv2). Saitama (JP): Japan Consortium for Glycobiology and Glycotechnology; 2021. Available: http://www.ncbi.nlm.nih.gov/books/NBK593927/
