# Supplementary figures and images for: Elucidating the functional role of the novel BdP50 protein and extracellular vesicles in the human erythrocyte infection by Babesia divergens
Source: PLoS Negl Trop Dis. 2025 Aug 13;19(8):e0013401. doi: 10.1371/journal.pntd.0013401 (PMC12370190; doi:10.1371/journal.pntd.0013401)

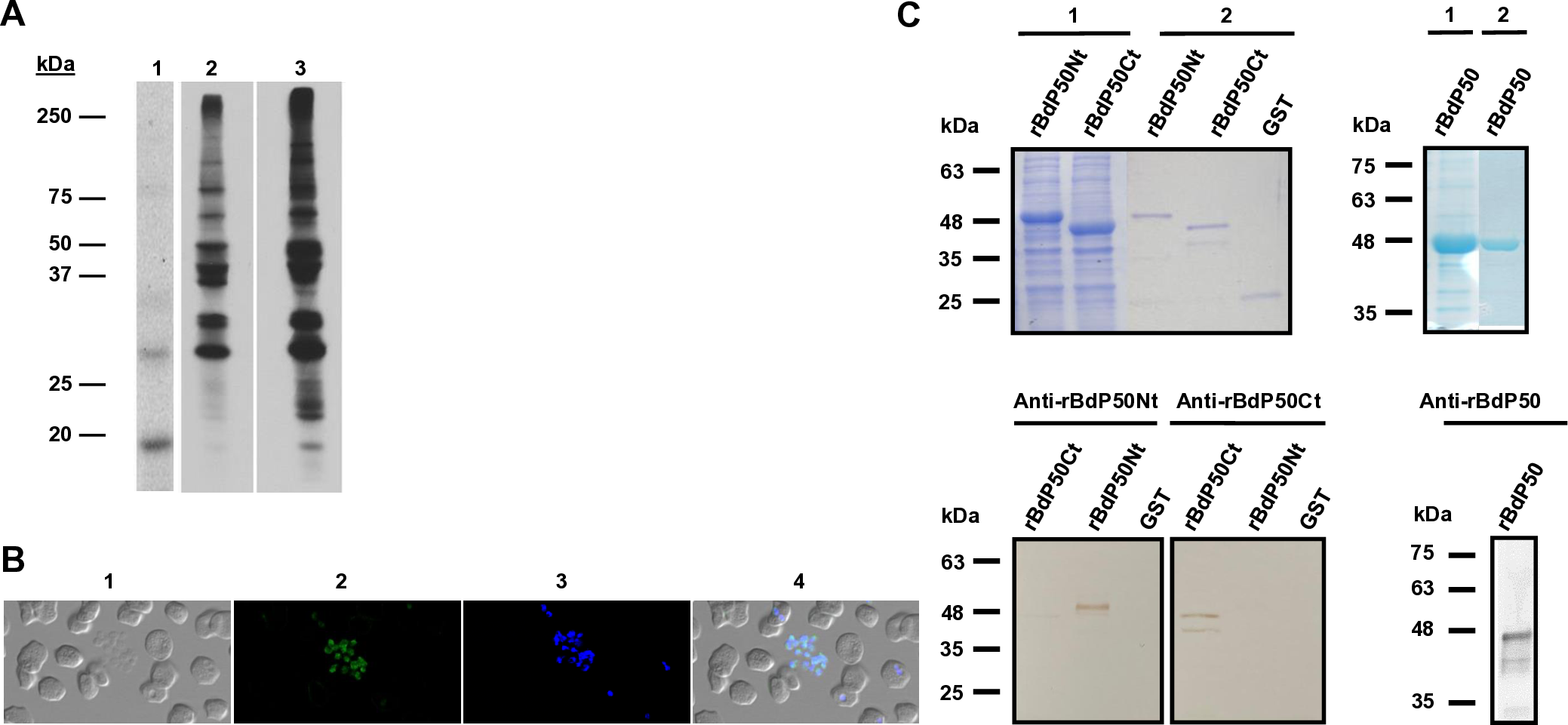

Supplement: S1 Fig — (A) The immunoprecipitation assay shows anti-B. divergens supernatant sera recognizing parasite-specific proteins in the B. divergens supernatant (lane 2) and B. divergens iRBCs (lane 3) of [35S]-labelled parasite cultures. Lane 1 is a negative control using the preimmune rabbit serum. Positions of the molecular mass standards are shown on the left. (B) Immunofluorescent assays were performed using B. divergens cultures and anti-B. divergens supernatant sera. Bound antibody was detected by using fluorescein isothiocyanate-conjugated anti–rabbit IgG antibodies. B. divergens proteins were mostly localized in free merozoites and some intraerythrocytic parasites. Panel 1: B. divergens iRBCs and free merozoites captured by differential interference contrast (DIC) image. Panel 2: parasite nucleus stained with DAPI. Panel 3: Fluorescing parasites probed with anti-B. divergens supernatant sera. Panel 4: All images overlap. (C) Analysis by SDS-PAGE (upper panels) of the expression of the recombinant proteins: rBdP50Nt, rBdP50Ct and rBdP50 (line 1) and the purification of rBdP50Nt, rBdP50Ct, GST and rBdP50 (line 2). Polyclonal rabbit sera against rBdP50Nt, rBdP50Ct and rBdP50 were tested by Western blot (lower panels) using the corresponding purified recombinant proteins including GST as targets. Molecular mass markers are shown on the left. (TIF) [file pntd.0013401.s002.tif]

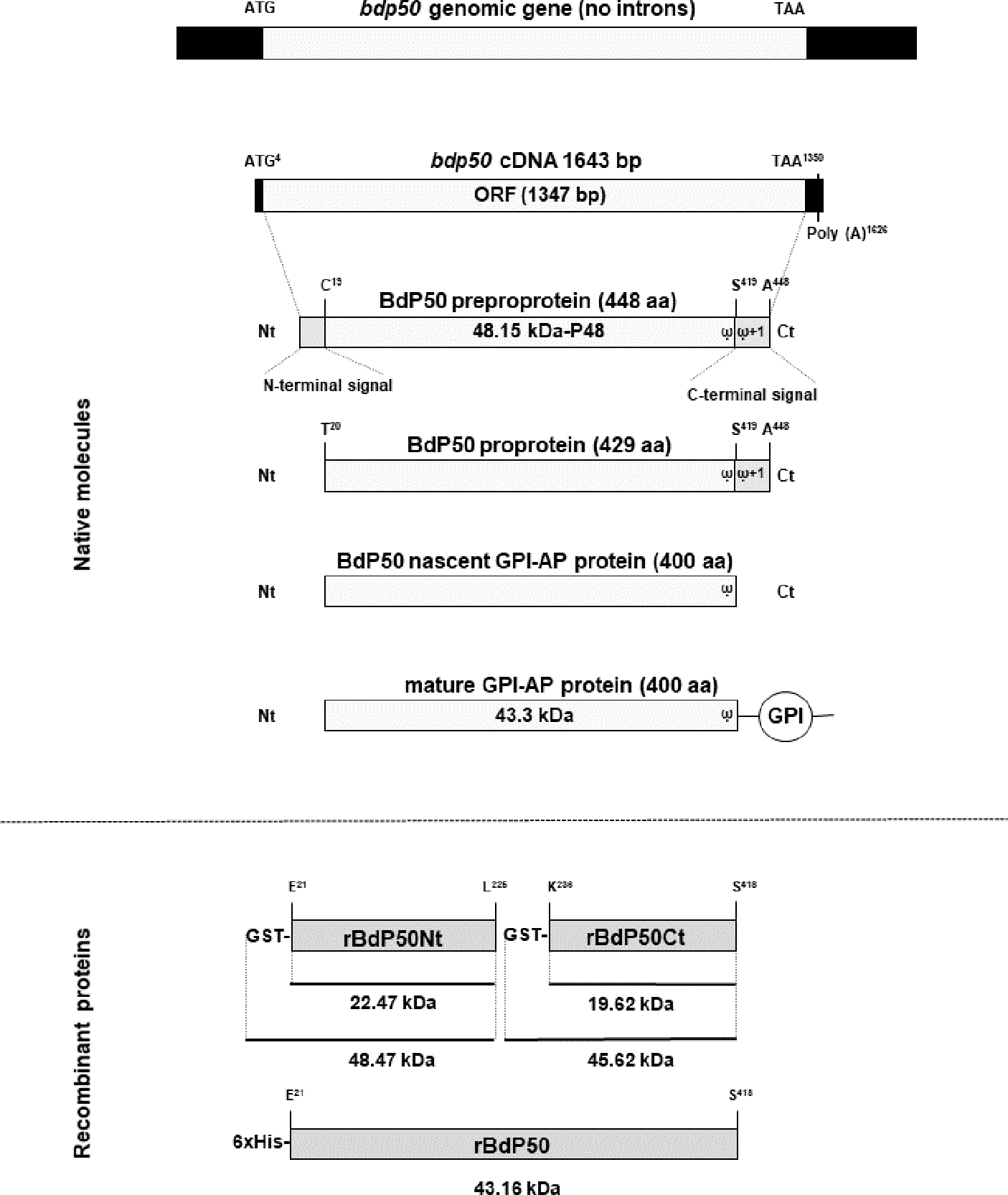

Supplement: S2 Fig — The cartoon above shows standard features of the bdp50 gene. Non-coding regions of the gene are shown in black and a coding region in a white box. The bdp50 gene contains the complete ORF (1347 bp) including the initial ATG codon at 5’ -end and the poly (A) tail at the 3’ -end. The BdP50 preproprotein is represented in a white bar that includes an estimated theoretical molecular mass. The N-terminal (Nt) signal peptide in the BdP50Nt and the canonical omega (ω) site to which the glycosylphosphatidylinositol (GPI) anchor is attached in the BdP50C-terminal (Ct) are represented in grey. The cartoon also shows post-translational modifications sites, such as proteolytic cleavage of the Nt and Ct motifs and the transfer of a GPI-anchor to generate a nascent GPI-AP and finally a mature GPI-AP which is represented in a white bar that includes an estimated theoretical molecular mass. The cartoon below shows the rBdP50Nt and rBdP50Ct-GST fusion proteins, which exclude Nt signal peptide and C-terminal signal for GPI attachment, respectively. There is also the His-tagged fusion rBdP50 protein, which lacks Nt and Ct signals. The core regions of the recombinant proteins, including the position of the Nt and Ct amino acids, are represented as grey boxes. (TIF) [file pntd.0013401.s003.tif]

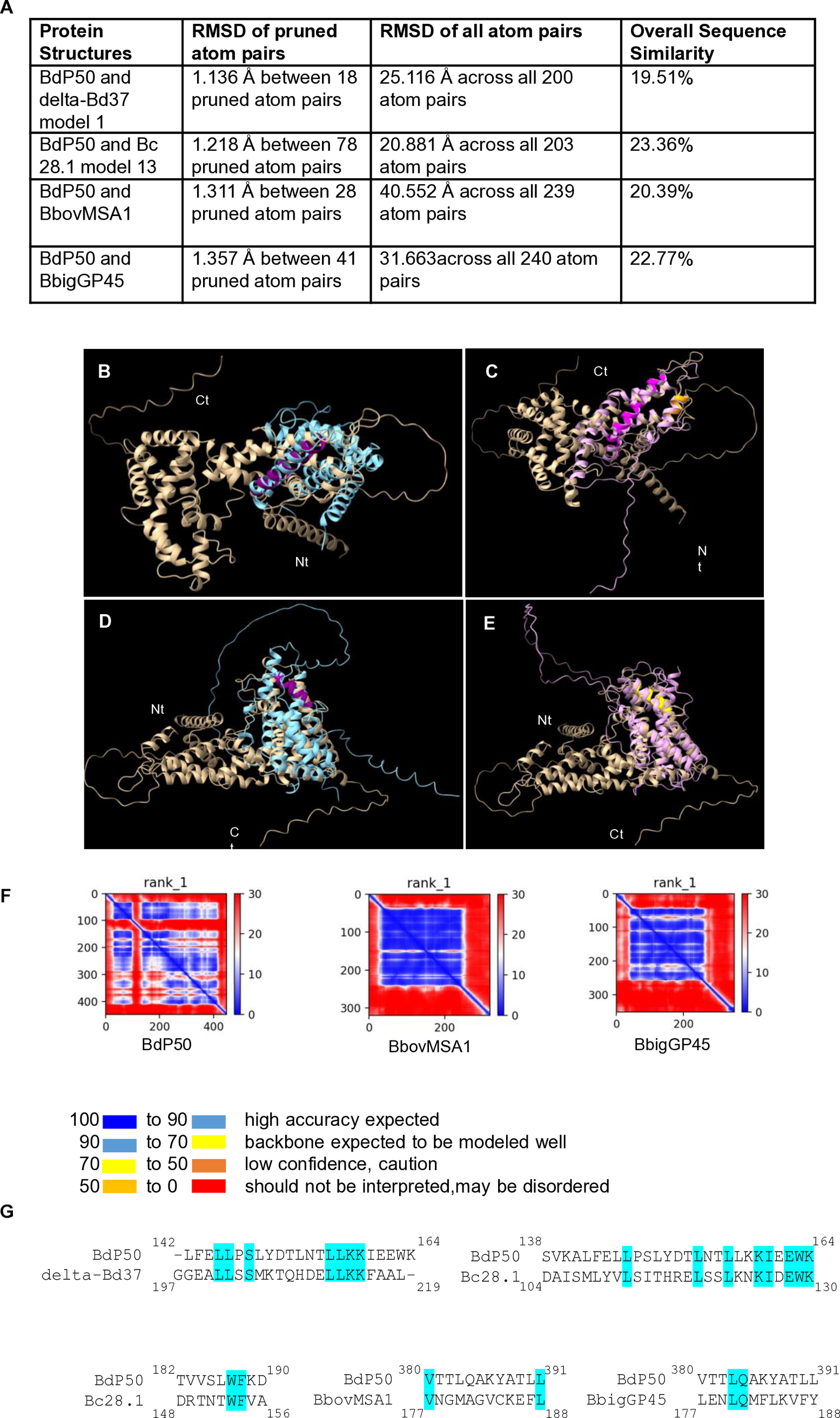

Supplement: S3 Fig — (A) The table shows the similarity based on the root mean square deviation (RMSD) score of pruned atom pairs and all atom pairs from structural superimpositions of BdP50 with related Babesia spp. proteins. RMSD <2 Å indicates significant structural similarity. (B) Superimposition of BdP50 (golden) with the 1st model of nuclear magnetic resonance (NMR) structure of delta-Bd37 of B. divergens (cyan). The helix of BdP50 (purple) overlaps with a delta-Bd37 helix (cyan). The RMSD between the particular 142–164 residues of BdP50 and 197–219 residues of delta-Bd37 model was 4.321 Å.(C): Superimposition of BdP50 (golden) with the 13th NMR structure of B. canis 28.1 (Bc28.1). Helices of BdP50 (magenta and orange) overlap with two Bc28.1 helices (pink). One of the helices shows a RMSD of 0.942 Å between 182–190 BdP50 residues and 148–156 Bc28.1 residues. The RMSD between 138–164 BdP50 residues and 104–130 Bc28.1 residues of the other helix was 1.219 Å. (D): Superimposition of BdP50 (golden) with the AlphaFold2 predicted BbovMSA1 structure of B. bovis (cyan). The purple helix of BdP50 overlaps with a helix of BbovMSA1. The RMSD between 380–391 BdP50 residues and 177–188 BbovMSA1 residues was 1.285 Å. (E) Superimposition of BdP50 (golden) with the AlphaFold2 predicted BbigGP45 structure of B. bigemina (pink). The yellow helix of BdP50 overlaps with a similar BbigGP45 helix. The RMSD between 380–391 BdP50 residues and 177–188 BbigGP45 residues was 1.980 Å. Nt: N-terminal, Ct: C-terminal. (F) Predicted align error (PAE) plots of the AlphaFold models and the confidence score coloring. (G) Sequence alignments reveal the hydrophobic nature of helices in BdP50 compared to the other Babesia spp proteins. Alignment between BdP50 and delta-Bd37 highlights one conserved serine, two lysines, and four leucines; similarly, comparison with Bc28.1 shows conserved leucine, tryptophan, and isoleucine in one helix and tryptophan and phenylalanine residues in the other one, emphasizing helix hydr [file pntd.0013401.s004.tif]

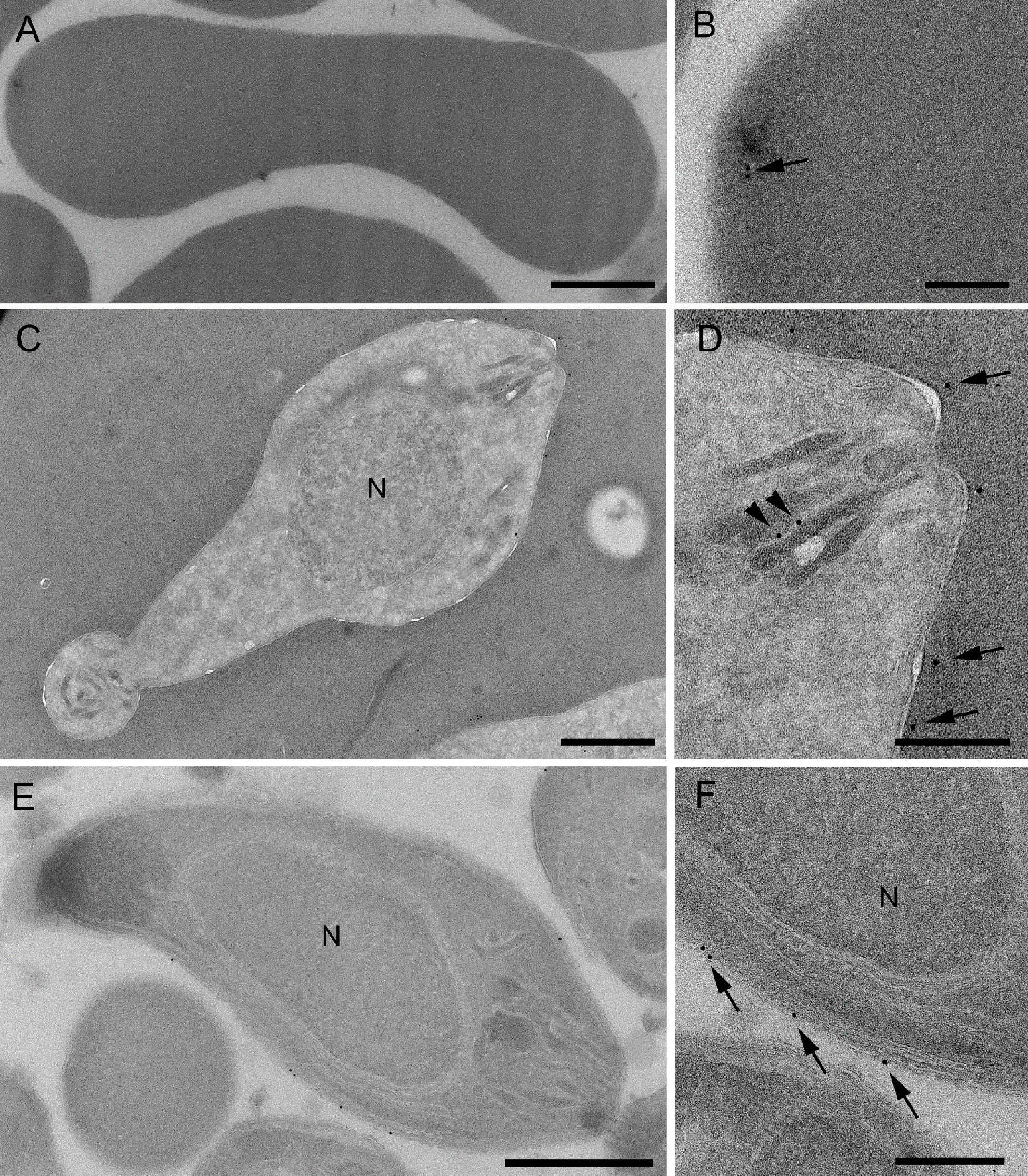

Supplement: S4 Fig — Thawed cryosections of non-infected RBCs (A, B), B. divergens infected RBCs (C, D) and free extracellular merozoites (E, F) were labelled with anti-rBdP50Ct antibodies followed by a goat anti-rabbit secondary antibody coupled to 10 nm gold. (A) Overview of uRBCs. (B) Detail from (A). The arrow points to the background label in the cytosol. (C) Overview of the parasite inside the RBC. (D) Detail from (C). BdP50 is mainly present at the plasma membrane of the parasite (arrows). In addition, it is present on the rhoptries (arrowheads). (E) Overview of a free merozoite. (D) Detail from (E). BdP50 localizes mainly to the plasma membrane (arrows). N – nucleus. Scale bar: (A) – 1 µm, (C, E) – 500 nm, (B, D, F) – 200nm. (TIF) [file pntd.0013401.s005.tif]

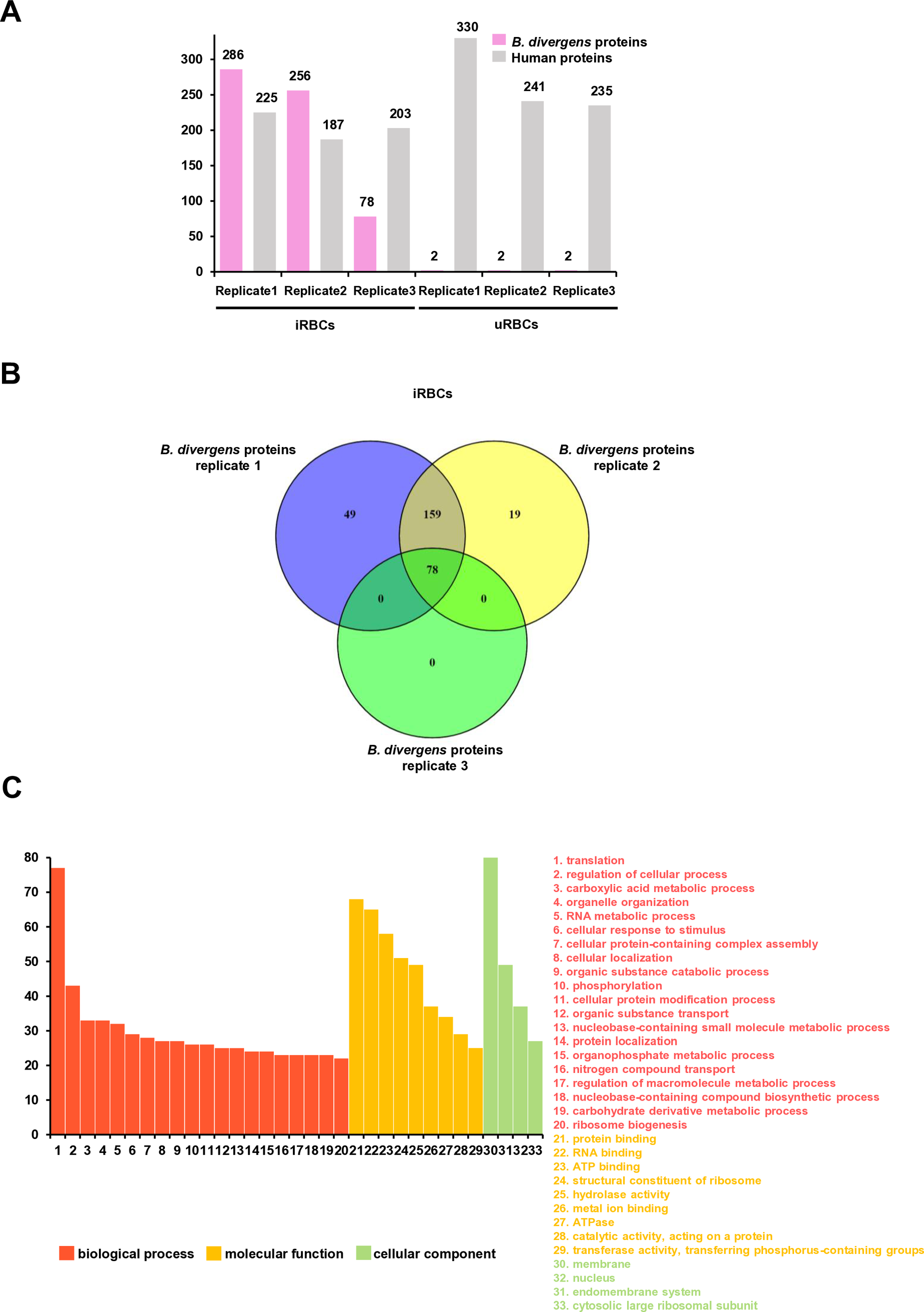

Supplement: S5 Fig — Total proteins from Bd-derived EVs were, trypsinized and analysed by tandem mass spectrometry. (A) A graphic shows difference in abundance of B. divergens and human proteins identified in Bd-derived EVs and uRBC-derived EVs (B) Venn diagram depicting differences and similarities between the Bd-derived EVs replicates used in this study. (C) Pathway analysis of Bd-derived EV replicates shows proteins related to biological processes, molecular functions and cellular components. Proteins are associated with their number and plotted as bar graphs. Reference list: Homo sapiens proteome from Uniprot and B. divergens proteome [9]. (TIF) [file pntd.0013401.s006.tif]

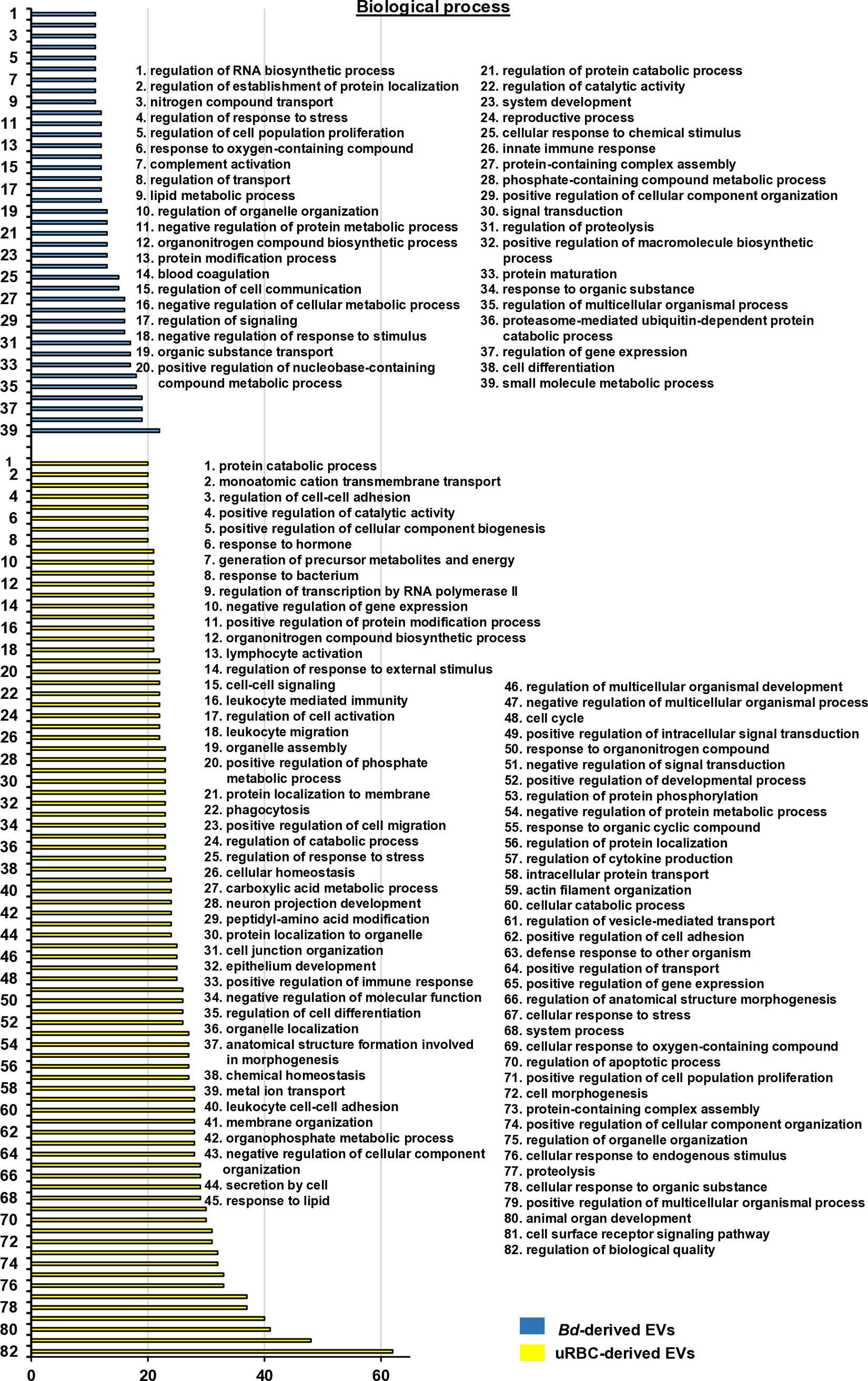

Supplement: S6 Fig — The horizontal bar graph shows the human proteins. Reference list: Homo sapiens proteome from Uniprot. (TIF) [file pntd.0013401.s007.tif]

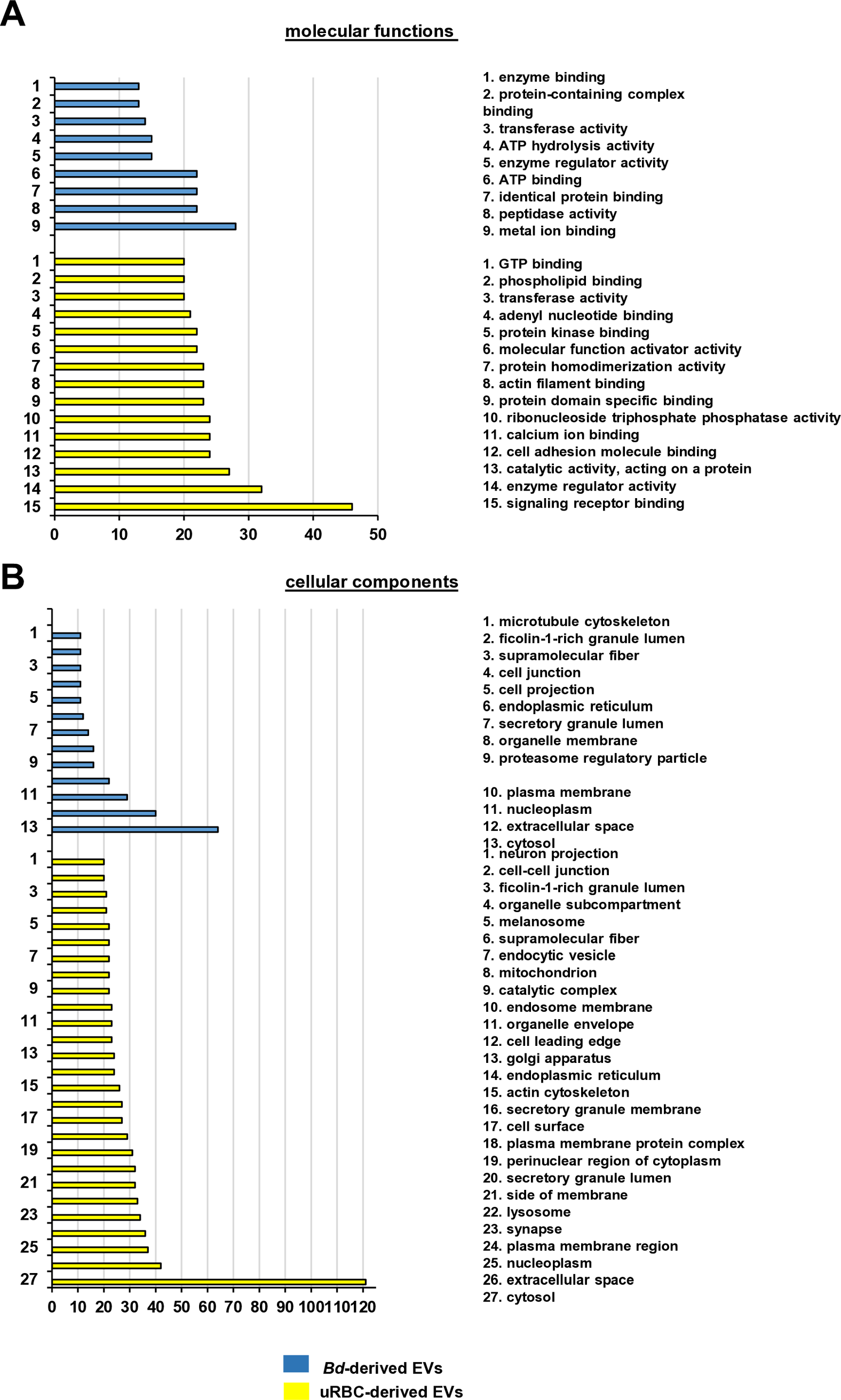

Supplement: S7 Fig — The horizontal bar graph shows the human proteins. Reference list: Homo sapiens proteome from Uniprot. (TIF) [file pntd.0013401.s008.tif]

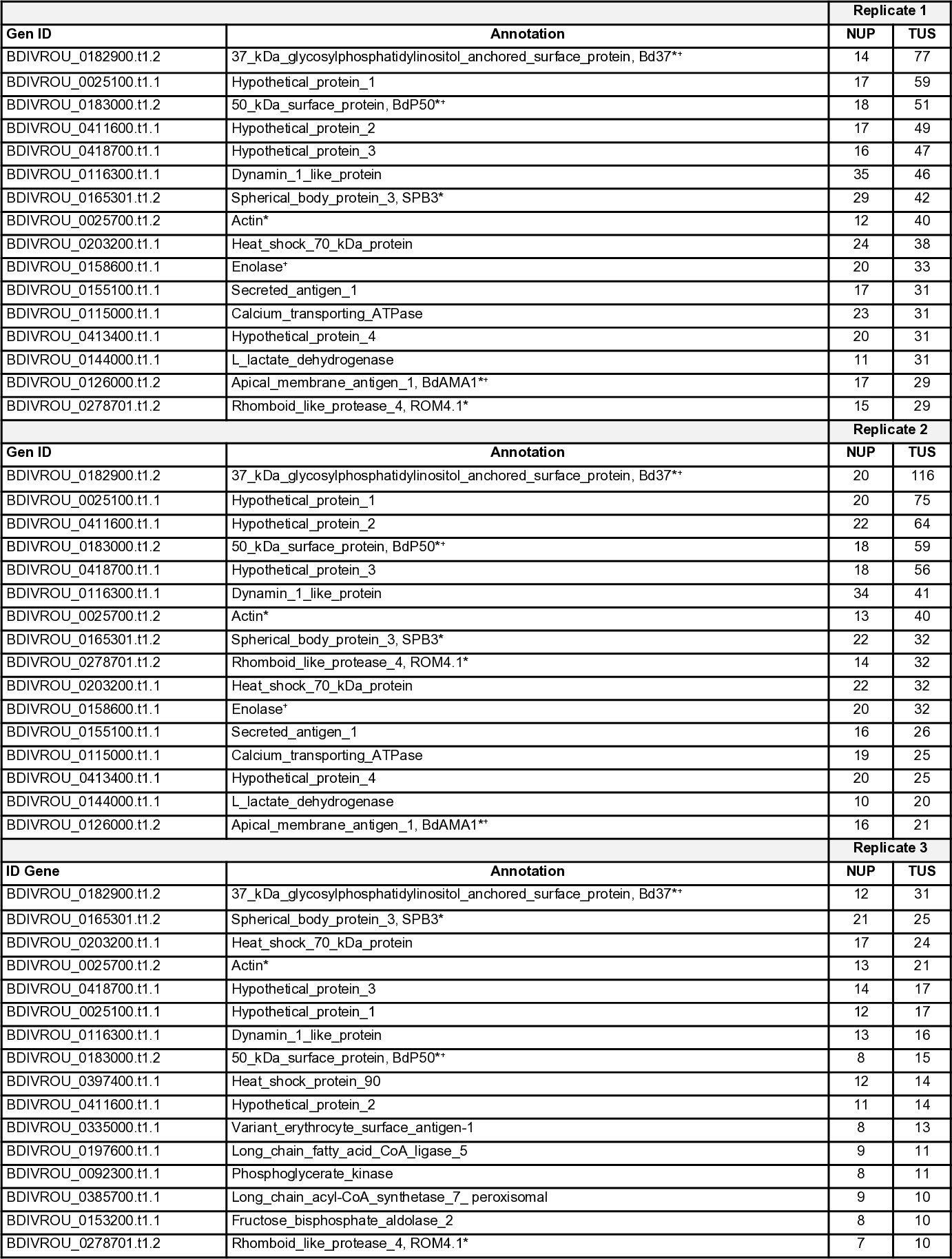

Supplement: S8 Fig — The table show the 16 most abundant parasite proteins found in Bd-derived EVs per replicate. (*) B. divergens proteins involved in the red blood cell invasion process. (+) Molecules that were also identified by the immunoscreening of the B. divergens cDNA library. NUP: number unique peptides, TUS: Total unique spectra. (TIF) [file pntd.0013401.s009.tif]
