# Supplementary material for: Elucidating the functional role of the novel BdP50 protein and extracellular vesicles in the human erythrocyte infection by Babesia divergens
Source: PLoS Negl Trop Dis. 2025 Aug 13;19(8):e0013401. doi: 10.1371/journal.pntd.0013401 (PMC12370190; doi:10.1371/journal.pntd.0013401)
Supplement: S1 Table — (DOCX) [file pntd.0013401.s010.docx]

| **GeneBank accession number** | **cDNA** |
| --- | --- |
| HF969321 | 50-47 kDa merozoite surface protein (BdP50) |
| AJ422214 | glycosylphosphatidylinositol-anchored merozoite surface protein (Bd37) |
| HF969320 | 2-phosphoglycerate dehydratase (enolase) |
| HF969319 | guanosine monophosphate reductase (gmp reductase) |
| HF969318 | 60S ribosomal protein L3 |
| HF969322 | hypothetical protein |
| HF9693234 | 12D3 antigen (12D3) |
| HF969324 | adenosylhomocysteinase |
| MZ836245 | apical membrane antigen 1 (AMA1) |
| DQ517294 | *Babesia divergens* subtilisin-1 (SUB1) |
| PQ765515 | variant erythrocyte surface antigen-1, alpha subunit (VESA1) |
| PQ765516 | secreted antigen 1 |
| PQ765517 | putative lipoate-protein ligase A |
| PQ765518 | putative translation initiation factor 3 subunit G |
| PQ765519 | heat shock protein 90 (hsp90) |

**S1 Table**. *B. divergens* cDNAs identified in the parasite expression library.
