# Supplementary material for: Elucidating the functional role of the novel BdP50 protein and extracellular vesicles in the human erythrocyte infection by Babesia divergens
Source: PLoS Negl Trop Dis. 2025 Aug 13;19(8):e0013401. doi: 10.1371/journal.pntd.0013401 (PMC12370190; doi:10.1371/journal.pntd.0013401)
Supplement: S2 Table — The table shows the associated gold particles per surface area (GP/µm2) counted in uninfected RBCs (uRBCs) compared to the GP/µm2 counted in infected RBCs (iRBCs). (DOCX) [file pntd.0013401.s011.docx]

**S2 Table**. Quantification of labeling density. The table shows the associated gold particles per surface area (GP/µm2) counted in uninfected RBCs (uRBCs) compared to the GP/µm2 counted in infected RBCs (iRBCs).

| **Sample** | **GP/µm2** (average of individual labeling densities) | **GP/µm2** (labeling density of total GP per total area) |
| --- | --- | --- |
| uRBCs (N=10) | 0,39 | 0,43 |
| iRBCs (N=20) | 6,14 | 4,63 |
