# Supplementary material for: Elucidating the functional role of the novel BdP50 protein and extracellular vesicles in the human erythrocyte infection by Babesia divergens
Source: PLoS Negl Trop Dis. 2025 Aug 13;19(8):e0013401. doi: 10.1371/journal.pntd.0013401 (PMC12370190; doi:10.1371/journal.pntd.0013401)
Supplement: S3 Table — Reference list: Homo sapiens proteome from Uniprot. (DOCX) [file pntd.0013401.s012.docx]

**S3 Table.** Human proteins identified in *Bd*-derived EVs. Reference list: *Homo sapiens* proteome from Uniprot.

| **Protein ID** | **Protein Description** |
| --- | --- |
| A0A0B4J1V0 | Immunoglobulin heavy variable 3-15 |
| O00187 | Mannan-binding lectin serine protease 2 |
| O00231 | 26S proteasome non-ATPase regulatory subunit 11 |
| O00232 | 26S proteasome non-ATPase regulatory subunit 12 |
| O00299 | Chloride intracellular channel protein 1 |
| O00487 | 26S proteasome non-ATPase regulatory subunit 14 |
| O14744 | Protein arginine N-methyltransferase 5 |
| O14791 | Apolipoprotein L1 |
| O14980 | Exportin-1 |
| O15067 | Phosphoribosylformylglycinamidine synthase |
| O15162 | Phospholipid scramblase 1 |
| O43242 | 26S proteasome non-ATPase regulatory subunit 3 |
| O43488 | Aflatoxin B1 aldehyde reductase member 2 |
| O43681 | ATPase GET3 |
| O43813 | Glutathione S-transferase LANCL1 |
| O43866 | CD5 antigen-like |
| O75083 | WD repeat-containing protein 1 |
| O75326 | Semaphorin-7A |
| O75636 | Ficolin-3 |
| O75955 | Flotillin-1 |
| O95373 | Importin-7 |
| O95445 | Apolipoprotein M |
| O95747 | Serine/threonine-protein kinase OSR1 |
| P00352 | Aldehyde dehydrogenase 1A1 |
| P00387 | NADH-cytochrome b5 reductase 3 |
| P00450 | Ceruloplasmin |
| P00558 | Phosphoglycerate kinase 1 |
| P00568 | Adenylate kinase isoenzyme 1 |
| P00734 | Prothrombin |
| P00736 | Complement C1r subcomponent |
| P00738 | Haptoglobin |
| P00739 | Haptoglobin-related protein |
| P00740 | Coagulation factor IX |
| P00747 | Plasminogen |
| P00748 | Coagulation factor XII |
| P00751 | Complement factor B |
| P00761 | Trypsin |
| P00915 | Carbonic anhydrase 1 |
| P00918 | Carbonic anhydrase 2 |
| P01008 | Antithrombin-III |
| P01009 | Alpha-1-antitrypsin |
| P01011 | Alpha-1-antichymotrypsin |
| P01019 | Angiotensinogen |
| P01023 | Alpha-2-macroglobulin |
| P01024 | Complement C3 |
| P01031 | Complement C5 |
| P01042 | Kininogen-1 |
| P01591 | Immunoglobulin J chain |
| P01714 | Immunoglobulin lambda variable 3-19 |
| P01833 | Polymeric immunoglobulin receptor |
| P01834 | Immunoglobulin kappa constant |
| P01857 | Immunoglobulin heavy constant gamma 1 |
| P01859 | Immunoglobulin heavy constant gamma 2 |
| P01860 | Immunoglobulin heavy constant gamma 3 |
| P01861 | Immunoglobulin heavy constant gamma 4 |
| P01871 | Immunoglobulin heavy constant mu |
| P01876 | Immunoglobulin heavy constant alpha 1 |
| P01880 | Immunoglobulin heavy constant delta |
| P02042 | Hemoglobin subunit delta |
| P02533 | Keratin, type I cytoskeletal 14 |
| P02549 | Spectrin alpha chain, erythrocytic 1 |
| P02647 | Apolipoprotein A-I |
| P02649 | Apolipoprotein E |
| P02671 | Fibrinogen alpha chain |
| P02675 | Fibrinogen beta chain |
| P02679 | Fibrinogen gamma chain |
| P02730 | Band 3 anion transport protein |
| P02743 | Serum amyloid P-component |
| P02745 | Complement C1q subcomponent subunit A |
| P02746 | Complement C1q subcomponent subunit B |
| P02747 | Complement C1q subcomponent subunit C |
| P02748 | Complement component C9 |
| P02749 | Beta-2-glycoprotein 1 |
| P02750 | Leucine-rich alpha-2-glycoprotein |
| P02751 | Fibronectin |
| P02760 | Protein AMBP |
| P02765 | Alpha-2-HS-glycoprotein |
| P02766 | Transthyretin |
| P02768 | Albumin |
| P02774 | Vitamin D-binding protein |
| P02787 | Serotransferrin |
| P02788 | Lactotransferrin |
| P02790 | Hemopexin |
| P03951 | Coagulation factor XI |
| P03952 | Plasma kallikrein |
| P04003 | C4b-binding protein alpha chain |
| P04004 | Vitronectin |
| P04040 | Catalase |
| P04083 | Annexin A1 |
| P04114 | Apolipoprotein B-100 |
| P04180 | Phosphatidylcholine-sterol acyltransferase |
| P04217 | Alpha-1B-glycoprotein |
| P04264 | Keratin, type II cytoskeletal 1 |
| P04278 | Sex hormone-binding globulin |
| P04406 | Glyceraldehyde-3-phosphate dehydrogenase |
| P05090 | Apolipoprotein D |
| P05106 | Integrin beta-3 |
| P05109 | Protein S100-A8 |
| P05154 | Plasma serine protease inhibitor |
| P05155 | Plasma protease C1 inhibitor |
| P05156 | Complement factor I |
| P05160 | Coagulation factor XIII B chain |
| P05164 | Myeloperoxidase |
| P05452 | Tetranectin |
| P05543 | Thyroxine-binding globulin |
| P05546 | Heparin cofactor 2 |
| P06276 | Cholinesterase |
| P06312 | Immunoglobulin kappa variable 4-1 |
| P06396 | Gelsolin |
| P06681 | Complement C2 |
| P06727 | Apolipoprotein A-IV |
| P07195 | L-lactate dehydrogenase B chain |
| P07203 | Glutathione peroxidase 1 |
| P07225 | Vitamin K-dependent protein S |
| P07357 | Complement component C8 alpha chain |
| P07360 | Complement component C8 gamma chain |
| P07737 | Profilin-1 |
| P07738 | Bisphosphoglycerate mutase |
| P07996 | Thrombospondin-1 |
| P08185 | Corticosteroid-binding globulin |
| P08237 | ATP-dependent 6-phosphofructokinase, muscle type |
| P08514 | Integrin alpha-IIb |
| P08603 | Complement factor H |
| P08603 | Complement factor H |
| P08603 | Complement factor H |
| P08697 | Alpha-2-antiplasmin |
| P08758 | Annexin A5 |
| P08779 | Keratin, type I cytoskeletal 16 |
| P09543 | 2',3'-cyclic-nucleotide 3'-phosphodiesterase |
| P09871 | Complement C1s subcomponent |
| P0C0L4 | Complement C4-A |
| P0C0L5 | Complement C4-B |
| P10643 | Complement component C7 |
| P10768 | S-formylglutathione hydrolase |
| P10909 | Clusterin |
| P11142 | Heat shock cognate 71 kDa protein |
| P11166 | Solute carrier family 2, facilitated glucose transporter member 1 |
| P11171 | Protein 4.1 |
| P11277 | Spectrin beta chain, erythrocytic |
| P11413 | Glucose-6-phosphate 1-dehydrogenase |
| P11586 | C-1-tetrahydrofolate synthase, cytoplasmic |
| P11678 | Eosinophil peroxidase |
| P12259 | Coagulation factor V |
| P13489 | Ribonuclease inhibitor |
| P13645 | Keratin, type I cytoskeletal 10 |
| P13646 | Keratin, type I cytoskeletal 13 |
| P13647 | Keratin, type II cytoskeletal 5 |
| P13671 | Complement component C6 |
| P13987 | CD59 glycoprotein |
| P14618 | Pyruvate kinase PKM |
| P14868 | Aspartate--tRNA ligase, cytoplasmic |
| P15169 | Carboxypeptidase N catalytic chain |
| P16070 | CD44 antigen |
| P16152 | Carbonyl reductase [NADPH] 1 |
| P16157 | Ankyrin-1 |
| P16452 | Protein 4.2 |
| P17858 | ATP-dependent 6-phosphofructokinase, liver type |
| P17980 | 26S proteasome regulatory subunit 6A |
| P17987 | T-complex protein 1 subunit alpha |
| P18428 | Lipopolysaccharide-binding protein |
| P19256 | Lymphocyte function-associated antigen 3 |
| P19652 | Alpha-1-acid glycoprotein 2 |
| P19823 | Inter-alpha-trypsin inhibitor heavy chain H2 |
| P19827 | Inter-alpha-trypsin inhibitor heavy chain H1 |
| P20073 | Annexin A7 |
| P20742 | Pregnancy zone protein |
| P20851 | C4b-binding protein beta chain |
| P21980 | Protein-glutamine gamma-glutamyltransferase 2 |
| P22303 | Acetylcholinesterase |
| P22314 | Ubiquitin-like modifier-activating enzyme 1 |
| P22352 | Glutathione peroxidase 3 |
| P22792 | Carboxypeptidase N subunit 2 |
| P23142 | Fibulin-1 |
| P23526 | Adenosylhomocysteinase |
| P23528 | Cofilin-1 |
| P25311 | Zinc-alpha-2-glycoprotein |
| P26038 | Moesin |
| P27105 | Stomatin |
| P27169 | Serum paraoxonase/arylesterase 1 |
| P27348 | 14-3-3 protein theta |
| P28066 | Proteasome subunit alpha type-5 |
| P29144 | Tripeptidyl-peptidase 2 |
| P29622 | Kallistatin |
| P30041 | Peroxiredoxin-6 |
| P30043 | Flavin reductase (NADPH) |
| P30153 | Serine/threonine-protein phosphatase 2A 65 kDa regulatory subunit A alpha isoform |
| P30566 | Adenylosuccinate lyase |
| P30613 | Pyruvate kinase PKLR |
| P30626 | Sorcin |
| P31146 | Coronin-1A |
| P31939 | Bifunctional purine biosynthesis protein ATIC |
| P32119 | Peroxiredoxin-2 |
| P35527 | Keratin, type I cytoskeletal 9 |
| P35542 | Serum amyloid A-4 protein |
| P35613 | Basigin |
| P35858 | Insulin-like growth factor-binding protein complex acid labile subunit |
| P35908 | Keratin, type II cytoskeletal 2 epidermal |
| P35998 | 26S proteasome regulatory subunit 7 |
| P36955 | Pigment epithelium-derived factor |
| P36959 | GMP reductase 1 |
| P36980 | Complement factor H-related protein 2 |
| P40227 | T-complex protein 1 subunit zeta |
| P43034 | Platelet-activating factor acetylhydrolase IB subunit beta |
| P43652 | Afamin |
| P43686 | 26S proteasome regulatory subunit 6B |
| P45974 | Ubiquitin carboxyl-terminal hydrolase 5 |
| P46459 | Vesicle-fusing ATPase |
| P48426 | Phosphatidylinositol 5-phosphate 4-kinase type-2 alpha |
| P48556 | 26S proteasome non-ATPase regulatory subunit 8 |
| P48643 | T-complex protein 1 subunit epsilon |
| P48740 | Mannan-binding lectin serine protease 1 |
| P49189 | 4-trimethylaminobutyraldehyde dehydrogenase |
| P49368 | T-complex protein 1 subunit gamma |
| P49913 | Cathelicidin antimicrobial peptide |
| P50395 | Rab GDP dissociation inhibitor beta |
| P50570 | Dynamin-2 |
| P50895 | Basal cell adhesion molecule |
| P50990 | T-complex protein 1 subunit theta |
| P50991 | T-complex protein 1 subunit delta |
| P50995 | Annexin A11 |
| P51665 | 26S proteasome non-ATPase regulatory subunit 7 |
| P51884 | Lumican |
| P52209 | 6-phosphogluconate dehydrogenase, decarboxylating |
| P53004 | Biliverdin reductase A |
| P53396 | ATP-citrate synthase |
| P54578 | Ubiquitin carboxyl-terminal hydrolase 14 |
| P54920 | Alpha-soluble NSF attachment protein |
| P55058 | Phospholipid transfer protein |
| P55072 | Transitional endoplasmic reticulum ATPase |
| P60174 | Triosephosphate isomerase |
| P60842 | Eukaryotic initiation factor 4A-I |
| P60891 | Ribose-phosphate pyrophosphokinase 1 |
| P60900 | Proteasome subunit alpha type-6 |
| P60953 | Cell division control protein 42 homolog |
| P61026 | Ras-related protein Rab-10 |
| P61225 | Ras-related protein Rap-2b |
| P61626 | Lysozyme C |
| P62191 | 26S proteasome regulatory subunit 4 |
| P62195 | 26S proteasome regulatory subunit 8 |
| P62258 | 14-3-3 protein epsilon |
| P62333 | 26S proteasome regulatory subunit 10B |
| P62805 | Histone H4 |
| P62826 | GTP-binding nuclear protein Ran |
| P62834 | Ras-related protein Rap-1A |
| P62873 | Guanine nucleotide-binding protein G(I)/G(S)/G(T) subunit beta-1 |
| P63104 | 14-3-3 protein zeta/delta |
| P68871 | Hemoglobin subunit beta |
| P69905 | Hemoglobin subunit alpha |
| P78371 | T-complex protein 1 subunit beta |
| P78417 | Glutathione S-transferase omega-1 |
| P80108 | Phosphatidylinositol-glycan-specific phospholipase D |
| Q00013 | 55 kDa erythrocyte membrane protein |
| Q00013 | 55 kDa erythrocyte membrane protein |
| Q00577 | Transcriptional activator protein Pur-alpha |
| Q00610 | Clathrin heavy chain 1 |
| Q06033 | Inter-alpha-trypsin inhibitor heavy chain H3 |
| Q08380 | Galectin-3-binding protein |
| Q08495 | Dematin |
| Q08722 | Leukocyte surface antigen CD47 |
| Q13200 | 26S proteasome non-ATPase regulatory subunit 2 |
| Q13228 | Methanethiol oxidase |
| Q13630 | GDP-L-fucose synthase |
| Q14240 | Eukaryotic initiation factor 4A-II |
| Q14254 | Flotillin-2 |
| Q14520 | Hyaluronan-binding protein 2 |
| Q14624 | Inter-alpha-trypsin inhibitor heavy chain H4 |
| Q14974 | Importin subunit beta-1 |
| Q14C86 | GTPase-activating protein and VPS9 domain-containing protein 1 |
| Q15008 | 26S proteasome non-ATPase regulatory subunit 6 |
| Q15102 | Platelet-activating factor acetylhydrolase IB subunit alpha1 |
| Q16401 | 26S proteasome non-ATPase regulatory subunit 5 |
| Q53H96 | Pyrroline-5-carboxylate reductase 3 |
| Q5JWF2 | Guanine nucleotide-binding protein G(s) subunit alpha isoforms XLas |
| Q5T4S7 | E3 ubiquitin-protein ligase UBR4 |
| Q6XQN6 | Nicotinate phosphoribosyltransferase |
| Q86VP6 | Cullin-associated NEDD8-dissociated protein 1 |
| Q86YZ3 | Hornerin |
| Q8IZ83 | Aldehyde dehydrogenase family 16 member A1 |
| Q8TDL5 | BPI fold-containing family B member 1 |
| Q8WUM4 | Programmed cell death 6-interacting protein |
| Q92530 | Proteasome inhibitor PI31 subunit |
| Q96IY4 | Carboxypeptidase B2 |
| Q96KN2 | Beta-Ala-His dipeptidase |
| Q96PD5 | N-acetylmuramoyl-L-alanine amidase |
| Q99460 | 26S proteasome non-ATPase regulatory subunit 1 |
| Q99808 | Equilibrative nucleoside transporter 1 |
| Q99832 | T-complex protein 1 subunit eta |
| Q9BQA1 | Methylosome protein 50 |
| Q9BT78 | COP9 signalosome complex subunit 4 |
| Q9H479 | Fructosamine-3-kinase |
| Q9H4G4 | Golgi-associated plant pathogenesis-related protein 1 |
| Q9HA64 | Ketosamine-3-kinase |
| Q9HDC9 | Adipocyte plasma membrane-associated protein |
| Q9NP59 | Solute carrier family 40 member 1 |
| Q9NTK5 | Obg-like ATPase 1 |
| Q9P2R3 | Rabankyrin-5 |
| Q9UGM5 | Fetuin-B |
| Q9UIA9 | Exportin-7 |
| Q9UK55 | Protein Z-dependent protease inhibitor |
| Q9UNM6 | 26S proteasome non-ATPase regulatory subunit 13 |
| Q9UQ80 | Proliferation-associated protein 2G4 |
| Q9Y230 | RuvB-like 2 |
| Q9Y265 | RuvB-like 1 |
| Q9Y315 | Deoxyribose-phosphate aldolase |
| Q9Y3I1 | F-box only protein 7 |
| Q9Y490 | Talin-1 |
