# Supplementary material for: Elucidating the functional role of the novel BdP50 protein and extracellular vesicles in the human erythrocyte infection by Babesia divergens
Source: PLoS Negl Trop Dis. 2025 Aug 13;19(8):e0013401. doi: 10.1371/journal.pntd.0013401 (PMC12370190; doi:10.1371/journal.pntd.0013401)
Supplement: S4 Table — Reference list: Homo sapiens proteome from Uniprot. (DOCX) [file pntd.0013401.s013.docx]

**Supplementary Table 4**. Human proteins identified in uRBCs-derived EVs replicates. Reference list: *Homo sapiens* proteome from Uniprot.

| **Protein ID** | **Protein Description** |
| --- | --- |
| P31946 | 14-3-3 protein beta/alpha |
| P62258 | 14-3-3 protein epsilon |
| Q04917 | 14-3-3 protein eta |
| P27348 | 14-3-3 protein theta |
| P63104 | 14-3-3 protein zeta/delta |
| P09543 | 2',3'-cyclic-nucleotide 3'-phosphodiesterase |
| Q13200 | 26S proteasome non-ATPase regulatory subunit 2 |
| P08195 | 4F2 cell-surface antigen heavy chain |
| Q00013 | 55 kDa erythrocyte membrane protein |
| P52209 | 6-phosphogluconate dehydrogenase, decarboxylating |
| P22303 | Acetylcholinesterase |
| P61160 | Actin-related protein 2 |
| O15143 | Actin-related protein 2/3 complex subunit 1B |
| O15144 | Actin-related protein 2/3 complex subunit 2 |
| O15145 | Actin-related protein 2/3 complex subunit 3 |
| P59998 | Actin-related protein 2/3 complex subunit 4 |
| P61158 | Actin-related protein 3 |
| P13798 | Acylamino-acid-releasing enzyme |
| P23526 | Adenosylhomocysteinase |
| P00568 | Adenylate kinase isoenzyme 1 |
| P30566 | Adenylosuccinate lyase |
| Q01518 | Adenylyl cyclase-associated protein 1 |
| Q9HDC9 | Adipocyte plasma membrane-associated protein |
| P43652 | Afamin |
| P02768 | Albumin |
| P00352 | Aldehyde dehydrogenase 1A1 |
| P02763 | Alpha-1-acid glycoprotein 1 |
| P19652 | Alpha-1-acid glycoprotein 2 |
| P01011 | Alpha-1-antichymotrypsin |
| P01009 | Alpha-1-antitrypsin |
| P04217 | Alpha-1B-glycoprotein |
| P08697 | Alpha-2-antiplasmin |
| P02765 | Alpha-2-HS-glycoprotein |
| P01023 | Alpha-2-macroglobulin |
| P12814 | Alpha-actinin-1 |
| O43707 | Alpha-actinin-4 |
| P06733 | Alpha-enolase |
| P54920 | Alpha-soluble NSF attachment protein |
| Q9H4A4 | Aminopeptidase B |
| Q01432 | AMP deaminase 3 |
| P01019 | Angiotensinogen |
| P16157 | Ankyrin-1 |
| P04083 | Annexin A1 |
| P50995 | Annexin A11 |
| P07355 | Annexin A2 |
| P12429 | Annexin A3 |
| P09525 | Annexin A4 |
| P08758 | Annexin A5 |
| P08133 | Annexin A6 |
| P20073 | Annexin A7 |
| P01008 | Antithrombin-III |
| P63010 | AP-2 complex subunit beta |
| P02647 | Apolipoprotein A-I |
| P02652 | Apolipoprotein A-II |
| P06727 | Apolipoprotein A-IV |
| P04114 | Apolipoprotein B-100 |
| P05090 | Apolipoprotein D |
| P02649 | Apolipoprotein E |
| O14791 | Apolipoprotein L1 |
| P20292 | Arachidonate 5-lipoxygenase-activating protein |
| Q9NP58 | ATP-binding cassette sub-family B member 6 |
| P53396 | ATP-citrate synthase |
| P17858 | ATP-dependent 6-phosphofructokinase, liver type |
| P20160 | Azurocidin |
| P02730 | Band 3 anion transport protein |
| P50895 | Basal cell adhesion molecule |
| P35613 | Basigin |
| P02749 | Beta-2-glycoprotein 1 |
| P31939 | Bifunctional purine biosynthesis protein ATIC |
| P53004 | Biliverdin reductase A |
| P07738 | Bisphosphoglycerate mutase |
| Q13867 | Bleomycin hydrolase |
| P80723 | Brain acid soluble protein 1 |
| P11586 | C-1-tetrahydrofolate synthase, cytoplasmic |
| P04003 | C4b-binding protein alpha chain |
| P21730 | C5a anaphylatoxin chemotactic receptor 1 |
| Q9Y376 | Calcium-binding protein 39 |
| P27824 | Calnexin |
| P07384 | Calpain-1 catalytic subunit |
| O15484 | Calpain-5 |
| P27797 | Calreticulin |
| P00915 | Carbonic anhydrase 1 |
| P00918 | Carbonic anhydrase 2 |
| P22748 | Carbonic anhydrase 4 |
| P04040 | Catalase |
| P49913 | Cathelicidin antimicrobial peptide |
| P08311 | Cathepsin G |
| Q96F85 | CB1 cannabinoid receptor-interacting protein 1 |
| Q8N6Q3 | CD177 antigen |
| P16070 | CD44 antigen |
| O43866 | CD5 antigen-like |
| P13987 | CD59 glycoprotein |
| P60953 | Cell division control protein 42 homolog |
| P00450 | Ceruloplasmin |
| Q7LBR1 | Charged multivesicular body protein 1b |
| O00299 | Chloride intracellular channel protein 1 |
| Q8IWA5 | Choline transporter-like protein 2 |
| Q00610 | Clathrin heavy chain 1 |
| P10909 | Clusterin |
| P23528 | Cofilin-1 |
| P02746 | Complement C1q subcomponent subunit B |
| P02747 | Complement C1q subcomponent subunit C |
| P00736 | Complement C1r subcomponent |
| P09871 | Complement C1s subcomponent |
| P01024 | Complement C3 |
| P01031 | Complement C5 |
| P13671 | Complement component C6 |
| P07357 | Complement component C8 alpha chain |
| P07358 | Complement component C8 beta chain |
| P02748 | Complement component C9 |
| P08174 | Complement decay-accelerating factor |
| P00751 | Complement factor B |
| P08603 | Complement factor H |
| Q99829 | Copine-1 |
| O75131 | Copine-3 |
| O75367 | Core histone macro-H2A.1 |
| P31146 | Coronin-1A |
| P57737 | Coronin-7 |
| P08185 | Corticosteroid-binding globulin |
| Q9NUQ9 | CYFIP-related Rac1 interactor B |
| P01040 | Cystatin-A |
| P04839 | Cytochrome b-245 heavy chain |
| P13716 | Delta-aminolevulinic acid dehydratase |
| Q08554 | Desmocollin-1 |
| P25686 | DnaJ homolog subfamily B member 2 |
| P50570 | Dynamin-2 |
| Q93070 | Ecto-ADP-ribosyltransferase 4 |
| Q9H4M9 | EH domain-containing protein 1 |
| P11021 | Endoplasmic reticulum chaperone BiP |
| P11678 | Eosinophil peroxidase |
| Q99808 | Equilibrative nucleoside transporter 1 |
| Q96PL5 | Erythroid membrane-associated protein |
| P15311 | Ezrin |
| P52907 | F-actin-capping protein subunit alpha-1 |
| P47756 | F-actin-capping protein subunit beta |
| Q9Y3I1 | F-box only protein 7 |
| Q86UX7 | Fermitin family homolog 3 |
| P02671 | Fibrinogen alpha chain |
| P02675 | Fibrinogen beta chain |
| P02679 | Fibrinogen gamma chain |
| P02751 | Fibronectin |
| Q5D862 | Filaggrin-2 |
| P21333 | Filamin-A |
| P30043 | Flavin reductase (NADPH) |
| O75955 | Flotillin-1 |
| Q14254 | Flotillin-2 |
| O95466 | Formin-like protein 1 |
| P04075 | Fructose-bisphosphate aldolase A |
| P17931 | Galectin-3 |
| Q08380 | Galectin-3-binding protein |
| P60520 | Gamma-aminobutyric acid receptor-associated protein-like 2 |
| Q96QA5 | Gasdermin-A |
| P06396 | Gelsolin |
| P11413 | Glucose-6-phosphate 1-dehydrogenase |
| P00390 | Glutathione reductase, mitochondrial |
| P78417 | Glutathione S-transferase omega-1 |
| P09211 | Glutathione S-transferase P |
| P04406 | Glyceraldehyde-3-phosphate dehydrogenase |
| P06737 | Glycogen phosphorylase, liver form |
| P46976 | Glycogenin-1 |
| Q9H4G4 | Golgi-associated plant pathogenesis-related protein 1 |
| P28676 | Grancalcin |
| P62993 | Growth factor receptor-bound protein 2 |
| P62826 | GTP-binding nuclear protein Ran |
| P04899 | Guanine nucleotide-binding protein G(i) subunit alpha-2 |
| P08754 | Guanine nucleotide-binding protein G(i) subunit alpha-3 |
| P62873 | Guanine nucleotide-binding protein G(I)/G(S)/G(T) subunit beta-1 |
| P62879 | Guanine nucleotide-binding protein G(I)/G(S)/G(T) subunit beta-2 |
| P50148 | Guanine nucleotide-binding protein G(q) subunit alpha |
| Q5JWF2 | Guanine nucleotide-binding protein G(s) subunit alpha isoforms XLas |
| Q14344 | Guanine nucleotide-binding protein subunit alpha-13 |
| P00738 | Haptoglobin |
| P00738 | Haptoglobin |
| P00739 | Haptoglobin-related protein |
| P11142 | Heat shock cognate 71 kDa protein |
| P07900 | Heat shock protein HSP 90-alpha |
| Q9NRV9 | Heme-binding protein 1 |
| Q9NRV9 | Heme-binding protein 1 |
| P69905 | Hemoglobin subunit alpha |
| P68871 | Hemoglobin subunit beta |
| P02042 | Hemoglobin subunit delta |
| P02790 | Hemopexin |
| P05546 | Heparin cofactor 2 |
| O14964 | Hepatocyte growth factor-regulated tyrosine kinase substrate |
| Q14103 | Heterogeneous nuclear ribonucleoprotein D0 |
| P22626 | Heterogeneous nuclear ribonucleoproteins A2/B1 |
| P52790 | Hexokinase-3 |
| P30825 | High affinity cationic amino acid transporter 1 |
| P04196 | Histidine-rich glycoprotein |
| P62805 | Histone H4 |
| P04439 | HLA class I histocompatibility antigen, A alpha chain |
| Q86YZ3 | Hornerin |
| Q14520 | Hyaluronan-binding protein 2 |
| Q16775 | Hydroxyacylglutathione hydrolase, mitochondrial |
| P00492 | Hypoxanthine-guanine phosphoribosyltransferase |
| Q9Y6R7 | IgGFc-binding protein |
| P01876 | Immunoglobulin heavy constant alpha 1 |
| P01857 | Immunoglobulin heavy constant gamma 1 |
| P01859 | Immunoglobulin heavy constant gamma 2 |
| P01860 | Immunoglobulin heavy constant gamma 3 |
| P01861 | Immunoglobulin heavy constant gamma 4 |
| P01871 | Immunoglobulin heavy constant mu |
| A0A0B4J1V0 | Immunoglobulin heavy variable 3-15 |
| P01780 | Immunoglobulin heavy variable 3-7 |
| A0A0B4J1U7 | Immunoglobulin heavy variable 6-1 |
| P01591 | Immunoglobulin J chain |
| P01834 | Immunoglobulin kappa constant |
| P06312 | Immunoglobulin kappa variable 4-1 |
| A0A075B6J9 | Immunoglobulin lambda variable 2-18 |
| P01714 | Immunoglobulin lambda variable 3-19 |
| P23229 | Integrin alpha-6 |
| P08514 | Integrin alpha-IIb |
| P20701 | Integrin alpha-L |
| P11215 | Integrin alpha-M |
| P05107 | Integrin beta-2 |
| P05106 | Integrin beta-3 |
| P19827 | Inter-alpha-trypsin inhibitor heavy chain H1 |
| P19823 | Inter-alpha-trypsin inhibitor heavy chain H2 |
| Q06033 | Inter-alpha-trypsin inhibitor heavy chain H3 |
| Q14624 | Inter-alpha-trypsin inhibitor heavy chain H4 |
| P32942 | Intercellular adhesion molecule 3 |
| O75874 | Isocitrate dehydrogenase [NADP] cytoplasmic |
| P53990 | IST1 homolog |
| Q9Y624 | Junctional adhesion molecule A |
| P23276 | Kell blood group glycoprotein |
| P13645 | Keratin, type I cytoskeletal 10 |
| P02533 | Keratin, type I cytoskeletal 14 |
| P08779 | Keratin, type I cytoskeletal 16 |
| P35527 | Keratin, type I cytoskeletal 9 |
| P04264 | Keratin, type II cytoskeletal 1 |
| Q7Z794 | Keratin, type II cytoskeletal 1b |
| P35908 | Keratin, type II cytoskeletal 2 epidermal |
| P13647 | Keratin, type II cytoskeletal 5 |
| Q5T749 | Keratinocyte proline-rich protein |
| P01042 | Kininogen-1 |
| P02788 | Lactotransferrin |
| P20700 | Lamin-B1 |
| P30740 | Leukocyte elastase inhibitor |
| Q8N423 | Leukocyte immunoglobulin-like receptor subfamily B member 2 |
| Q08722 | Leukocyte surface antigen CD47 |
| P09960 | Leukotriene A-4 hydrolase |
| P18428 | Lipopolysaccharide-binding protein |
| P00338 | L-lactate dehydrogenase A chain |
| P07195 | L-lactate dehydrogenase B chain |
| O60488 | Long-chain-fatty-acid--CoA ligase 4 |
| P24666 | Low molecular weight phosphotyrosine protein phosphatase |
| P51884 | Lumican |
| P19256 | Lymphocyte function-associated antigen 3 |
| P61626 | Lysozyme C |
| P40121 | Macrophage-capping protein |
| Q14764 | Major vault protein |
| P40925 | Malate dehydrogenase, cytoplasmic |
| P14780 | Matrix metalloproteinase-9 |
| Q658P3 | Metalloreductase STEAP3 |
| Q13228 | Methanethiol oxidase |
| P26038 | Moesin |
| P05164 | Myeloperoxidase |
| P60660 | Myosin light polypeptide 6 |
| P35579 | Myosin-9 |
| O14745 | Na(+)/H(+) exchange regulatory cofactor NHE-RF1 |
| Q15758 | Neutral amino acid transporter B(0) |
| P19878 | Neutrophil cytosol factor 2 |
| Q15080 | Neutrophil cytosol factor 4 |
| P80188 | Neutrophil gelatinase-associated lipocalin |
| P43490 | Nicotinamide phosphoribosyltransferase |
| P15531 | Nucleoside diphosphate kinase A |
| Q9NTK5 | Obg-like ATPase 1 |
| Q6UX06 | Olfactomedin-4 |
| Q92882 | Osteoclast-stimulating factor 1 |
| O95497 | Pantetheinase |
| O95498 | Pantetheine hydrolase VNN2 |
| Q99497 | Parkinson disease protein 7 |
| P62937 | Peptidyl-prolyl cis-trans isomerase A |
| Q06830 | Peroxiredoxin-1 |
| P32119 | Peroxiredoxin-2 |
| P30041 | Peroxiredoxin-6 |
| P30086 | Phosphatidylethanolamine-binding protein 1 |
| P48426 | Phosphatidylinositol 5-phosphate 4-kinase type-2 alpha |
| P36871 | Phosphoglucomutase-1 |
| P00558 | Phosphoglycerate kinase 1 |
| P18669 | Phosphoglycerate mutase 1 |
| O15162 | Phospholipid scramblase 1 |
| P36955 | Pigment epithelium-derived factor |
| P03952 | Plasma kallikrein |
| P23634 | Plasma membrane calcium-transporting ATPase 4 |
| P05155 | Plasma protease C1 inhibitor |
| P05154 | Plasma serine protease inhibitor |
| P00747 | Plasminogen |
| P13796 | Plastin-2 |
| P16284 | Platelet endothelial cell adhesion molecule |
| P16671 | Platelet glycoprotein 4 |
| P01833 | Polymeric immunoglobulin receptor |
| P09917 | Polyunsaturated fatty acid 5-lipoxygenase |
| P07737 | Profilin-1 |
| Q8WUM4 | Programmed cell death 6-interacting protein |
| P12273 | Prolactin-inducible protein |
| Q06323 | Proteasome activator complex subunit 1 |
| P25789 | Proteasome subunit alpha type-4 |
| P11171 | Protein 4.1 |
| P16452 | Protein 4.2 |
| P02760 | Protein AMBP |
| Q9UKV8 | Protein argonaute-2 |
| P07237 | Protein disulfide-isomerase |
| Q9UNF0 | Protein kinase C and casein kinase substrate in neurons protein 2 |
| Q9BZQ8 | Protein Niban 1 |
| P26447 | Protein S100-A4 |
| P06703 | Protein S100-A6 |
| P05109 | Protein S100-A8 |
| P06702 | Protein S100-A9 |
| Q92734 | Protein TFG |
| O75695 | Protein XRP2 |
| Q9Y2J8 | Protein-arginine deiminase type-2 |
| P21980 | Protein-glutamine gamma-glutamyltransferase 2 |
| P00734 | Prothrombin |
| P00491 | Purine nucleoside phosphorylase |
| P14618 | Pyruvate kinase PKM |
| P50395 | Rab GDP dissociation inhibitor beta |
| P35241 | Radixin |
| P46940 | Ras GTPase-activating-like protein IQGAP1 |
| Q13576 | Ras GTPase-activating-like protein IQGAP2 |
| Q7LDG7 | RAS guanyl-releasing protein 2 |
| P63000 | Ras-related C3 botulinum toxin substrate 1 |
| P15153 | Ras-related C3 botulinum toxin substrate 2 |
| P61026 | Ras-related protein Rab-10 |
| P61106 | Ras-related protein Rab-14 |
| P62820 | Ras-related protein Rab-1A |
| Q9H0U4 | Ras-related protein Rab-1B |
| Q15286 | Ras-related protein Rab-35 |
| P61020 | Ras-related protein Rab-5B |
| P51148 | Ras-related protein Rab-5C |
| P51149 | Ras-related protein Rab-7a |
| Q92930 | Ras-related protein Rab-8B |
| P62834 | Ras-related protein Rap-1A |
| P61225 | Ras-related protein Rap-2b |
| P08575 | Receptor-type tyrosine-protein phosphatase C |
| P52566 | Rho GDP-dissociation inhibitor 2 |
| P84095 | Rho-related GTP-binding protein RhoG |
| P13489 | Ribonuclease inhibitor |
| P49247 | Ribose-5-phosphate isomerase |
| Q9Y265 | RuvB-like 1 |
| Q9Y230 | RuvB-like 2 |
| O75326 | Semaphorin-7A |
| O95747 | Serine/threonine-protein kinase OSR1 |
| Q15257 | Serine/threonine-protein phosphatase 2A activator |
| P02787 | Serotransferrin |
| P48595 | Serpin B10 |
| P27169 | Serum paraoxonase/arylesterase 1 |
| Q9Y336 | Sialic acid-binding Ig-like lectin 9 |
| Q92783 | Signal transducing adapter molecule 1 |
| P05023 | Sodium/potassium-transporting ATPase subunit alpha-1 |
| P54709 | Sodium/potassium-transporting ATPase subunit beta-3 |
| P11166 | Solute carrier family 2, facilitated glucose transporter member 1 |
| P30626 | Sorcin |
| P27105 | Stomatin |
| Q99536 | Synaptic vesicle membrane protein VAT-1 homolog |
| O15400 | Syntaxin-7 |
| Q15833 | Syntaxin-binding protein 2 |
| O00560 | Syntenin-1 |
| Q9Y490 | Talin-1 |
| Q9ULP9 | TBC1 domain family member 24 |
| P06127 | T-cell surface glycoprotein CD5 |
| P49368 | T-complex protein 1 subunit gamma |
| P50990 | T-complex protein 1 subunit theta |
| P10599 | Thioredoxin |
| P07996 | Thrombospondin-1 |
| P05543 | Thyroxine-binding globulin |
| P37837 | Transaldolase |
| P02786 | Transferrin receptor protein 1 |
| P61586 | Transforming protein RhoA |
| P55072 | Transitional endoplasmic reticulum ATPase |
| P29401 | Transketolase |
| P60174 | Triosephosphate isomerase |
| P00761 | Trypsin |
| Q99816 | Tumor susceptibility gene 101 protein |
| P41240 | Tyrosine-protein kinase CSK |
| P07948 | Tyrosine-protein kinase Lyn |
| P43405 | Tyrosine-protein kinase SYK |
| P29350 | Tyrosine-protein phosphatase non-receptor type 6 |
| P54578 | Ubiquitin carboxyl-terminal hydrolase 14 |
| P45974 | Ubiquitin carboxyl-terminal hydrolase 5 |
| P22314 | Ubiquitin-like modifier-activating enzyme 1 |
| O00160 | Unconventional myosin-If |
| B0I1T2 | Unconventional myosin-Ig |
| Q13336 | Urea transporter 1 |
| Q16851 | UTP--glucose-1-phosphate uridylyltransferase |
| Q9NRW7 | Vacuolar protein sorting-associated protein 45 |
| P46459 | Vesicle-fusing ATPase |
| P08670 | Vimentin |
| P18206 | Vinculin |
| P02774 | Vitamin D-binding protein |
| P07225 | Vitamin K-dependent protein S |
| P04004 | Vitronectin |
| P04275 | von Willebrand factor |
| Q93050 | V-type proton ATPase 116 kDa subunit a 1 |
| P61421 | V-type proton ATPase subunit d 1 |
| O75083 | WD repeat-containing protein 1 |
| O75083 | WD repeat-containing protein 1 |
| Q969T9 | WW domain-binding protein 2 |
| P25311 | Zinc-alpha-2-glycoprotein |
