# Supplementary material for: Elucidating the functional role of the novel BdP50 protein and extracellular vesicles in the human erythrocyte infection by Babesia divergens
Source: PLoS Negl Trop Dis. 2025 Aug 13;19(8):e0013401. doi: 10.1371/journal.pntd.0013401 (PMC12370190; doi:10.1371/journal.pntd.0013401)
Supplement: S5 Table — NUP: number unique peptides, TUS: Total unique spectra. Reference list: B. divergens proteome [9]. (DOCX) [file pntd.0013401.s014.docx]

**S5 Table**. *Babesia divergens* proteins identified in *Bd*-derived EVs: replicate 1 (text in black), replicate 2 (text in red) and replicate (text in blue). NUP: number unique peptides, TUS: Total unique spectra. Reference list: *B*. *divergens* proteome ^8^.

| **Protein ID** | **Description** | **PT** | **NPU** | **TS** | **TSU** |
| --- | --- | --- | --- | --- | --- |
| BDIVROU_0009600.t1.1 0 | Acyl-CoA-binding_domain-containing_protein_1 | 2 | 2 | 2 | 2 |
| BDIVROU_0009600.t1.1 0 |  | 4 | 4 | 4 | 4 |
| BDIVROU_0010500.t1.1 0 | TVP38/TMEM64_family membrane_protein_slr0305 | 4 | 4 | 4 | 4 |
| BDIVROU_0010500.t1.1 0 |  | 3 | 3 | 3 | 3 |
| BDIVROU_0012800.t1.1 0 | Methionine tRNA_ligase_cytoplasmic | 4 | 4 | 6 | 6 |
| BDIVROU_0017400.t1.1 0 | Hypothetical_protein | 3 | 3 | 3 | 3 |
| BDIVROU_0017400.t1.1 0 |  | 5 | 5 | 6 | 6 |
| BDIVROU_0017600.t1.1 0 | Hypothetical_protein | 4 | 4 | 5 | 5 |
| BDIVROU_0017600.t1.1 0 |  | 3 | 3 | 4 | 4 |
| BDIVROU_0018500.t1.1 0 | V-type_proton_ATPase_subunit_E_1 | 3 | 3 | 3 | 3 |
| BDIVROU_0022500.t1.1 0 | Hexokinase | 19 | 19 | 26 | 26 |
| BDIVROU_0022500.t1.1 0 |  | 16 | 16 | 20 | 20 |
| BDIVROU_0024000.t1.1 0 | Uncharacterized_transporter_YrhG | 6 | 6 | 6 | 6 |
| BDIVROU_0024000.t1.1 0 |  | 4 | 4 | 4 | 4 |
| BDIVROU_0025100.t1.1 0 | Hypothetical_protein | 17 | 17 | 59 | 59 |
| BDIVROU_0025100.t1.1 0 |  | 20 | 20 | 75 | 75 |
| BDIVROU_0025100.t1.1 0 |  | 12 | 12 | 17 | 17 |
| BDIVROU_0025300.t1.1 0 | Hypothetical_protein | 2 | 2 | 2 | 2 |
| BDIVROU_0025700.t1.2 0 | Actin* | 13 | 12 | 43 | 40 |
| BDIVROU_0025700.t1.2 0 |  | 14 | 13 | 42 | 40 |
| BDIVROU_0025700.t1.2 0 |  | 15 | 13 | 25 | 21 |
| BDIVROU_0026200.t1.1 0 | Hypothetical_protein | 4 | 4 | 5 | 5 |
| BDIVROU_0026700.t1.1 0 | Hypothetical_protein | 18 | 7 | 20 | 8 |
| BDIVROU_0026700.t1.1 0 |  | 16 | 6 | 19 | 7 |
| BDIVROU_0026700.t1.1 0 |  | 8 | 3 | 9 | 4 |
| BDIVROU_0032200.t1.1 0 | Hypothetical_protein | 2 | 2 | 3 | 3 |
| BDIVROU_0032200.t1.1 0 |  | 2 | 2 | 3 | 3 |
| BDIVROU_0033500.t1.1 0 | Hypothetical_protein | 4 | 4 | 5 | 5 |
| BDIVROU_0033500.t1.1 0 |  | 4 | 4 | 4 | 4 |
| BDIVROU_0034200.t1.1 0 | T-complex_protein_1_subunit_epsilon | 7 | 7 | 7 | 7 |
| BDIVROU_0034200.t1.1 0 |  | 2 | 2 | 2 | 2 |
| BDIVROU_0035200.t1.1 0 | 40S_ribosomal_protein_S11 | 5 | 4 | 5 | 4 |
| BDIVROU_0035200.t1.1 0 |  | 3 | 2 | 4 | 3 |
| BDIVROU_0038500.t1.1 0 | Hypothetical_protein | 2 | 2 | 2 | 2 |
| BDIVROU_0038700.t1.1 0 | GTP-binding_protein_SAR1A | 6 | 6 | 9 | 9 |
| BDIVROU_0038700.t1.1 0 |  | 4 | 4 | 4 | 4 |
| BDIVROU_0039200.t1.1 0 | Ran-specific_GTPase activating_protein | 2 | 2 | 2 | 2 |
| BDIVROU_0039200.t1.1 0 |  | 2 | 2 | 3 | 3 |
| BDIVROU_0039900.t1.1 0 | 23.2_kDa_heat_shock_protein. Bd17_BdHSP20 | 2 | 2 | 2 | 2 |
| BDIVROU_0039900.t1.1 0 |  | 2 | 2 | 4 | 4 |
| BDIVROU_0040400.t1.1 0 | Hypothetical_protein | 6 | 6 | 10 | 10 |
| BDIVROU_0040400.t1.1 0 |  | 5 | 5 | 6 | 6 |
| BDIVROU_0043100.t1.1 0 | Probable_nucleosome_assembly_protein | 2 | 2 | 4 | 4 |
| BDIVROU_0043800.t1.1 0 | Hypothetical_protein | 2 | 2 | 3 | 3 |
| BDIVROU_0043800.t1.1 0 |  | 2 | 2 | 2 | 2 |
| BDIVROU_0043800.t1.1 0 |  | 2 | 2 | 4 | 4 |
| BDIVROU_0046300.t1.1 0 | Acetyl-coenzyme_A_synthetase | 3 | 3 | 4 | 4 |
| BDIVROU_0046300.t1.1 0 |  | 3 | 3 | 4 | 4 |
| BDIVROU_0051300.t1.1 0 | 40S_ribosomal_protein_S24-2 | 4 | 4 | 4 | 4 |
| BDIVROU_0051300.t1.1 0 |  | 3 | 3 | 5 | 5 |
| BDIVROU_0051600.t1.1 0 | Eukaryotic_translation_initiation_factor_3_subunit_B | 5 | 5 | 6 | 6 |
| BDIVROU_0053100.t1.1 0 | 40S_ribosomal_protein_S14 | 3 | 2 | 3 | 2 |
| BDIVROU_0053100.t1.1 0 |  | 3 | 2 | 4 | 2 |
| BDIVROU_0053600.t1.1 0 | 60S_ribosomal_protein_L12-3 | 5 | 5 | 6 | 6 |
| BDIVROU_0053600.t1.1 0 |  | 3 | 3 | 3 | 3 |
| BDIVROU_0054200.t1.1 0 | Hypothetical_protein | 3 | 3 | 3 | 3 |
| BDIVROU_0054700.t1.1 0 | Guanine_nucleotide-binding_protein_subunit_beta-like_protein | 4 | 4 | 4 | 4 |
| BDIVROU_0054700.t1.1 0 |  | 3 | 3 | 4 | 4 |
| BDIVROU_0055200.t1.1 0 | Dynamin-related_protein_3B | 4 | 4 | 5 | 5 |
| BDIVROU_0055200.t1.1 0 |  | 2 | 2 | 4 | 4 |
| BDIVROU_0056200.t1.2 0 | Thrombospondin-related_apical_membrane_protein_TRAP* | 14 | 14 | 17 | 17 |
| BDIVROU_0056200.t1.2 0 |  | 15 | 15 | 16 | 16 |
| BDIVROU_0056800.t1.1 0 | 40S_ribosomal_protein_S9-1 | 2 | 2 | 2 | 2 |
| BDIVROU_0058100.t1.1 0 | Arginine-tRNA_ligase | 6 | 6 | 8 | 8 |
| BDIVROU_0058100.t1.1 0 |  | 7 | 7 | 8 | 8 |
| BDIVROU_0058500.t1.1 0 | 60S_ribosomal_protein_L13a-2 | 3 | 3 | 3 | 3 |
| BDIVROU_0058500.t1.1 0 |  | 4 | 4 | 4 | 4 |
| BDIVROU_0059700.t1.1 0 | Glyceraldehyde-3-phosphate_dehydrogenase | 10 | 9 | 18 | 17 |
| BDIVROU_0059700.t1.1 0 |  | 10 | 9 | 19 | 18 |
| BDIVROU_0059700.t1.1 0 |  | 6 | 6 | 9 | 9 |
| BDIVROU_0061500.t1.1 0 | Exportin-7 | 2 | 2 | 2 | 2 |
| BDIVROU_0064300.t1.1 0 | Rab9_effector_protein_with_kelch_motifs | 3 | 3 | 4 | 4 |
| BDIVROU_0064300.t1.1 0 |  | 2 | 2 | 2 | 2 |
| BDIVROU_0067700.t1.1 0 | 60S_ribosomal_protein_L22_1 | 2 | 2 | 3 | 3 |
| BDIVROU_0067900.t1.1 0 | 26S_proteasome_non-ATPase_regulatory_subunit_6 | 2 | 2 | 2 | 2 |
| BDIVROU_0068000.t1.1 0 | Eukaryotic_translation_initiation_factor_3_subunit_M | 7 | 7 | 10 | 10 |
| BDIVROU_0068000.t1.1 0 |  | 3 | 3 | 3 | 3 |
| BDIVROU_0068500.t1.1 0 |  | 3 | 3 | 3 | 3 |
| BDIVROU_0069000.t1.1 0 | Late_histone_H2B.2.2 | 2 | 2 | 3 | 3 |
| BDIVROU_0069000.t1.1 0 |  | 2 | 2 | 3 | 3 |
| BDIVROU_0070300.t1.1 0 | 40S_ribosomal_protein_S3-1 | 6 | 6 | 12 | 12 |
| BDIVROU_0070300.t1.1 0 |  | 6 | 6 | 9 | 9 |
| BDIVROU_0070300.t1.1 0 |  | 2 | 2 | 2 | 2 |
| BDIVROU_0072200.t1.1 0 | 26S_proteasome_non-ATPase_regulatory_subunit_3 | 2 | 2 | 4 | 4 |
| BDIVROU_0072200.t1.1 0 |  | 2 | 2 | 2 | 2 |
| BDIVROU_0072600.t1.1 0 | Hypothetical_protein | 2 | 2 | 2 | 2 |
| BDIVROU_0072600.t1.1 0 |  | 2 | 2 | 3 | 3 |
| BDIVROU_0073100.t1.1 0 | Eukaryotic_translation_initiation_factor_3_subunit_F | 2 | 2 | 3 | 3 |
| BDIVROU_0073100.t1.1 0 |  | 2 | 2 | 3 | 3 |
| BDIVROU_0073701.t1.2 0 | F-actina binding_protein_or_coronin | 9 | 9 | 11 | 11 |
| BDIVROU_0073701.t1.2 0 |  | 8 | 8 | 10 | 10 |
| BDIVROU_0073701.t1.2 0 |  | 2 | 2 | 2 | 2 |
| BDIVROU_0074500.t1.1 0 | TBC1_domain_family_member_24 | 4 | 4 | 4 | 4 |
| BDIVROU_0074500.t1.1 0 |  | 2 | 2 | 2 | 2 |
| BDIVROU_0075000.t1.1 0 | Hypothetical_protein | 5 | 5 | 6 | 6 |
| BDIVROU_0075000.t1.1 0 |  | 4 | 4 | 6 | 6 |
| BDIVROU_0076200.t1.1 0 | Phosphatidate_phosphatase_PAH2 | 2 | 2 | 2 | 2 |
| BDIVROU_0076400.t1.1 0 | Vesicle associated_membrane_protein-associated_protein_B | 3 | 3 | 3 | 3 |
| BDIVROU_0076400.t1.1 0 |  | 2 | 2 | 2 | 2 |
| BDIVROU_0076900.t1.1 0 | Triosephosphate_isomerase | 3 | 3 | 3 | 3 |
| BDIVROU_0076900.t1.1 0 |  | 3 | 3 | 3 | 3 |
| BDIVROU_0078500.t1.1 0 | Protein_SEY1_homolog | 3 | 3 | 3 | 3 |
| BDIVROU_0078500.t1.1 0 |  | 3 | 3 | 4 | 4 |
| BDIVROU_0080000.t1.1 0 | 60S_ribosomal_protein_L26-1 | 2 | 2 | 2 | 2 |
| BDIVROU_0080400.t1.1 0 | Probable_elongation_factor_1-gamma_1 | 8 | 8 | 9 | 9 |
| BDIVROU_0080400.t1.1 0 |  | 8 | 8 | 10 | 10 |
| BDIVROU_0080400.t1.1 0 |  | 2 | 2 | 2 | 2 |
| BDIVROU_0080500.t1.1 0 | 60S_ribosomal_protein_L6 | 4 | 4 | 5 | 5 |
| BDIVROU_0080500.t1.1 0 |  | 3 | 3 | 3 | 3 |
| BDIVROU_0082000.t1.1 0 | Bifunctional_dihydrofolate_reductase-thymidylate_synthase | 9 | 9 | 18 | 18 |
| BDIVROU_0082000.t1.1 0 |  | 9 | 9 | 14 | 14 |
| BDIVROU_0082000.t1.1 0 |  | 2 | 2 | 2 | 2 |
| BDIVROU_0091600.t1.1 0 | WD_repeat-containing_protein_82 | 5 | 5 | 6 | 6 |
| BDIVROU_0091600.t1.1 0 |  | 4 | 4 | 5 | 5 |
| BDIVROU_0092200.t1.1 0 | Ribose-phosphate_pyrophosphokinase | 3 | 3 | 3 | 3 |
| BDIVROU_0092200.t1.1 0 |  | 3 | 3 | 3 | 3 |
| BDIVROU_0092300.t1.1 0 | Phosphoglycerate_kinase | 15 | 13 | 26 | 24 |
| BDIVROU_0092300.t1.1 0 |  | 13 | 12 | 23 | 22 |
| BDIVROU_0092300.t1.1 0 |  | 8 | 8 | 11 | 11 |
| BDIVROU_0092500.t1.1 0 | 60S_ribosomal_protein_L35 | 3 | 3 | 3 | 3 |
| BDIVROU_0092600.t1.1 0 | 26S_proteasome_non-ATPase_regulatory_subunit_11 | 2 | 2 | 2 | 2 |
| BDIVROU_0093000.t1.1 0 | Stabilin-1. Bd12D3^+^ | 4 | 4 | 6 | 6 |
| BDIVROU_0093000.t1.1 0 |  | 6 | 6 | 9 | 9 |
| BDIVROU_0093000.t1.1 0 |  | 2 | 2 | 3 | 3 |
| BDIVROU_0094400.t1.1 0 | Polyadenylate-binding_protein_cytoplasmic_and_nuclear | 12 | 12 | 15 | 15 |
| BDIVROU_0094400.t1.1 0 |  | 8 | 8 | 11 | 11 |
| BDIVROU_0094400.t1.1 0 |  | 2 | 2 | 2 | 2 |
| BDIVROU_0095100.t1.1 0 | Hypothetical_protein | 3 | 3 | 3 | 3 |
| BDIVROU_0095100.t1.1 0 |  | 2 | 2 | 2 | 2 |
| BDIVROU_0095200.t1.1 0 | Cell_division_control_protein_48_homolog_A | 4 | 3 | 4 | 3 |
| BDIVROU_0095200.t1.1 0 |  | 2 | 2 | 3 | 3 |
| BDIVROU_0095300.t1.1 0 | Cell_division_control_protein_48_homolog_E | 13 | 11 | 16 | 13 |
| BDIVROU_0095300.t1.1 0 |  | 12 | 10 | 14 | 12 |
| BDIVROU_0096510.t1.2 0 | Actin-depolymerizing_factor_ADF_or_cofilin | 4 | 4 | 5 | 5 |
| BDIVROU_0096510.t1.2 0 |  | 5 | 5 | 6 | 6 |
| BDIVROU_0097500.t1.1 0 | Protein_transport_protein_sec31 | 10 | 10 | 11 | 11 |
| BDIVROU_0097500.t1.1 0 |  | 5 | 5 | 5 | 5 |
| BDIVROU_0102200.t1.1 0 | Hypothetical_protein | 2 | 2 | 2 | 2 |
| BDIVROU_0104100.t1.1 0 | GMP_synthase_[glutamine-hydrolyzing] | 4 | 4 | 6 | 6 |
| BDIVROU_0104100.t1.1 0 |  | 4 | 4 | 6 | 6 |
| BDIVROU_0104800.t1.1 0 | Eukaryotic_translation_initiation_factor_2_subunit_alpha | 4 | 4 | 4 | 4 |
| BDIVROU_0104800.t1.1 0 |  | 2 | 2 | 2 | 2 |
| BDIVROU_0104900.t1.1 0 | 40S_ribosomal_protein_SA | 2 | 2 | 3 | 3 |
| BDIVROU_0104900.t1.1 0 |  | 3 | 3 | 3 | 3 |
| BDIVROU_0108300.t1.1 0 | Hypothetical_protein | 13 | 13 | 24 | 24 |
| BDIVROU_0108300.t1.1 0 |  | 14 | 14 | 19 | 19 |
| BDIVROU_0112000.t1.1 0 | Actin-2 | 3 | 3 | 5 | 5 |
| BDIVROU_0112000.t1.1 0 |  | 3 | 3 | 3 | 3 |
| BDIVROU_0112600.t1.1 0 | Phosphomannomutase | 2 | 2 | 2 | 2 |
| BDIVROU_0112800.t1.1 0 | Rhoptry-associated protein 1_RAP-1* | 13 | 0 | 26 | 0 |
| BDIVROU_0112800.t1.1 0 |  | 13 | 0 | 26 | 0 |
| BDIVROU_0112800.t1.1 0 |  | 13 | 0 | 26 | 0 |
| BDIVROU_0113400.t1.1 0 | DnaJ_homolog_subfamily_A_member_1 | 7 | 7 | 9 | 9 |
| BDIVROU_0113400.t1.1 0 |  | 5 | 5 | 7 | 7 |
| BDIVROU_0113400.t1.1 0 |  | 2 | 2 | 2 | 2 |
| BDIVROU_0114800.t1.1 0 | Hypothetical_protein | 2 | 2 | 2 | 2 |
| BDIVROU_0115000.t1.1 0 | Calcium-transporting_ATPase | 23 | 23 | 31 | 31 |
| BDIVROU_0115000.t1.1 0 |  | 19 | 19 | 25 | 25 |
| BDIVROU_0115000.t1.1 0 |  | 4 | 4 | 4 | 4 |
| BDIVROU_0116200.t1.1 0 | Eukaryotic_translation_initiation_factor_3_subunit_C-like_protein | 2 | 2 | 2 | 2 |
| BDIVROU_0116200.t1.1 0 |  | 2 | 2 | 2 | 2 |
| BDIVROU_0116300.t1.1 0 | Dynamin-1-like_protein | 35 | 35 | 46 | 46 |
| BDIVROU_0116300.t1.1 0 |  | 34 | 34 | 41 | 41 |
| BDIVROU_0116300.t1.1 0 |  | 13 | 13 | 16 | 16 |
| BDIVROU_0116900.t1.1 0 | 40S_ribosomal_protein_S25 | 2 | 2 | 2 | 2 |
| BDIVROU_0118200.t1.1 0 | NADP-specific_glutamate_dehydrogenase_(Fragment) | 6 | 6 | 7 | 7 |
| BDIVROU_0118200.t1.1 0 |  | 7 | 7 | 10 | 10 |
| BDIVROU_0119700.t1.1 0 | 60S_ribosomal_protein_L14-2 | 2 | 2 | 2 | 2 |
| BDIVROU_0119700.t1.1 0 |  | 2 | 2 | 3 | 3 |
| BDIVROU_0120000.t1.1 0 | Hypothetical_protein | 5 | 5 | 9 | 9 |
| BDIVROU_0120000.t1.1 0 |  | 4 | 4 | 8 | 8 |
| BDIVROU_0120000.t1.1 0 |  | 2 | 2 | 2 | 2 |
| BDIVROU_0120900.t1.1 0 | Elongation_factor_1-alpha | 19 | 7 | 47 | 15 |
| BDIVROU_0120900.t1.1 0 |  | 19 | 7 | 44 | 14 |
| BDIVROU_0120900.t1.1 0 |  | 15 | 6 | 30 | 10 |
| BDIVROU_0121300.t1.1 0 | Hypothetical_protein | 8 | 8 | 17 | 17 |
| BDIVROU_0121300.t1.1 0 |  | 8 | 8 | 15 | 15 |
| BDIVROU_0121300.t1.1 0 |  | 5 | 5 | 6 | 6 |
| BDIVROU_0123200.t1.1 0 | GTP-binding_nuclear_protein_Ran | 6 | 4 | 7 | 5 |
| BDIVROU_0123200.t1.1 0 |  | 5 | 3 | 6 | 4 |
| BDIVROU_0123200.t1.1 0 |  | 5 | 2 | 6 | 3 |
| BDIVROU_0124700.t1.1 0 | Myosin-12 | 2 | 2 | 3 | 3 |
| BDIVROU_0126000.t1.2 0 | Apical membrane antigen 1_(AMA1)*^+^ | 17 | 17 | 29 | 29 |
| BDIVROU_0126000.t1.2 0 |  | 16 | 16 | 21 | 21 |
| BDIVROU_0126000.t1.2 0 |  | 7 | 7 | 9 | 9 |
| BDIVROU_0128400.t1.1 0 | V-type_proton_ATPase_116_kDa_subunit_a_isoform_4 | 2 | 2 | 3 | 3 |
| BDIVROU_0128400.t1.1 0 |  | 2 | 2 | 2 | 2 |
| BDIVROU_0129400.t1.1 0 | Solute_carrier_family_2_facilitated_glucose_transporter_member_3 | 7 | 2 | 9 | 3 |
| BDIVROU_0129400.t1.1 0 |  | 7 | 3 | 11 | 4 |
| BDIVROU_0130101.t1.2 0 | Calcium dependent protein kinase 4 CDPK-4* | 15 | 15 | 18 | 18 |
| BDIVROU_0130101.t1.2 0 |  | 14 | 14 | 19 | 19 |
| BDIVROU_0130101.t1.2 0 |  | 6 | 6 | 8 | 8 |
| BDIVROU_0132000.t1.1 0 | Hypothetical_protein | 2 | 2 | 2 | 2 |
| BDIVROU_0132000.t1.1 0 |  | 5 | 5 | 6 | 6 |
| BDIVROU_0132200.t1.1 0 | Elongation_factor_1 beta | 7 | 7 | 11 | 11 |
| BDIVROU_0132200.t1.1 0 |  | 7 | 7 | 9 | 9 |
| BDIVROU_0132200.t1.1 0 |  | 2 | 2 | 2 | 2 |
| BDIVROU_0133900.t1.1 0 | Hypothetical_protein | 3 | 3 | 3 | 3 |
| BDIVROU_0134500.t1.1 0 | T-complex_protein_1_subunit_theta | 7 | 7 | 8 | 8 |
| BDIVROU_0134500.t1.1 0 |  | 2 | 2 | 2 | 2 |
| BDIVROU_0138100.t1.1 0 | Solute_carrier_family_35_member_B1 | 2 | 2 | 2 | 2 |
| BDIVROU_0138100.t1.1 0 |  | 3 | 3 | 3 | 3 |
| BDIVROU_0139300.t1.1 0 | Protein_tyrosine_phosphatase_PRL-1 | 6 | 6 | 6 | 6 |
| BDIVROU_0139300.t1.1 0 |  | 5 | 5 | 5 | 5 |
| BDIVROU_0141300.t1.1 0 | Superoxide_dismutase_[Fe] | 2 | 2 | 3 | 3 |
| BDIVROU_0142300.t1.2 0 | Rhoptry_neck_protein_5 (RON5)* | 10 | 10 | 10 | 10 |
| BDIVROU_0142300.t1.2 0 |  | 6 | 6 | 6 | 6 |
| BDIVROU_0142400.t1.1 0 | Hypothetical_protein | 6 | 5 | 6 | 5 |
| BDIVROU_0142400.t1.1 0 |  | 5 | 4 | 10 | 7 |
| BDIVROU_0142400.t1.1 0 |  | 2 | 2 | 2 | 2 |
| BDIVROU_0144000.t1.1 0 | L-lactate_dehydrogenase | 11 | 11 | 31 | 31 |
| BDIVROU_0144000.t1.1 0 |  | 10 | 10 | 20 | 20 |
| BDIVROU_0144000.t1.1 0 |  | 5 | 5 | 9 | 9 |
| BDIVROU_0144300.t1.1 0 | *B. divergens* mRNA for erythrocyte binding protein 37.2 precursor | 9 | 9 | 13 | 13 |
| BDIVROU_0144300.t1.1 0 |  | 10 | 10 | 19 | 19 |
| BDIVROU_0144300.t1.1 0 |  | 5 | 5 | 5 | 5 |
| BDIVROU_0144800.t1.1 0 | Hypothetical_protein | 11 | 7 | 11 | 7 |
| BDIVROU_0144800.t1.1 0 |  | 10 | 6 | 12 | 8 |
| BDIVROU_0144800.t1.1 0 |  | 14 | 8 | 17 | 10 |
| BDIVROU_0146300.t1.1 0 | 40S_ribosomal_protein_S8 | 7 | 7 | 7 | 7 |
| BDIVROU_0146300.t1.1 0 |  | 6 | 6 | 7 | 7 |
| BDIVROU_0146300.t1.1 0 |  | 2 | 2 | 2 | 2 |
| BDIVROU_0146700.t1.1 0 | T-complex_protein_1_subunit_zeta | 11 | 11 | 12 | 12 |
| BDIVROU_0146700.t1.1 0 |  | 3 | 3 | 3 | 3 |
| BDIVROU_0146700.t1.1 0 |  | 2 | 2 | 2 | 2 |
| BDIVROU_0146900.t1.1 0 | V-type_proton_ATPase_catalytic_subunit_A | 6 | 6 | 8 | 8 |
| BDIVROU_0146900.t1.1 0 |  | 5 | 5 | 5 | 5 |
| BDIVROU_0147100.t1.1 0 | Glutamine--fructose-6-phosphate_aminotransferase_[isomerizing] | 5 | 5 | 6 | 6 |
| BDIVROU_0147100.t1.1 0 |  | 4 | 4 | 6 | 6 |
| BDIVROU_0147100.t1.1 0 |  | 2 | 2 | 2 | 2 |
| BDIVROU_0151100.t1.1 0 | Hypothetical_protein | 9 | 9 | 13 | 13 |
| BDIVROU_0151100.t1.1 0 |  | 9 | 9 | 12 | 12 |
| BDIVROU_0151100.t1.1 0 |  | 4 | 4 | 5 | 5 |
| BDIVROU_0152600.t1.1 0 | Protein_transport_protein_Sec24A | 4 | 4 | 4 | 4 |
| BDIVROU_0152600.t1.1 0 |  | 3 | 3 | 3 | 3 |
| BDIVROU_0152900.t1.1 0 | Hypothetical_protein | 6 | 6 | 8 | 8 |
| BDIVROU_0152900.t1.1 0 |  | 7 | 7 | 8 | 8 |
| BDIVROU_0153200.t1.1 0 | Fructose-bisphosphate_aldolase_2 | 12 | 12 | 18 | 18 |
| BDIVROU_0153200.t1.1 0 |  | 13 | 13 | 20 | 20 |
| BDIVROU_0153200.t1.1 0 |  | 8 | 8 | 10 | 10 |
| BDIVROU_0155000.t1.1 0 | N-acetylserotonin_O-methyltransferase-like_protein | 2 | 2 | 3 | 3 |
| BDIVROU_0155000.t1.1 0 |  | 3 | 3 | 3 | 3 |
| BDIVROU_0155100.t1.1 0 | Hypothetical_protein | 18 | 17 | 32 | 31 |
| BDIVROU_0155100.t1.1 0 |  | 17 | 16 | 27 | 26 |
| BDIVROU_0155100.t1.1 0 |  | 4 | 3 | 5 | 4 |
| BDIVROU_0155600.t1.1 0 | Long_chain_acyl-CoA_synthetase_7_peroxisomal | 10 | 10 | 16 | 16 |
| BDIVROU_0155600.t1.1 0 |  | 9 | 9 | 15 | 15 |
| BDIVROU_0155600.t1.1 0 |  | 4 | 4 | 4 | 4 |
| BDIVROU_0158600.t1.1 0 | 2-phosphoglycerate dehydratase (Enolase)^+^ | 20 | 20 | 33 | 33 |
| BDIVROU_0158600.t1.1 0 |  | 20 | 20 | 32 | 32 |
| BDIVROU_0158600.t1.1 0 |  | 3 | 3 | 5 | 5 |
| BDIVROU_0161201.t1.2 0 | Mac/perforin_protein_2 (MAC2*) | 3 | 3 | 3 | 3 |
| BDIVROU_0161201.t1.2 0 |  | 2 | 2 | 2 | 2 |
| BDIVROU_0165301.t1.2 0 | Spherical_body_protein_3 (SBP3)***** | 29 | 29 | 42 | 42 |
| BDIVROU_0165301.t1.2 0 |  | 22 | 22 | 32 | 32 |
| BDIVROU_0165301.t1.2 0 |  | 21 | 21 | 25 | 25 |
| BDIVROU_0167700.t1.1 0 | UMP-CMP_kinase | 2 | 2 | 2 | 2 |
| BDIVROU_0168600.t1.1 0 | Protein_transport_protein_SEC23 | 4 | 4 | 6 | 6 |
| BDIVROU_0168600.t1.1 0 |  | 3 | 3 | 3 | 3 |
| BDIVROU_0169800.t1.1 0 | Protein_ABHD17A | 2 | 2 | 2 | 2 |
| BDIVROU_0170900.t1.1 0 | 23-bisphosphoglycerate-dependent_phosphoglycerate_mutase | 8 | 8 | 11 | 11 |
| BDIVROU_0170900.t1.1 0 |  | 6 | 6 | 9 | 9 |
| BDIVROU_0170900.t1.1 0 |  | 3 | 3 | 3 | 3 |
| BDIVROU_0174700.t1.1 0 | Origin_recognition_complex_subunit_1 | 2 | 2 | 2 | 2 |
| BDIVROU_0175500.t1.1 0 | Hypothetical_protein | 4 | 4 | 6 | 6 |
| BDIVROU_0175500.t1.1 0 |  | 4 | 4 | 7 | 7 |
| BDIVROU_0177100.t1.1 0 | Heat shock_70_kDa_protein_C | 8 | 8 | 8 | 8 |
| BDIVROU_0177100.t1.1 0 |  | 10 | 10 | 11 | 11 |
| BDIVROU_0177100.t1.1 0 |  | 3 | 3 | 3 | 3 |
| BDIVROU_0179700.t1.1 0 | 60S_ribosomal_protein_L8 | 3 | 3 | 3 | 3 |
| BDIVROU_0179700.t1.1 0 |  | 2 | 2 | 2 | 2 |
| BDIVROU_0179900.t1.1 0 | Probable_cytoplasmic_aconitate_hydratase | 2 | 2 | 2 | 2 |
| BDIVROU_0181600.t1.1 0 | Hypothetical_protein | 3 | 3 | 7 | 7 |
| BDIVROU_0181600.t1.1 0 |  | 3 | 3 | 7 | 7 |
| BDIVROU_0181900.t1.1 0 | Alanine tRNA_ligase | 4 | 4 | 4 | 4 |
| BDIVROU_0181900.t1.1 0 |  | 2 | 2 | 3 | 3 |
| BDIVROU_0182900.t1.2 0 | glycosylphosphatidylinositol-anchored surface protein (Bd37)*^+^ | 14 | 14 | 77 | 77 |
| BDIVROU_0182900.t1.2 0 |  | 20 | 20 | 116 | 116 |
| BDIVROU_0182900.t1.2 0 |  | 12 | 12 | 31 | 31 |
| BDIVROU_0183000.t1.2 0 | 50-47 kDa surface protein (BdP50)*^+^ | 18 | 18 | 51 | 51 |
| BDIVROU_0183000.t1.2 0 |  | 18 | 18 | 59 | 59 |
| BDIVROU_0183000.t1.2 0 |  | 8 | 8 | 15 | 15 |
| BDIVROU_0186600.t1.1 0 | Asparagine tRNA_ligase | 10 | 10 | 10 | 10 |
| BDIVROU_0186600.t1.1 0 |  | 5 | 5 | 7 | 7 |
| BDIVROU_0188300.t1.1 0 | ABC_transporter_B_family_member_25_mitochondrial | 4 | 4 | 4 | 4 |
| BDIVROU_0188300.t1.1 0 |  | 2 | 2 | 3 | 3 |
| BDIVROU_0190900.t1.2 0 | Babesia_divergens_Calpain-7 | 3 | 3 | 3 | 3 |
| BDIVROU_0190900.t1.2 0 |  | 3 | 3 | 3 | 3 |
| BDIVROU_0191000.t1.1 0 | Aspartate_aminotransferase_chloroplastic | 5 | 5 | 6 | 6 |
| BDIVROU_0191000.t1.1 0 |  | 3 | 3 | 3 | 3 |
| BDIVROU_0191501.t1.2 0 | rhoptry_neck_protein_4 (RON4)* | 4 | 4 | 4 | 4 |
| BDIVROU_0191501.t1.2 0 |  | 3 | 3 | 4 | 4 |
| BDIVROU_0191800.t1.1 0 | Probable_deoxyhypusine_synthase | 5 | 5 | 5 | 5 |
| BDIVROU_0191800.t1.1 0 |  | 3 | 3 | 4 | 4 |
| BDIVROU_0192500.t1.1 0 | Soluble_inorganic_pyrophosphatase_6_chloroplastic | 2 | 2 | 2 | 2 |
| BDIVROU_0192500.t1.1 0 |  | 2 | 2 | 3 | 3 |
| BDIVROU_0192600.t1.1 0 | Kinetochore_protein_Nuf2 | 2 | 2 | 2 | 2 |
| BDIVROU_0192800.t1.1 0 | Receptor_expression-enhancing_protein_5 | 2 | 2 | 2 | 2 |
| BDIVROU_0194800.t1.1 0 | 60S_ribosomal_protein_L27 | 5 | 4 | 7 | 6 |
| BDIVROU_0194800.t1.1 0 |  | 4 | 3 | 6 | 5 |
| BDIVROU_0195400.t1.1 0 | 60S_ribosomal_protein_L5 | 5 | 5 | 6 | 6 |
| BDIVROU_0195400.t1.1 0 |  | 4 | 4 | 4 | 4 |
| BDIVROU_0195400.t1.1 0 |  | 4 | 4 | 5 | 5 |
| BDIVROU_0195600.t1.1 0 | 60S_ribosomal_protein_L7a-2 | 7 | 7 | 11 | 11 |
| BDIVROU_0195600.t1.1 0 |  | 6 | 6 | 9 | 9 |
| BDIVROU_0195600.t1.1 0 |  | 2 | 2 | 3 | 3 |
| BDIVROU_0196200.t1.1 0 | 60S_ribosomal_protein_L21-B | 4 | 4 | 6 | 6 |
| BDIVROU_0196200.t1.1 0 |  | 4 | 4 | 4 | 4 |
| BDIVROU_0196300.t1.1 0 | Transcription_factor_BTF3 | 3 | 3 | 3 | 3 |
| BDIVROU_0196500.t1.1 0 | 60S_ribosomal_protein_L10 | 2 | 2 | 2 | 2 |
| BDIVROU_0196500.t1.1 0 |  | 2 | 2 | 3 | 3 |
| BDIVROU_0197600.t1.1 0 | Long-chain-fatty-acid--CoA_ligase_5 | 16 | 16 | 26 | 26 |
| BDIVROU_0197600.t1.1 0 |  | 18 | 18 | 28 | 28 |
| BDIVROU_0197600.t1.1 0 |  | 9 | 9 | 11 | 11 |
| BDIVROU_0199800.t1.1 0 | Eukaryotic_initiation_factor_4A | 9 | 7 | 17 | 15 |
| BDIVROU_0199800.t1.1 0 |  | 9 | 8 | 15 | 14 |
| BDIVROU_0199800.t1.1 0 |  | 3 | 2 | 5 | 3 |
| BDIVROU_0199900.t1.1 0 | Eukaryotic_initiation_factor_4A | 4 | 3 | 6 | 5 |
| BDIVROU_0199900.t1.1 0 |  | 5 | 4 | 5 | 4 |
| BDIVROU_0202300.t2.2 0 | Babesia_divergens_vignain_like_protease | 6 | 6 | 7 | 7 |
| BDIVROU_0202300.t2.2 0 |  | 6 | 6 | 9 | 9 |
| BDIVROU_0203200.t1.1 0 | Heat_shock_70_kDa_protein | 27 | 24 | 44 | 38 |
| BDIVROU_0203200.t1.1 0 |  | 26 | 22 | 39 | 32 |
| BDIVROU_0203200.t1.1 0 |  | 20 | 17 | 28 | 24 |
| BDIVROU_0204000.t1.1 0 | Coatomer_subunit_alpha | 2 | 2 | 2 | 2 |
| BDIVROU_0204200.t1.1 0 | Histone_H4 | 4 | 2 | 6 | 3 |
| BDIVROU_0208200.t1.1 0 | 40S_ribosomal_protein_S4_X_isoform | 11 | 11 | 17 | 17 |
| BDIVROU_0208200.t1.1 0 |  | 9 | 9 | 12 | 12 |
| BDIVROU_0208200.t1.1 0 |  | 3 | 3 | 5 | 5 |
| BDIVROU_0211600.t1.1 0 | Eukaryotic_translation_initiation_factor_2_subunit_3 | 2 | 2 | 2 | 2 |
| BDIVROU_0211600.t1.1 0 |  | 2 | 2 | 3 | 3 |
| BDIVROU_0215000.t1.1 0 | Glycerol-3-phosphate_dehydrogenase_1-like_protein | 13 | 13 | 18 | 18 |
| BDIVROU_0215000.t1.1 0 |  | 10 | 10 | 13 | 13 |
| BDIVROU_0215000.t1.1 0 |  | 3 | 3 | 4 | 4 |
| BDIVROU_0215700.t1.1 0 | 60S_acidic_ribosomal_protein_P0 | 5 | 5 | 5 | 5 |
| BDIVROU_0215700.t1.1 0 |  | 3 | 3 | 4 | 4 |
| BDIVROU_0216000.t1.1 0 | Hypothetical_protein | 2 | 2 | 3 | 3 |
| BDIVROU_0216000.t1.1 0 |  | 2 | 2 | 3 | 3 |
| BDIVROU_0216100.t1.1 0 | 26S_proteasome_regulatory_subunit_6A_homolog | 2 | 2 | 2 | 2 |
| BDIVROU_0216800.t1.1 0 | Importin-5 | 8 | 8 | 9 | 9 |
| BDIVROU_0216800.t1.1 0 |  | 5 | 5 | 5 | 5 |
| BDIVROU_0218300.t1.1 0 | Hypothetical_protein | 15 | 15 | 17 | 17 |
| BDIVROU_0218300.t1.1 0 |  | 14 | 14 | 15 | 15 |
| BDIVROU_0218300.t1.1 0 |  | 2 | 2 | 2 | 2 |
| BDIVROU_0220600.t1.1 0 | Peptidyl-prolyl_cis-trans_isomerase | 3 | 3 | 6 | 6 |
| BDIVROU_0220600.t1.1 0 |  | 3 | 3 | 6 | 6 |
| BDIVROU_0221000.t1.1 0 | T-complex_protein_1_subunit_delta | 3 | 3 | 3 | 3 |
| BDIVROU_0224310.t1.2 0 | Ser/Thr_phosphatase_PP1_or_calcineurin* | 20 | 20 | 24 | 24 |
| BDIVROU_0224310.t1.2 0 |  | 19 | 19 | 22 | 22 |
| BDIVROU_0224310.t1.2 0 |  | 5 | 5 | 5 | 5 |
| BDIVROU_0226300.t1.1 0 | 40S_ribosomal_protein_S16 | 3 | 3 | 3 | 3 |
| BDIVROU_0226300.t1.1 0 |  | 3 | 3 | 3 | 3 |
| BDIVROU_0226400.t1.1 0 | 60S_ribosomal_protein_L13-1 | 2 | 2 | 2 | 2 |
| BDIVROU_0227200.t1.1 0 | SRSF_protein_kinase_1 | 2 | 2 | 2 | 2 |
| BDIVROU_0227500.t1.1 0 | 40S_ribosomal_protein_S18 | 3 | 3 | 3 | 3 |
| BDIVROU_0227500.t1.1 0 |  | 3 | 3 | 3 | 3 |
| BDIVROU_0229100.t1.1 0 | guanosine monophosphate reductase (gmp reductase)^+^ | 12 | 12 | 16 | 16 |
| BDIVROU_0229100.t1.1 0 |  | 11 | 11 | 15 | 15 |
| BDIVROU_0229100.t1.1 0 |  | 5 | 5 | 7 | 7 |
| BDIVROU_0230700.t1.1 0 | Hypothetical_protein | 2 | 2 | 3 | 3 |
| BDIVROU_0231300.t1.1 0 | Serine_hydroxymethyltransferase_1 | 17 | 17 | 23 | 23 |
| BDIVROU_0231300.t1.1 0 |  | 15 | 15 | 22 | 22 |
| BDIVROU_0231300.t1.1 0 |  | 6 | 6 | 8 | 8 |
| BDIVROU_0234100.t1.1 0 | 60S_ribosomal_protein_L9 | 6 | 6 | 6 | 6 |
| BDIVROU_0234100.t1.1 0 |  | 4 | 4 | 5 | 5 |
| BDIVROU_0238800.t1.1 0 | Glycine-tRNA_ligase | 2 | 2 | 3 | 3 |
| BDIVROU_0240700.t1.1 0 | Isocitrate_dehydrogenase_[NADP]_mitochondrial | 2 | 2 | 2 | 2 |
| BDIVROU_0242300.t1.1 0 | Phosphoenolpyruvate_carboxykinase_(ATP) | 6 | 6 | 7 | 7 |
| BDIVROU_0242300.t1.1 0 |  | 4 | 4 | 4 | 4 |
| BDIVROU_0242901.t1.2 0 | Myosin-A_(MYOA)* | 3 | 3 | 4 | 4 |
| BDIVROU_0242901.t1.2 0 |  | 4 | 4 | 4 | 4 |
| BDIVROU_0243500.t1.1 0 | 40S_ribosomal_protein_S13-1 | 4 | 4 | 5 | 5 |
| BDIVROU_0243500.t1.1 0 |  | 2 | 2 | 3 | 3 |
| BDIVROU_0245400.t1.1 0 | T-complex_protein_1_subunit_alpha | 7 | 7 | 7 | 7 |
| BDIVROU_0245400.t1.1 0 |  | 4 | 4 | 4 | 4 |
| BDIVROU_0246300.t1.1 0 | Adenylyl_cyclase-associated_protein | 4 | 4 | 6 | 6 |
| BDIVROU_0246300.t1.1 0 |  | 3 | 3 | 5 | 5 |
| BDIVROU_0247600.t1.1 0 | 60S_ribosomal_protein_L19 | 3 | 3 | 3 | 3 |
| BDIVROU_0247600.t1.1 0 |  | 2 | 2 | 2 | 2 |
| BDIVROU_0247900.t1.1 0 | Adenosylhomocysteinase^+^ | 14 | 14 | 18 | 18 |
| BDIVROU_0247900.t1.1 0 |  | 12 | 12 | 17 | 17 |
| BDIVROU_0247900.t1.1 0 |  | 3 | 3 | 3 | 3 |
| BDIVROU_0249500.t1.1 0 | Uncharacterized_protein_C458.02c | 11 | 11 | 11 | 11 |
| BDIVROU_0249500.t1.1 0 |  | 12 | 12 | 12 | 12 |
| BDIVROU_0249500.t1.1 0 |  | 2 | 2 | 3 | 3 |
| BDIVROU_0250100.t1.1 0 | ATPase_ASNA1_homolog | 2 | 2 | 2 | 2 |
| BDIVROU_0253300.t1.1 0 | Protein_transport_protein_sec1 | 4 | 4 | 4 | 4 |
| BDIVROU_0253300.t1.1 0 |  | 3 | 3 | 4 | 4 |
| BDIVROU_0254200.t1.1 0 | Protein_DJ-1_homolog_B | 2 | 2 | 2 | 2 |
| BDIVROU_0254200.t1.1 0 |  | 2 | 2 | 3 | 3 |
| BDIVROU_0258900.t1.1 0 | Vacuolar_protein_sorting/targeting_protein_10 | 4 | 4 | 5 | 5 |
| BDIVROU_0258900.t1.1 0 |  | 4 | 4 | 5 | 5 |
| BDIVROU_0259100.t1.2 0 | Rhoptry_neck_protein_2_(RON2)* | 2 | 2 | 2 | 2 |
| BDIVROU_0259100.t1.2 0 |  | 3 | 3 | 4 | 4 |
| BDIVROU_0260200.t1.1 0 | Hypothetical_protein | 3 | 3 | 3 | 3 |
| BDIVROU_0260200.t1.1 0 |  | 3 | 3 | 3 | 3 |
| BDIVROU_0260500.t1.1 0 | Elongation_factor_2 | 14 | 14 | 18 | 18 |
| BDIVROU_0260500.t1.1 0 |  | 14 | 14 | 19 | 19 |
| BDIVROU_0260500.t1.1 0 |  | 5 | 5 | 5 | 5 |
| BDIVROU_0263800.t1.1 0 | 60S_ribosomal_protein_L3^+^ | 8 | 8 | 12 | 12 |
| BDIVROU_0263800.t1.1 0 |  | 5 | 5 | 7 | 7 |
| BDIVROU_0265600.t1.1 0 | Adenosine_deaminase | 6 | 6 | 7 | 7 |
| BDIVROU_0265600.t1.1 0 |  | 6 | 6 | 7 | 7 |
| BDIVROU_0268800.t1.1 0 | 2-Cys_peroxiredoxin_BAS1_chloroplastic_(Fragment) | 8 | 8 | 10 | 10 |
| BDIVROU_0268800.t1.1 0 |  | 7 | 7 | 13 | 13 |
| BDIVROU_0269200.t1.1 0 | Pyruvate_kinase | 11 | 11 | 15 | 15 |
| BDIVROU_0269200.t1.1 0 |  | 10 | 10 | 15 | 15 |
| BDIVROU_0269600.t1.1 0 | 40S_ribosomal_protein_S15 | 2 | 2 | 2 | 2 |
| BDIVROU_0269700.t1.1 0 | Copper_transporter_6 | 2 | 2 | 2 | 2 |
| BDIVROU_0269700.t1.1 0 |  | 2 | 2 | 2 | 2 |
| BDIVROU_0271500.t1.1 0 | CTP_synthase | 3 | 3 | 3 | 3 |
| BDIVROU_0271500.t1.1 0 |  | 2 | 2 | 2 | 2 |
| BDIVROU_0271900.t1.1 0 | Hypothetical_protein | 8 | 8 | 14 | 14 |
| BDIVROU_0271900.t1.1 0 |  | 8 | 8 | 14 | 14 |
| BDIVROU_0271900.t1.1 0 |  | 3 | 3 | 3 | 3 |
| BDIVROU_0272400.t1.1 0 | cAMP-dependent_protein_kinase_regulatory_subunit | 4 | 4 | 5 | 5 |
| BDIVROU_0272400.t1.1 0 |  | 4 | 4 | 5 | 5 |
| BDIVROU_0272901.t1.2 0 | Gliding_associated_protein_45 (GAP45)* | 2 | 2 | 3 | 3 |
| BDIVROU_0272901.t1.2 0 |  | 3 | 3 | 5 | 5 |
| BDIVROU_0274000.t1.1 0 | Sodium-dependent_phosphate_transporter_2 | 6 | 6 | 8 | 8 |
| BDIVROU_0274000.t1.1 0 |  | 5 | 5 | 7 | 7 |
| BDIVROU_0278701.t1.2 0 | Rhomboid-like_protease_4.1 (ROM4.1)* | 15 | 15 | 29 | 29 |
| BDIVROU_0278701.t1.2 0 |  | 14 | 14 | 32 | 32 |
| BDIVROU_0278701.t1.2 0 |  | 7 | 7 | 10 | 10 |
| BDIVROU_0279300.t1.2 0 | Profilin* | 4 | 4 | 4 | 4 |
| BDIVROU_0279300.t1.2 0 |  | 5 | 5 | 6 | 6 |
| BDIVROU_0279700.t1.1 0 | Putative_sulfate_transporter_YbaR | 3 | 3 | 3 | 3 |
| BDIVROU_0280400.t1.1 0 | 60S_ribosomal_protein_L4-A | 8 | 8 | 10 | 10 |
| BDIVROU_0280400.t1.1 0 |  | 6 | 6 | 6 | 6 |
| BDIVROU_0280400.t1.1 0 |  | 3 | 3 | 3 | 3 |
| BDIVROU_0281000.t1.1 0 | Pyrophosphate fructose 6-phosphate 1-phosphotransferase_subunit_beta_1 | 12 | 12 | 15 | 15 |
| BDIVROU_0281000.t1.1 0 |  | 8 | 8 | 8 | 8 |
| BDIVROU_0281000.t1.1 0 |  | 3 | 3 | 3 | 3 |
| BDIVROU_0281700.t1.1 0 | Leucine-tRNA_ligase_cytoplasmic | 4 | 4 | 5 | 5 |
| BDIVROU_0281700.t1.1 0 |  | 2 | 2 | 2 | 2 |
| BDIVROU_0284000.t1.1 0 | 14-3-3_protein_homolog | 3 | 2 | 5 | 3 |
| BDIVROU_0284000.t1.1 0 |  | 3 | 2 | 4 | 3 |
| BDIVROU_0284000.t1.1 0 |  | 3 | 2 | 4 | 2 |
| BDIVROU_0284500.t1.1 0 | Spondin-1 | 14 | 14 | 16 | 16 |
| BDIVROU_0284500.t1.1 0 |  | 14 | 14 | 18 | 18 |
| BDIVROU_0287200.t1.1 0 | Hypothetical_protein | 5 | 5 | 5 | 5 |
| BDIVROU_0287200.t1.1 0 |  | 2 | 2 | 2 | 2 |
| BDIVROU_0288900.t1.1 0 | Charged_multivesicular_body_protein_2a_homolog_2 | 2 | 2 | 2 | 2 |
| BDIVROU_0288900.t1.1 0 |  | 2 | 2 | 2 | 2 |
| BDIVROU_0290800.t1.1 0 | 60S_ribosomal_protein_L17 | 2 | 2 | 3 | 3 |
| BDIVROU_0290800.t1.1 0 |  | 2 | 2 | 2 | 2 |
| BDIVROU_0291700.t1.1 0 | UPF0587_protein_C1orf123_homolog | 2 | 2 | 2 | 2 |
| BDIVROU_0291700.t1.1 0 |  | 2 | 2 | 2 | 2 |
| BDIVROU_0293000.t1.1 0 | Lipoate-protein_ligase_A^+^ | 2 | 2 | 2 | 2 |
| BDIVROU_0293000.t1.1 0 |  | 3 | 3 | 3 | 3 |
| BDIVROU_0294300.t1.1 0 | Ras-related_protein_Rab-11B | 8 | 7 | 10 | 9 |
| BDIVROU_0294300.t1.1 0 |  | 8 | 6 | 12 | 10 |
| BDIVROU_0297000.t1.1 0 |  | 2 | 2 | 4 | 4 |
| BDIVROU_0297800.t1.1 0 | Inosine-5'-monophosphate_dehydrogenase | 5 | 5 | 7 | 7 |
| BDIVROU_0298600.t1.1 0 | T-complex_protein_1_subunit_eta | 5 | 5 | 7 | 7 |
| BDIVROU_0298600.t1.1 0 |  | 4 | 4 | 4 | 4 |
| BDIVROU_0299100.t1.1 0 | Ras-related_protein_Rab-5C | 2 | 2 | 3 | 3 |
| BDIVROU_0299100.t1.1 0 |  | 4 | 4 | 5 | 5 |
| BDIVROU_0299200.t1.1 0 | Chaperonin_CPN60_mitochondrial | 3 | 2 | 3 | 2 |
| BDIVROU_0300600.t1.1 0 | Ubiquitin-conjugating_enzyme_E2_36 | 3 | 3 | 4 | 4 |
| BDIVROU_0300600.t1.1 0 |  | 2 | 2 | 3 | 3 |
| BDIVROU_0302200.t1.1 0 | Serine-tRNA_ligase | 8 | 8 | 9 | 9 |
| BDIVROU_0302200.t1.1 0 |  | 8 | 8 | 9 | 9 |
| BDIVROU_0304800.t1.1 0 | Eukaryotic_translation_initiation_factor_4G | 2 | 2 | 3 | 3 |
| BDIVROU_0304800.t1.1 0 |  | 2 | 2 | 3 | 3 |
| BDIVROU_0308600.t1.2 0 | *Babesia_divergens*_papain-like_cysteine_protease_2 | 2 | 2 | 3 | 3 |
| BDIVROU_0310600.t1.1 0 | Eukaryotic_translation_initiation_factor_3_subunit_K | 3 | 3 | 4 | 4 |
| BDIVROU_0314600.t1.1 0 | Glucose-6-phosphate_isomerase_cytosolic | 8 | 8 | 10 | 10 |
| BDIVROU_0314600.t1.1 0 |  | 7 | 7 | 9 | 9 |
| BDIVROU_0318600.t1.1 0 | Lysine-tRNA_ligase | 3 | 3 | 4 | 4 |
| BDIVROU_0318600.t1.1 0 |  | 2 | 2 | 2 | 2 |
| BDIVROU_0322100.t1.1 0 | Ubiquitin-like_protein_1 | 2 | 2 | 3 | 3 |
| BDIVROU_0322100.t1.1 0 |  | 2 | 2 | 2 | 2 |
| BDIVROU_0322900.t1.1 0 | Protein_disulfide-isomerase | 2 | 2 | 2 | 2 |
| BDIVROU_0323100.t1.1 0 | 60S_ribosomal_protein_L27a | 3 | 3 | 3 | 3 |
| BDIVROU_0323100.t1.1 0 |  | 2 | 2 | 2 | 2 |
| BDIVROU_0323400.t1.1 0 | Phosphatidylinositol_4-phosphate_5-kinase_4 | 6 | 6 | 10 | 10 |
| BDIVROU_0323400.t1.1 0 |  | 6 | 6 | 9 | 9 |
| BDIVROU_0323400.t1.1 0 |  | 2 | 2 | 2 | 2 |
| BDIVROU_0325100.t1.1 0 | Proliferating_cell_nuclear_antigen | 4 | 4 | 5 | 5 |
| BDIVROU_0325100.t1.1 0 |  | 2 | 2 | 2 | 2 |
| BDIVROU_0326000.t1.1 0 | Proteasome_subunit_beta_type 4_(Fragment) | 9 | 9 | 12 | 12 |
| BDIVROU_0326000.t1.1 0 |  | 6 | 6 | 8 | 8 |
| BDIVROU_0326000.t1.1 0 |  | 2 | 2 | 2 | 2 |
| BDIVROU_0328400.t1.1 0 | Threonylcarbamoyl-AMP_synthase | 2 | 2 | 2 | 2 |
| BDIVROU_0328900.t1.1 0 | Phospholipid-transporting_ATPase_IA | 2 | 2 | 2 | 2 |
| BDIVROU_0329000.t1.1 0 | Clathrin_heavy_chain_1 | 6 | 6 | 6 | 6 |
| BDIVROU_0329000.t1.1 0 |  | 3 | 3 | 4 | 4 |
| BDIVROU_0331300.t1.1 0 | Glutamine-tRNA_ligase | 4 | 4 | 4 | 4 |
| BDIVROU_0331300.t1.1 0 |  | 4 | 4 | 5 | 5 |
| BDIVROU_0332400.t1.1 0 | 60S_ribosomal_protein_L23 | 3 | 2 | 4 | 3 |
| BDIVROU_0332400.t1.1 0 |  | 3 | 2 | 3 | 2 |
| BDIVROU_0333900.t1.1 0 | Hypothetical_protein | 2 | 2 | 3 | 3 |
| BDIVROU_0334000.t1.1 0 | Transcription_elongation_factor_1_homolog | 5 | 5 | 19 | 19 |
| BDIVROU_0334000.t1.1 0 |  | 5 | 5 | 16 | 16 |
| BDIVROU_0334000.t1.1 0 |  | 3 | 3 | 3 | 3 |
| BDIVROU_0335000.t1.1 0 | Hypothetical_protein | 9 | 5 | 11 | 7 |
| BDIVROU_0335000.t1.1 0 |  | 12 | 8 | 17 | 12 |
| BDIVROU_0335000.t1.1 0 |  | 14 | 8 | 19 | 13 |
| BDIVROU_0336800.t1.1 0 | Bis(5'-adenosyl)-triphosphatase | 3 | 3 | 3 | 3 |
| BDIVROU_0339200.t1.2 0 | Gliding_associated_protein (GAP50)* | 4 | 4 | 4 | 4 |
| BDIVROU_0339200.t1.2 0 |  | 3 | 3 | 4 | 4 |
| BDIVROU_0359000.t1.1 0 | 40S_ribosomal_protein_S2 | 6 | 6 | 6 | 6 |
| BDIVROU_0359000.t1.1 0 |  | 2 | 2 | 2 | 2 |
| BDIVROU_0359200.t1.1 0 | Uncharacterized_sugar_kinase_slr0537 | 7 | 7 | 13 | 13 |
| BDIVROU_0359200.t1.1 0 |  | 7 | 7 | 11 | 11 |
| BDIVROU_0359200.t1.1 0 |  | 2 | 2 | 6 | 6 |
| BDIVROU_0366100.t1.1 0 | Hypothetical_protein | 6 | 4 | 6 | 4 |
| BDIVROU_0366100.t1.1 0 |  | 6 | 4 | 6 | 4 |
| BDIVROU_0366100.t1.1 0 |  | 5 | 3 | 8 | 6 |
| BDIVROU_0378800.t1.1 0 | Nascent_polypeptide-associated_complex_subunit_alpha | 2 | 2 | 3 | 3 |
| BDIVROU_0379200.t1.1 0 | Eukaryotic_translation_initiation_factor_3_subunit_E | 7 | 6 | 7 | 6 |
| BDIVROU_0379200.t1.1 0 |  | 4 | 4 | 4 | 4 |
| BDIVROU_0379400.t1.1 0 | cAMP-dependent_protein_kinase_catalytic_subunit_beta | 4 | 3 | 4 | 3 |
| BDIVROU_0379400.t1.1 0 |  | 4 | 3 | 5 | 3 |
| BDIVROU_0379600.t2.1 0 | Serine/threonine-protein_phosphatase_BSL3 | 22 | 12 | 30 | 18 |
| BDIVROU_0379600.t2.1 0 |  | 19 | 12 | 32 | 23 |
| BDIVROU_0382300.t1.1 0 | Hypothetical_protein | 5 | 5 | 10 | 10 |
| BDIVROU_0382300.t1.1 0 |  | 6 | 6 | 11 | 11 |
| BDIVROU_0382300.t1.1 0 |  | 2 | 2 | 3 | 3 |
| BDIVROU_0384500.t1.1 0 | Hypothetical_protein | 3 | 3 | 4 | 4 |
| BDIVROU_0385600.t1.1 0 | Hypothetical_protein | 15 | 14 | 27 | 26 |
| BDIVROU_0385600.t1.1 0 |  | 15 | 14 | 26 | 25 |
| BDIVROU_0385600.t1.1 0 |  | 5 | 5 | 5 | 5 |
| BDIVROU_0385700.t1.1 0 | Long_chain_acyl-CoA_synthetase_7_peroxisomal | 15 | 15 | 25 | 25 |
| BDIVROU_0385700.t1.1 0 |  | 13 | 13 | 24 | 24 |
| BDIVROU_0385700.t1.1 0 |  | 9 | 9 | 10 | 10 |
| BDIVROU_0390800.t1.1 0 | mRNA-decapping_enzyme_subunit_2 | 3 | 3 | 3 | 3 |
| BDIVROU_0390800.t1.1 0 |  | 2 | 2 | 3 | 3 |
| BDIVROU_0390900.t1.1 0 | Protein_PYR1-3 | 7 | 7 | 11 | 11 |
| BDIVROU_0390900.t1.1 0 |  | 13 | 13 | 16 | 16 |
| BDIVROU_0391600.t1.1 0 | Protein_transport_protein_sec13 | 4 | 4 | 5 | 5 |
| BDIVROU_0391600.t1.1 0 |  | 2 | 2 | 2 | 2 |
| BDIVROU_0392600.t1.1 0 | Eukaryotic_translation_initiation_factor_3_subunit_L | 3 | 3 | 5 | 5 |
| BDIVROU_0392700.t1.1 0 | Importin_beta-like_SAD2_homolog | 2 | 2 | 3 | 3 |
| BDIVROU_0392700.t1.1 0 |  | 2 | 2 | 2 | 2 |
| BDIVROU_0392800.t1.1 0 | Syntaxin-2 | 2 | 2 | 2 | 2 |
| BDIVROU_0392800.t1.1 0 |  | 2 | 2 | 2 | 2 |
| BDIVROU_0393600.t1.1 0 | Chaperone_protein_dnaJ_10 | 2 | 2 | 2 | 2 |
| BDIVROU_0393600.t1.1 0 |  | 2 | 2 | 2 | 2 |
| BDIVROU_0394000.t1.1 0 | Hypothetical_protein | 2 | 2 | 2 | 2 |
| BDIVROU_0394500.t1.1 0 | 40S_ribosomal_protein_S7 | 7 | 7 | 8 | 8 |
| BDIVROU_0394500.t1.1 0 |  | 4 | 4 | 5 | 5 |
| BDIVROU_0395000.t1.1 0 | Histone-lysine_N-methyltransferase_SUV39H1 | 2 | 2 | 2 | 2 |
| BDIVROU_0397300.t1.1 0 | ADP-ribosylation_factor | 3 | 2 | 5 | 4 |
| BDIVROU_0397300.t1.1 0 |  | 2 | 2 | 6 | 6 |
| BDIVROU_0397400.t1.1 0 | Heat_shock_protein_90^+^ | 22 | 18 | 35 | 28 |
| BDIVROU_0397400.t1.1 0 | Hypothetical_protein | 22 | 18 | 31 | 27 |
| BDIVROU_0397400.t1.1 0 |  | 15 | 12 | 18 | 14 |
| BDIVROU_0397500.t1.1 0 | Heat_shock_protein_88 | 12 | 12 | 15 | 15 |
| BDIVROU_0397500.t1.1 0 |  | 9 | 9 | 11 | 11 |
| BDIVROU_0397500.t1.1 0 |  | 2 | 2 | 2 | 2 |
| BDIVROU_0398100.t1.1 0 | Hypothetical_protein | 2 | 2 | 2 | 2 |
| BDIVROU_0399800.t1.1 0 | GTP-binding_protein_ryh1 | 8 | 7 | 12 | 10 |
| BDIVROU_0399800.t1.1 0 |  | 7 | 6 | 12 | 9 |
| BDIVROU_0401900.t1.1 0 | 60S_ribosomal_protein_L18a | 5 | 5 | 10 | 10 |
| BDIVROU_0401900.t1.1 0 |  | 5 | 5 | 6 | 6 |
| BDIVROU_0401900.t1.1 0 |  | 2 | 2 | 2 | 2 |
| BDIVROU_0402200.t1.1 0 | 60S_ribosomal_protein_L18 | 5 | 5 | 7 | 7 |
| BDIVROU_0402200.t1.1 0 |  | 4 | 4 | 4 | 4 |
| BDIVROU_0402200.t1.1 0 |  | 3 | 3 | 3 | 3 |
| BDIVROU_0402300.t1.1 0 | Protein_wos2 | 2 | 2 | 3 | 3 |
| BDIVROU_0403100.t1.1 0 | Diacylglycerol_kinase_kappa | 2 | 2 | 2 | 2 |
| BDIVROU_0404600.t1.1 0 | Tubulin_beta_chain | 3 | 2 | 3 | 2 |
| BDIVROU_0404600.t1.1 0 |  | 2 | 2 | 2 | 2 |
| BDIVROU_0407600.t1.1 0 | Uridine_phosphorylase | 2 | 2 | 2 | 2 |
| BDIVROU_0407600.t1.1 0 |  | 2 | 2 | 2 | 2 |
| BDIVROU_0411600.t1.1 0 | Hypothetical_protein | 17 | 17 | 49 | 49 |
| BDIVROU_0411600.t1.1 0 |  | 22 | 22 | 64 | 64 |
| BDIVROU_0411600.t1.1 0 |  | 11 | 11 | 14 | 14 |
| BDIVROU_0413400.t1.1 0 | Hypothetical_protein | 20 | 20 | 31 | 31 |
| BDIVROU_0413400.t1.1 0 |  | 20 | 20 | 25 | 25 |
| BDIVROU_0413400.t1.1 0 |  | 5 | 5 | 7 | 7 |
| BDIVROU_0414000.t1.1 0 | T-complex_protein_1_subunit_gamma | 6 | 6 | 6 | 6 |
| BDIVROU_0414000.t1.1 0 |  | 4 | 4 | 5 | 5 |
| BDIVROU_0415600.t1.1 0 | 60S_ribosomal_protein_L10a | 3 | 3 | 8 | 8 |
| BDIVROU_0415600.t1.1 0 |  | 3 | 3 | 3 | 3 |
| BDIVROU_0417700.t1.1 0 | 40S_ribosomal_protein_S26 | 2 | 2 | 2 | 2 |
| BDIVROU_0417700.t1.1 0 |  | 2 | 2 | 2 | 2 |
| BDIVROU_0417900.t1.1 0 | Hypothetical_protein | 9 | 7 | 32 | 28 |
| BDIVROU_0417900.t1.1 0 |  | 9 | 7 | 35 | 30 |
| BDIVROU_0417900.t1.1 0 |  | 6 | 6 | 10 | 10 |
| BDIVROU_0418000.t1.1 0 | Hypothetical_protein | 6 | 6 | 7 | 7 |
| BDIVROU_0418700.t1.1 0 | Hypothetical_protein | 16 | 16 | 47 | 47 |
| BDIVROU_0418700.t1.1 0 |  | 18 | 18 | 56 | 56 |
| BDIVROU_0418700.t1.1 0 |  | 14 | 14 | 17 | 17 |
| BDIVROU_0421700.t1.1 0 | Obg-like_ATPase_1 | 2 | 2 | 2 | 2 |
| BDIVROU_0422200.t1.1 0 | STI1-like_protein | 3 | 3 | 3 | 3 |
| BDIVROU_0426800.t1.1 0 | Ribonuclease_P_protein_subunit_p25-like_protein | 2 | 2 | 2 | 2 |
| BDIVROU_0426800.t1.1 0 |  | 2 | 2 | 2 | 2 |
| BDIVROU_0427500.t1.1 0 | Hypothetical_protein | 13 | 13 | 17 | 17 |
| BDIVROU_0427500.t1.1 0 |  | 13 | 13 | 21 | 21 |
| BDIVROU_0427500.t1.1 0 |  | 4 | 4 | 5 | 5 |
| BDIVROU_0430000.t1.1 0 | 60S_ribosomal_protein_L23a | 3 | 3 | 3 | 3 |
| BDIVROU_0430000.t1.1 0 |  | 2 | 2 | 2 | 2 |
| BDIVROU_0431200.t1.1 0 | 40S_ribosomal_protein_S3a | 11 | 11 | 14 | 14 |
| BDIVROU_0431200.t1.1 0 |  | 10 | 10 | 12 | 12 |
| BDIVROU_0431200.t1.1 0 |  | 3 | 3 | 4 | 4 |
| BDIVROU_0434200.t1.1 0 | Rab_GDP_dissociation_inhibitor_alpha | 2 | 2 | 2 | 2 |
| BDIVROU_0434300.t1.1 0 | 40S_ribosomal_protein_S19-A | 6 | 6 | 7 | 7 |
| BDIVROU_0434300.t1.1 0 |  | 4 | 4 | 6 | 6 |
| BDIVROU_0434400.t1.1 0 | 40S_ribosomal_protein_S12 | 3 | 3 | 5 | 5 |
| BDIVROU_0434400.t1.1 0 |  | 3 | 3 | 3 | 3 |
| BDIVROU_0435000.t1.1 0 | Uncharacterized_protein_At4g15545 | 5 | 5 | 11 | 11 |
| BDIVROU_0435000.t1.1 0 |  | 4 | 4 | 13 | 13 |
| BDIVROU_0435000.t1.1 0 |  | 2 | 2 | 5 | 5 |
| BDIVROU_0435300.t1.1 0 | 60S_ribosomal_protein_L7_2 | 6 | 6 | 9 | 9 |
| BDIVROU_0435300.t1.1 0 |  | 5 | 5 | 5 | 5 |
| BDIVROU_0435400.t1.1 0 | DNA-directed_RNA_polymerase_III_subunit_RPC2 | 4 | 4 | 4 | 4 |
| BDIVROU_0436000.t1.1 0 | Hypothetical_protein | 2 | 2 | 3 | 3 |
| BDIVROU_0436000.t1.1 0 |  | 2 | 2 | 2 | 2 |
| BDIVROU_0436300.t1.1 0 | T-complex_protein_1_subunit_beta | 8 | 8 | 9 | 9 |
| BDIVROU_0436300.t1.1 0 |  | 8 | 8 | 9 | 9 |
| BDIVROU_0436900.t1.1 0 | Ras-related_protein_Rab-1 | 6 | 5 | 6 | 5 |
| BDIVROU_0436900.t1.1 0 |  | 8 | 7 | 8 | 7 |
| BDIVROU_0438100.t1.1 0 | Uncharacterized_Nudix_hydrolase_orf19 | 5 | 5 | 6 | 6 |
| BDIVROU_0438100.t1.1 0 |  | 5 | 5 | 6 | 6 |
| BDIVROU_0439100.t1.1 0 | 60S_ribosomal_protein_L15 | 4 | 4 | 5 | 5 |
| BDIVROU_0439100.t1.1 0 |  | 4 | 4 | 4 | 4 |
| BDIVROU_0439800.t1.1 0 | Eukaryotic_translation_initiation_factor_3_subunit_D | 4 | 3 | 5 | 4 |
| BDIVROU_0444900.t1.1 0 | 40S_ribosomal_protein_S5-2 | 5 | 4 | 5 | 4 |
| BDIVROU_0456000.t1.1 0 | 60S_ribosomal_protein_L36-A | 4 | 3 | 4 | 3 |
| BDIVROU_0456000.t1.1 0 |  | 3 | 3 | 3 | 3 |

* The asterisk represents proteins involved in the invasion of the parasite into the erythrocyte. ^+^ The cross represents the molecules found in the screening of the expression gene library. ID: accession number, PT: Total peptides, NPU: number unique peptides, TS: Total Spectral, TSU: Total unique spectra.
